# Supplementary material for: Night shift work, chemical coexposures and risk of female breast cancer in the Norwegian Offshore Petroleum Workers (NOPW) cohort: a prospectively recruited case-cohort study
Source: BMJ Open. 2022 Jan 24;12(1):e056396. doi: 10.1136/bmjopen-2021-056396 (PMC8788313; doi:10.1136/bmjopen-2021-056396)
Supplement: Supplementary data [file bmjopen-2021-056396supp001.pdf]

SUPPLEMENTAL MATERIAL

Content:

- Methods' extension on multiple imputations
- Table S1
- Table S2
- Table S3
- Table S4
- Table S5
- Table S6
- Directed acyclic graphs (DAGs)
    - Figure S1
    - Figure S2

Liu FC, *et al. BMJ Open* 2022; 12:e056396. doi: 10.1136/bmjopen-2021-056396

Methods' extension

Multiple imputations

Complete case analysis (n=367)

As shown in Figure 1, the study sample for our complete case analysis included 49 breast cancer cases (with complete information on all variables in the regression model).

Analysis of imputed data (n=600)

As shown in Figure 1, our study sample yielded 86 breast cancer cases when those missing data retained. In this sample, 39% (n=233) had missing data on one or more of the following variables (missing numbers are displayed per variable):

- Work schedule involving night/rollover shift: n=32
- Duration of night/rollover shift: n=32
- Number of children: n=8
- Age at first child: n=192
- Education: n=8
- Main occupational activity in last position: n=7

Assuming missing at random, we used multiple imputation with chained equations and followed a 2-steps procedure:

1. We identified differences between these samples for date of birth, total employment duration, and breast cancer diagnosis, and used these variables as predictors together with the variables from the estimation model (i.e. work schedule involving night/rollover shift, duration of night/rollover shift, number of children, age at first child, education, and main

occupational activity in last position) to simultaneously predict missing data on all variables. Linear and multinomial logit models were used to predict missing data for continuous and categorical variables, respectively. The imputation model was ran 45 times, using the rule of thumb of running at least as many times as the percentage of missing data (White et al., 2011).

2. After step 1, we replicated the complete case analyses shown in Table 2, Table 3, and Table 4 on the imputed dataset (n=600).

In a sensitivity analysis (Table S4), we also tried an alternative imputation model where we excluded the variable 'age at first child', which had the highest number of missing (n=192, 39%). This reduced the total number of missing to n=53 (9%), but did not changed the results materially (Table S4).

Reference

White IR, Royston P, Wood AM. Multiple imputation using chained equations: Issues and guidance for practice. Statistics in medicine 2011; 30: 377-99.

Table S1

Table S1. Hazard Ratios of Female Breast Cancer According to Work Schedule in the Norwegian Offshore Petroleum Workers (NOPW) Cohort, sensitivity analyses with menopause cut-off at age ≥51

	Complete case analysis (n=367)			Multiple imputation (n=600; 86 cases)
**Work schedule variable**	**No. of participants**	**No. of cases**	**HR (95% CI)**	**HR (95% CI)**
Total employment duration [a,b]				
Quartile 1 (0-1.9 years)	79	9	1.00 (reference)	1.00 (reference)
Quartile 2 (2-5.9 years)	99	16	1.62 (0.59, 4.42)	1.48 (0.68, 3.23)
Quartile 3 (6-10.9 years)	90	8	0.69 (0.24, 2.00)	1.61 (0.77, 3.38)
Quartile 4 (11-24 years)	99	16	1.19 (0.46, 3.04)	1.17 (0.54, 2.55)
P-trend [c]			0.834	0.765
Work schedule involving night/rollover shift [a,b]				
Unexposed (day work only)	217	29	1.00 (reference)	1.00 (reference)
Exposed (night/rollover shift)	150	20	1.05 (0.57, 1.96)	0.86 (0.51, 1.45)
Duration of night/rollover shift [a,b]				
Unexposed (0 years, day work only)	217	29	1.00 (reference)	1.00 (reference)
≤Median night/rollover (<1-6 years)	68	6	0.71 (0.27, 1.86)	0.55 (0.25, 1.21)
>Median night/rollover (>6 years)	82	14	1.33 (0.66, 2.68)	1.21 (0.67, 2.18)
P-trend [c]			0.543	0.753

Abbreviations: No=number, HR=hazard ratio, CI=confidence interval

[a] Adjusted for age, age at first child, number of children, menopause status (≥51 years as cut-off), and education

[b] complete work history i.e. up to 8 employments as an offshore worker

[c] Modelled as a continuous variable to test for linear trend.

Table S2

Table S2. Hazard Ratios of Female Breast Cancer by Receptor Status Subtypes in Relation to Work Schedule in the Norwegian Offshore Petroleum Workers (NOPW) Cohort, sensitivity analyses with menopause cut-off at age ≥51

		Complete case analysis (n=367)			Multiple imputation (n=600; 86 cases)
**Breast cancer subtype**	**Work schedule variable**	**No. of participants**	**No. of cases**	**HR (95% CI)**	**HR (95% CI)**
ER-positive	Work schedule involving night/rollover shift [a]				
	Unexposed (day work only)	217	23	1.00 (reference)	1.00 (reference)
	Exposed (night/rollover shift)	150	16	1.00 (0.50, 2.01)	0.74 (0.41, 1.34)
ER-negative	Work schedule involving night/rollover shift [a]				
	Unexposed (day work only)	217	5	1.00 (reference)	1.00 (reference)
	Exposed (night/rollover shift)	150	2	0.76 (0.14, 4.17)	1.53 (0.50, 4.70)
PR-positive	Work schedule involving night/rollover shift [a]				
	Unexposed (day work only)	217	25	1.00 (reference)	1.00 (reference)
	Exposed (night/rollover shift)	150	13	0.79 (0.38, 1.64)	0.66 (0.35, 1.24)
PR-negative	Work schedule involving night/rollover shift [a]				
	Unexposed (day work only)	217	3	1.00 (reference)	1.00 (reference)
	Exposed (night/rollover shift)	150	5	2.08 (0.55, 7.88)	1.72 (0.59, 5.02)
HER2-positive	Work schedule involving night/rollover shift [a]				
	Unexposed (day work only)	217	7	1.00 (reference)	1.00 (reference)
	Exposed (night/rollover shift)	150	1	0.27 (0.04, 1.91)	0.21 (0.05, 0.98)
HER2-negative	Work schedule involving night/rollover shift [a]				
	Unexposed (day work only)	217	21	1.00 (reference)	1.00 (reference)
	Exposed (night/rollover shift)	150	14	1.04 (0.50, 2.16)	0.88 (0.48, 1.62)
ER-positive and PR-positive and HER2-negative	Work schedule involving night/rollover shift [a]				
	Unexposed (day work only)	217	17	1.00 (reference)	1.00 (reference)
	Exposed (night/rollover shift)	150	9	0.80 (0.33, 1.94)	0.58 (0.26, 1.28)
ER-positive and PR-positive and HER2-positive	Work schedule involving night/rollover shift [a]				
	Unexposed (day work only)	217	5	1.00 (reference)	1.00 (reference)
	Exposed (night/rollover shift)	150	1	0.35 (0.05, 2.36)	0.27 (0.06, 1.26)
ER-negative and PR-negative and HER2-positive	Work schedule involving night/rollover shift [a]				
	Unexposed (day work only)	217	0	1.00 (reference)	1.00 (reference)
	Exposed (night/rollover shift)	150	0	-	-
ER-negative and PR-negative and HER2-negative	Work schedule involving night/rollover shift [a]				
	Unexposed (day work only)	217	3	1.00 (reference)	1.00 (reference)
	Exposed (night/rollover shift)	150	2	1.34 (0.17, 11)	2.22 (0.58, 8.45)

Abbreviations: No=number, HR=hazard ratio, CI=confidence interval, ER=oestrogen receptor, PR=progesterone receptor, HER2=human epidermal growth factor receptor 2

[a] Adjusted for age, age at first child, number of children, menopause status (≥51 years as cut-off), and education

Liu FC, *et al. BMJ Open* 2022; 12:e056396. doi: 10.1136/bmjopen-2021-056396

Table S3

Table S3. Hazard Ratios of Female Breast Cancer According to Work Schedule in the Norwegian Offshore Petroleum Workers (NOPW) Cohort. Sensitivity Analyses of models adjusted for age at first child *versus* models without adjustment for age at first child.

Work schedule variable	Multiple imputation, including 'age at first child' (n=600; 86 cases) [a,b]<br>HR (95% CI)	Multiple imputation, excluding 'age at first child' (n=600; 86 cases) [b,c]<br>HR (95% CI)
Total employment duration		
Quartile 1 (0-1.9 years)	1.00 (reference)	1.00 (reference)
Quartile 2 (2-5.9 years)	1.50 (0.69, 3.27)	1.53 (0.71, 3.31)
Quartile 3 (6-10.9 years)	1.64 (0.78, 3.50)	1.65 (0.78, 3.47)
Quartile 4 (11-24 years)	1.19 (0.54, 2.61)	1.22 (0.56, 2.67)
P-trend [d1]	0.733	0.685
Work schedule involving night/rollover shift		
Unexposed (day work only)	1.00 (reference)	1.00 (reference)
Exposed (night/rollover shift)	0.87 (0.52, 1.46)	0.87 (0.52, 1.45)
Duration of night/rollover shift		
Unexposed (0 years, day work only)	1.00 (reference)	1.00 (reference)
≤Median night/rollover (<1-6 years)	0.56 (0.26, 1.22)	0.55 (0.25, 1.19)
>Median night/rollover (>6 years)	1.21 (0.67, 2.18)	1.21 (0.67, 2.17)
P-trend [d2]	0.748	0.752

Abbreviations: HR=hazard ratio, CI=confidence interval

[a] Adjusted for age, age at first child, number of children, menopause status (≥53 years as cut-off), and education. 39% (n=233) missing data imputed.

[b] complete work history i.e. up to 8 employments as an offshore worker

[c] Adjusted for variable in footnote "a" but without age at first child. 9% (n=53) missing data imputed.

[d1] Modelled as a continuous variable to test for linear trend. HR for excluding 'age at first child' 1.05 (95% CI: 0.84-1.31).

[d2] Modelled as a continuous variable to test for linear trend. HR for excluding 'age at first child' 1.05 (95% CI: 0.77-1.44).

Liu FC, *et al. BMJ Open* 2022; 12:e056396. doi: 10.1136/bmjopen-2021-056396

Table S4

Table S4. Characteristics of Female Breast Cancer Cases in the Norwegian Offshore Petroleum Workers (NOPW) Cohort.

	**Breast Cancer Cases [a] (n=86)**
Age at diagnosis, mean (range)	56 (38-80)
Clinical stage, n (%)	
I: No metastasis	38 (44)
II: Regional lymph node metastasis	29 (34)
III: Local infiltration into skin	12 (14)
IV: Distant metastasis	4 (5)
Unspecified	3 (3)
Oestrogen receptors (ER), n (%)	
ER-positive	67 (78)
ER-negative	13 (15)
Missing	6 (7)
Progesterone receptors (PR), n (%)	
PR-positive	59 (69)
PR-negative	21 (24)
Missing	6 (7)
Human epidermal growth factor receptor-2 (HER2), n (%)	
HER2-positive	12 (14)
HER2-negative	60 (70)
Missing	14 (16)
ER-positive and PR-positive and HER2-negative, n (%)	
Yes	39 (45)
No	30 (35)
Missing	17 (20)
ER-positive and PR-positive and HER2-positive, n (%)	
Yes	9 (10)
No	60 (70)
Missing	17 (20)
ER-negative and PR-negative and HER2-positive, n (%)	
Yes	0 (0)
No	69 (80)
Missing	17 (20)
ER-negative and PR-negative and HER2-negative	
Yes	11 (13)
No	58 (67)
Missing	17 (20)

[a] According to International Classification of Diseases 10th edition : C50

Liu FC, et al. BMJ Open 2022; 12:e056396. doi: 10.1136/bmjopen-2021-056396

Table S5

Table S5. Hazard Ratios of Breast Cancer According to duration of night + rollover shift quartile in the Norwegian Offshore Petroleum Workers (NOPW) Cohort

	Complete case analysis (n=367)			Multiple imputation (n=600; 86 cases)
**Work schedule variable**	**No. of participants**	**No. of cases**	**HR (95% CI)**	**HR (95% CI)**
Duration of night/rollover shift [a,b]				
Unexposed (0 years, day work only)	217	29	1.00 (reference)	1.00 (reference)
Quartile 1 night/rollover (<1-2 years)	28	3	0.99 (0.28, 3.52)	0.78 (0.28, 2.14)
Quartile 2 night/rollover (>2-5 years)	32	2	0.52 (0.11, 2.47)	0.49 (0.14, 1.71)
Quartile 3 night/rollover (>5-10 years)	36	4	0.77 (0.24, 2.48)	1.02 (0.47, 2.24)
Quartile 4 night/rollover (>10 years)	54	11	1.62 (0.73, 3.59)	1.10 (0.51, 2.37)
P-trend [c]			0.506	0.944

Abbreviations: No=number, HR=hazard ratio, CI=confidence interval

[a] Adjusted for age, age at first child, number of children, menopause status (≥53 years as cut-off), and education

[b] complete work history i.e. up to 8 employments as an offshore worker

[c] Modelled as a continuous variable to test for linear trend

Liu FC, *et al. BMJ Open* 2022; 12:e056396. doi: 10.1136/bmjopen-2021-056396

Table S6

Table S6. Hazard Ratios of Breast Cancer According to Work Schedule and Chemical Co-exposures in the Norwegian Offshore Petroleum Workers (NOPW) Cohort, multiple imputation analysis (n=601)

	Chlorinated degreasers [a]		
**Work schedule variable [b,d]**	**Unexposed**	**Exposed**	**P Value**
Unexposed (day work only)			
n (%)	99 (17)	223 (39)	
HR [c] (95% CI)	1.00 (reference)	0.91 (0.45, 1.84)	
Exposed (night/rollover shift)			
n (%)	43 (8)	204 (36)	
HR [c] (95% CI)	1.01 (0.40, 2.55)	0.79 (0.37, 1.71)	
$P_{interaction}$			0.725
	**Benzene**		
**Work schedule variable [b,d]**	**Unexposed**	**Exposed**	**P Value**
Unexposed (day work only)			
n (%)	269 (48)	53 (9)	
HR [c] (95% CI)	1.00 (reference)	0.60 (0.21, 1.70)	
Exposed (night/rollover shift)			
n (%)	199 (35)	48 (8)	
HR [c] (95% CI)	0.74 (0.41, 1.32)	1.11 (0.46, 2.70)	
$P_{interaction}$			0.175

Abbreviations: HR=hazard ratio, CI=confidence interval

[a] Importantly, the exposure assessment of chlorinated degreasers was probability-based by experts and evaluated as “possible” for catering workers.

[b] Complete work history i.e. up to 8 employments as an offshore worker

[c] Adjusted for age, age at first child, number of children, menopause status, and education

[d] Missing: n=32 for both chlorinated degreasers and benzene.

Liu FC, *et al. BMJ Open* 2022; 12:e056396. doi: 10.1136/bmjopen-2021-056396

Directed acyclic graphs (DAGs)

DAGs were produced by the DAGitty v3.0 software (Textor et al., 2016). Please note that the green circle denotes the exposure, the blue circle with I denotes the outcome, the red circles denote the ancestor of the exposure AND the outcome (i.e. confounders, which are included in the models), and blue circles denote ancestor of the outcome (not included in the models).

<u>Reference</u>

Textor J, Zander B, Gilthorpe MK, Liskiewicz M, Ellison G. Robust causal inference using directed acyclic graphs: the R package 'dagitty'. Int J Epidemiol. 2016;45(6):1887-1894.
Website: www.dagitty.net

<u>Models estimating breast cancer risk in relation to work schedule</u>

In the following, we show DAG for each model with breast cancer as the outcome (as displayed in Tables 2 and 4) in relation to work schedule, age, menopausal status, age at first child, number of children, and education. We made the following assumptions:

- Age and education were not caused by any of the other variables in the DAG
- Menopausal status was affected by age
- Age at first child was affected by education
- Number of children was affected by age at first child

Liu FC, *et al. BMJ Open* 2022; 12:e056396. doi: 10.1136/bmjopen-2021-056396

Figure S1

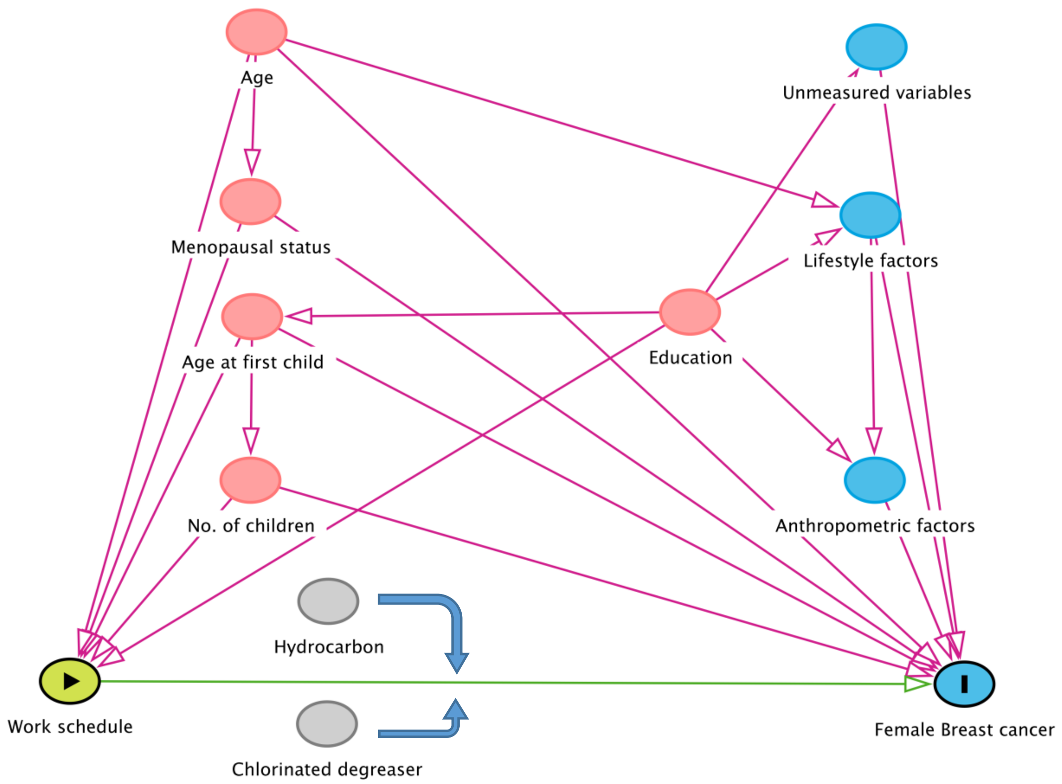

**Lifestyle factors:**	**Antropometric factors:**	**Unmeasured variables:**
Fibre intake	Height	Chronotype
Multivitamine intake	Weight	Exogenous hormones use
Dietary fat intake		Breast feeding
Alcohol intake		Family history
Smoking		
Physical activity		

Models estimating breast cancer risk in relation to chemical co-exposures

In the following, we show DAG for each model with breast cancer as the outcome (as displayed in Tables 3) in relation to age, education, main occupational activity in last position. We made the following assumptions:

- Age, and education were not caused by any of the other variables in the DAG
- Main occupational activity in last position was affected by education, and is an instrument of chemical co-exposure.

Liu FC, *et al. BMJ Open* 2022; 12:e056396. doi: 10.1136/bmjopen-2021-056396

Figure S2

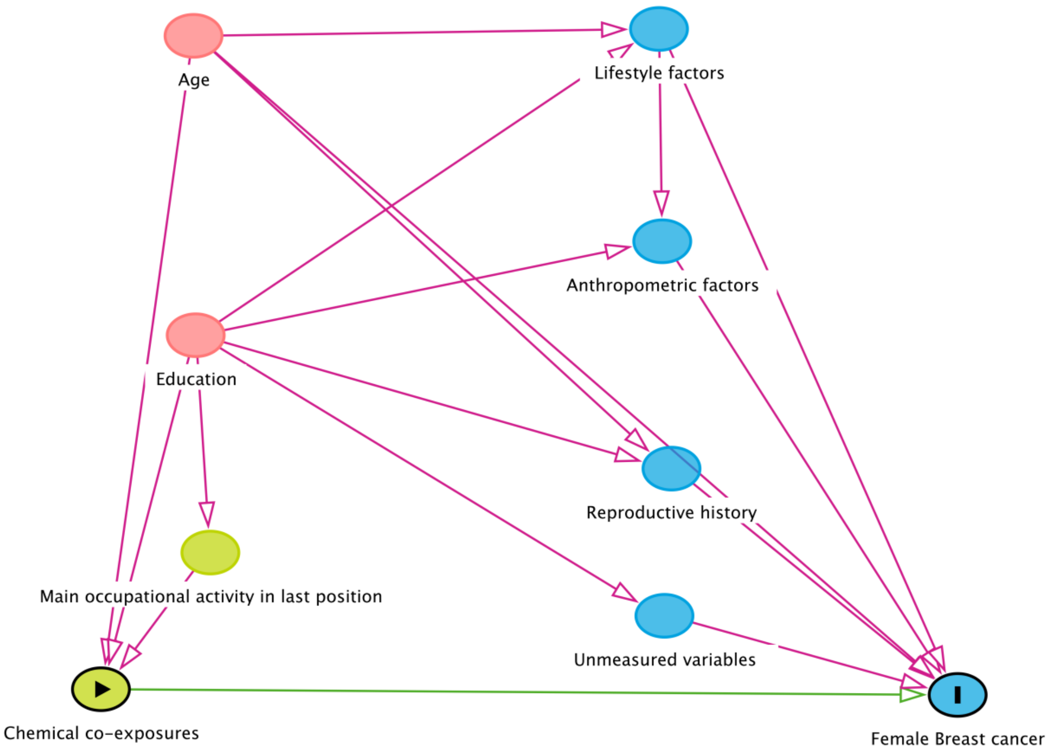

**Chemical co-exposures**:	**Lifestyle factors:**	**Antropometric factors:**	**Reproductive history:**	**Unmeasured variables:**
Chlorinated degreaser	Fibre intake	Height	Age at first child	Exogenous hormones use
Benzene	Multivitamine intake	Weight	Number of children	Breast feeding
	Dietary fat intake		Menopausal status	Family history
	Alcohol intake			
	Smoking			
	Physical activity			

Liu FC, *et al. BMJ Open* 2022; 12:e056396. doi: 10.1136/bmjopen-2021-056396
