# Supplementary material for: Night shift work, chemical coexposures and risk of female breast cancer in the Norwegian Offshore Petroleum Workers (NOPW) cohort: a prospectively recruited case-cohort study
Source: BMJ Open. 2022 Jan 24;12(1):e056396. doi: 10.1136/bmjopen-2021-056396 (PMC8788313; doi:10.1136/bmjopen-2021-056396)
Supplement: Author's manuscript [file bmjopen-2021-056396.draft_revisions.pdf]

BMJ Open is committed to open peer review. As part of this commitment we make the peer review history of every article we publish publicly available.

When an article is published we post the peer reviewers' comments and the authors' responses online. We also post the versions of the paper that were used during peer review. These are the versions that the peer review comments apply to.

The versions of the paper that follow are the versions that were submitted during the peer review process. They are not the versions of record or the final published versions. They should not be cited or distributed as the published version of this manuscript.

BMJ Open is an open access journal and the full, final, typeset and author-corrected version of record of the manuscript is available on our site with no access controls, subscription charges or pay-per-view fees (http://bmjopen.bmj.com).

If you have any questions on BMJ Open's open peer review process please email info.bmjopen@bmj.com

BMJ Open

# Night Shift Work, Chemical Co-exposures and Risk of Female Breast Cancer in the Norwegian Offshore Petroleum Workers (NOPW) Cohort: a prospective case-cohort study


| | |
|---|---|
| Journal: | *BMJ Open* |
| Manuscript ID | bmjopen-2021-056396 |
| Article Type: | Original research |
| Date Submitted by the Author: | 12-Aug-2021 |


| | |
|---|---|
| Complete List of Authors: | Liu, Fei-Chih; Cancer Registry of Norway, Department of Research; University of Oslo Faculty of Medicine, Department of Biostatistics<br>Veierød, Marit ; University of Oslo Faculty of Medicine, Department of Biostatistics<br>Kjærheim, Kristina; Cancer Registry of Norway, Department of Research<br>Robsahm, Trude; Cancer Registry of Norway, Department of Research<br>Ghiasvand, Reza; Cancer Registry of Norway, Department of Research; Oslo University Hospital, Oslo Centre for Biostatistics and Epidemiology<br>Hosgood, Dean; Albert Einstein College of Medicine, Department of Epidemiology and Population Health<br>Samuelsen, Sven Ove; University of Oslo, Department of Mathematics<br>Bråtveit, Magne; Universitetet i Bergen Det medisinsk-odontologiske fakultet, Department of Global Public Health and Primary Care<br>Kirkeleit, Jorun; Universitetet i Bergen Det medisinsk-odontologiske fakultet, Department of Global Health and Primary Care<br>Rothman, Nat; National Cancer Institute, Occupational and Environmental Epidemiology<br>Lan, Qing; National Cancer Institute, Occupational and Environmental Epidemiology<br>Silverman, Debra; National Cancer Institute, Occupational and Environmental Epidemiology<br>Friesen, Melissa C.; National Cancer Institute, Occupational and Environmental Epidemiology<br>Babigumira, Ronnie; Cancer Registry of Norway, Department of Research<br>Shala, Nita; Cancer Registry of Norway, Department of Research; University of Oslo Faculty of Medicine, Department of Biostatistics<br>Grimsrud, Tom; Cancer Registry of Norway, Department of Research<br>Stenehjem, Jo; Cancer Registry of Norway, Department of Research |


| | |
|---|---|
| Keywords: | EPIDEMIOLOGY, Breast tumours < ONCOLOGY, OCCUPATIONAL & INDUSTRIAL MEDICINE |
| | |


SCHOLARONE™ Manuscripts

*I, the Submitting Author has the right to grant and does grant on behalf of all authors of the Work (as defined in the below author licence), an exclusive licence and/or a non-exclusive licence for contributions from authors who are: i) UK Crown employees; ii) where BMJ has agreed a CC-BY licence shall apply, and/or iii) in accordance with the terms applicable for US Federal Government officers or employees acting as part of their official duties; on a worldwide, perpetual, irrevocable, royalty-free basis to BMJ Publishing Group Ltd ("BMJ") its licensees and where the relevant Journal is co-owned by BMJ to the co-owners of the Journal, to publish the Work in this journal and any other BMJ products and to exploit all rights, as set out in our licence.*

*The Submitting Author accepts and understands that any supply made under these terms is made by BMJ to the Submitting Author unless you are acting as an employee on behalf of your employer or a postgraduate student of an affiliated institution which is paying any applicable article publishing charge ("APC") for Open Access articles. Where the Submitting Author wishes to make the Work available on an Open Access basis (and intends to pay the relevant APC), the terms of reuse of such Open Access shall be governed by a Creative Commons licence – details of these licences and which Creative Commons licence will apply to this Work are set out in our licence referred to above.*

*Other than as permitted in any relevant BMJ Author's Self Archiving Policies, I confirm this Work has not been accepted for publication elsewhere, is not being considered for publication elsewhere and does not duplicate material already published. I confirm all authors consent to publication of this Work and authorise the granting of this licence.*

For peer review only

**MAIN DOCUMENT**

**Title:** Night Shift Work, Chemical Co-exposures and Risk of Female Breast Cancer in the Norwegian Offshore Petroleum Workers (NOPW) Cohort: a prospective case-cohort study.

**Authors:** Fei-Chih Liu[1,2], Marit Bragelien Veierød[2], Kristina Kjærheim[1], Trude Eid Robsahm[1], Reza Ghiasvand[1,3], H. Dean Hosgood[4], Sven Ove Samuelsen[5], Magne Bråtveit[6], Jorunn Kirkeleit[6], Nathaniel Rothman[7], Qing Lan[7], Debra T. Silverman[7], Melissa C. Friesen[7], Ronnie Babigumira[1], Nita K. Shala[1,2], Tom K. Grimsrud[1], Jo S. Stenehjem[1].

1) Department of Research, Cancer Registry of Norway, Institute of Population based Cancer Research, Oslo, Norway.
2) Oslo Centre for Biostatistics and Epidemiology, Department of Biostatistics, Institute of Basic Medical Sciences, University of Oslo, Norway.
3) Oslo Centre for Biostatistics and Epidemiology, Oslo University Hospital, Norway.
4) Department of Epidemiology and Population Health, Albert Einstein College of Medicine, Bronx, NY, United States.
5) Department of Mathematics, University of Oslo, Oslo, Norway.
6) Department of Global Public Health and Primary Care, University of Bergen, Bergen, Norway.
7) Occupational and Environmental Epidemiology Branch, Division of Cancer Epidemiology and Genetics, National Cancer Institute, Bethesda, MD, United States

Address correspondence to:
Fei-Chih Liu, MD, MPH
Post: Cancer Registry of Norway, P.O. Box 5313 Majorstuen, NO-0304 Oslo, Norway
E-mail: Fei-Chih.Liu@kreftregisteret.no
Phone: +47 913 54 452

Word-count: 222 (abstract) 3,482(main text)

Number of references: 44

Number of tables and figures: 5

Conflict of interest: None declared.



For peer review only - http://bmjopen.bmj.com/site/about/guidelines.xhtml

## ABSTRACT

**Objectives:** This study examined the association between night shift work and risk of breast cancer, overall and by hormone receptor subtype, among females in the Norwegian Offshore Petroleum Workers (NOPW) cohort. We also examined the association of co-exposure (chlorinated degreasers and benzene) and breast cancer risk, and possible interaction with work schedule.

**Design:** Prospective case-cohort study within the NOPW cohort.

**Setting:** Female offshore petroleum workers active on the Norwegian continental shelf.

**Participants**: 600 female workers (86 cases and 514 non-cases) were included in the study. We excluded workers that died or emigrated before start of follow-up, had missing work history, were diagnosed with breast cancer or other prior malignancy (except non-melanoma skin cancer) before start of follow-up.

**Results:** No overall association was found between breast cancer risk and work schedule (hazard ratio=0.87, 95% confidence interval: 0.52, 1.46 for work schedule involving night shift *vs*. day shift only). There was no significant association between work schedule and risk of any breast cancer subtype. No significant interactions were found between work schedule and chemical co-exposures (breast cancer overall $P_{\text{interaction chlorinated degreasers}}$=0.725 and $P_{\text{interaction benzene}}$=0.175).

**Conclusions:** Our results did not provide supporting evidence that work schedule involving night shift affects breast cancer risk in female offshore petroleum workers, but should be considered cautiously due to few cases. Further studies with larger sample sizes are warranted.




For peer review only - http://bmjopen.bmj.com/site/about/guidelines.xhtml

**Strengths and limitations of this study**

- Workers in the NOPW cohort have extreme work schedules that provide interesting data to study the association between night shift work and breast cancer.
- The Norwegian cancer and population registries ensured high quality cancer data and complete control of loss to follow-up.
- Job-exposure matrices of chemical exposure in the offshore work environment were developed specifically for cancer studies in the NOPW cohort.
- The females in the NOPW cohort were young at baseline and had relatively few breast cancer cases yielding limited statistical power.
- We had no information on exposures during follow-up, and lacked information on potential confounders such as family history of breast cancer, exogenous hormonal use and chronotype.

For peer review only



For peer review only - http://bmjopen.bmj.com/site/about/guidelines.xhtml

## INTRODUCTION

Breast cancer is the most frequent cancer among females and the incidence is higher in industrialised compared to developing countries (1). The increase seen in modern industrialised societies has mainly been attributed to reproduction and lifestyle factors (2, 3). However, the use of artificial light during work at night, leading to disruption of the internal circadian clock and suppressed melatonin production, may accelerate the transformation of malignant mammary cells and promote the risk of breast cancer (4, 5).

Norwegian offshore workers employed at oil and gas installations have an extreme touring work pattern (6). A standard two-week tour on Norwegian installations is minimum 12 hours of work seven days per week. The work schedule is either 14 day shifts, 14 night shifts or 14 rollover shifts (starting with 7 days followed by 7 nights or *vice versa*) (6). During a night shift, offshore workers are constantly exposed to artificial light (6). These extreme working hours meet the definition of night shift work (≥7 hours of work including the period from midnight to 05:00 hours) (7, 8).

The relationship between night shift work and breast cancer is still unclear. Several studies have reported a positive association between night shift work and breast cancer (9-12), while others did not (13-16). A challenge has been the lack of high-quality exposure data on light during night time and detailed data on night shift work schedules (4). A recent pooled analysis of five population-based case-control studies with complete work history reported an increased risk of breast cancer in pre-menopausal females who had high intensity and long duration of night shift work (17). Further, breast cancer risk in relation to night shift work may be elevated among pre-menopausal females with a positive tumour status to oestrogen receptor (ER), progesterone receptor (PR), or human epidermal growth factor receptor 2 (HER2), but the evidence is inconclusive (18, 19).



For peer review only - http://bmjopen.bmj.com/site/about/guidelines.xhtml

Based on the limited evidence for the carcinogenicity of night shift work, the International Agency for Research on Cancer (IARC) recently reconfirmed night shift work as a probable carcinogen to humans (Group 2A) (20), asking for improved exposure assessment in future studies to reduce heterogeneity between studies and allow for better comparisons. IARC also encouraged better reporting of potential co-exposures to occupational carcinogens. On offshore petroleum installations, some female workers may be exposed to a number of carcinogens, but in particular benzene during drilling, production and maintenance (21, 22), and chlorinated degreasers during cleaning of metal parts (trichloroethylene) or dry-cleaning of work clothes (tetrachloroethylene) (21). Cohort and population-based studies have linked Benzene and tetrachloroethylene to increased breast cancer risk (23, 24), and recently, night shift work has received increased attention as a possible effect modifier of the association between chemical exposure and cancer by desynchronising detoxification mechanisms (25) (26). To our knowledge, chemical exposure and night shift work have not been examined simultaneously in relation to breast cancer risk.

The Norwegian Offshore Petroleum Workers (NOPW) cohort holds information on work schedule for each individual employed 1965–1998 and job exposure matrices (JEMs) capturing chemical exposures in the offshore work environment (27). The aim of the present study was to prospectively examine the association between shift work and breast cancer risk, overall and by receptor status subtypes, among females in the NOPW cohort. We also examined the association of chemical exposures (chlorinated degreasers and benzene) and breast cancer risk, and possible multiplicative interactions with work schedule.



For peer review only - http://bmjopen.bmj.com/site/about/guidelines.xhtml

# MATERIALS AND METHODS

## Patient and public involvement

This study was conducted without patient involvement.

## Study population and study design

The NOPW cohort was established in 1998 by the Cancer Registry of Norway (CRN). In 1998, questionnaires were sent to current or former male and female offshore petroleum workers with a minimum of 20 days' work on the Norwegian continental shelf between 1965 and 1998 (27). In total, 27,917 workers were included (estimated response rate 69%) in the NOPW cohort (28). Details on the cohort and its establishment have been published elsewhere (27, 28). All workers filled in an informed consent for participation in the study. Necessary legal and ethical approvals have been obtained from the Norwegian Data Inspectorate, the Regional Committee for Medical Research Ethics (2018/1162), and the Norwegian Directorate of Health.

We used a stratified case-cohort design (29) as complete information on work history had to be extracted manually from the questionnaires for breast cancer cases and a random subsample of the cohort (hereafter the 'subcohort'). The case-cohort design allows prospective models such as Cox-regression, and the subcohort may be used as controls for different types of cancer cases, as it is not matched to specific cases.

## Identification of cancer cases

The NOPW cohort was linked to the CRN and the National Population Register for information on cancer, death and emigration, by using the unique personal identification numbers assigned to all Norwegian citizens (30). Follow-up started 1 July 1999 and ended 31 December 2017. Reporting of incident cancers to the CRN is mandatory in Norway and the



For peer review only - http://bmjopen.bmj.com/site/about/guidelines.xhtml

degree of completeness and validity is high, with morphology verified for 99.3% of the breast cancers (30). Cancer cases were defined according to the International Classification of Diseases, Tenth Revision (ICD-10), C50 for breast cancer.

Immunohistochemistry and pathology reports submitted routinely to the CRN were used to assess breast cancer receptor status according to the St. Gallen Consensus 2011 (31). Tumours which were HER2-negative and ER-positive and/or PR-positive were classified as luminal A; tumours which were HER2-positive and ER-positive and/or PR-positive were regarded as luminal B; and tumours which were HER2-negative and ER-negative and PR-negative as triple negative subtype.

**Study sample**

A total of 25,347 males were excluded from the full cohort, and breast cancer cases and the subcohort were identified among the remaining 2,570 female offshore workers (Figure 1). Among the cases, we applied the exclusion criteria (i) death or emigration before start of follow-up (n=0) (ii) missing work history (n=10), (iii) breast cancer before start of follow-up (n=7), and (iv) other prior malignancy except non-melanoma skin cancer before start of follow-up (mainly squamous cell carcinoma as the CRN does not routinely record information on basal cell carcinoma of the skin [n=0]), resulting in 86 breast cancer cases in the analyses (Figure 1).

The initial subcohort (n=557) was sampled from the female cohort (n=2,570) and was established in two steps to secure at least 5 non-cases per case. First, we oversampled (i.e. selected all) the female cohort members born 1920–1939 and 1975–1979 (n=102) to secure comparable non-cases for the oldest and youngest cases. Second, for those born 1940–1974, we drew at random within strata of 5-year birth cohorts 455 female workers, who were frequency matched to the birth year distribution of all potentially occupational cancers



For peer review only - http://bmjopen.bmj.com/site/about/guidelines.xhtml

estimated for 2017 (n=91). We excluded 43 females that died or emigrated before start of follow-up (n=1), had missing work history (n=3), were diagnosed with breast cancer (n=5) or other prior malignancy (except non-melanoma skin cancer [n=12]) before start of follow-up, or were diagnosed with breast cancer during follow-up (cases, n=22). Thus, for our analyses, 514 females remained in the subcohort (Figure 1).

**Exposure assessment**

*Work schedule*

We handled work history data of 514 female subcohort members and 86 cases who reported up to eight employments each. The process of harmonising overlapping employment records were handled by collapsing jobs within the same category and by splitting jobs of different categories into proportionally equal parts, according to a method described previously (32).

The self-reported work schedule for each job was recorded as either day, night, or rollover shift for two-week tours. Day shift is equivalent to 14 consecutive days of day-time work usually between 07:00 and 19:00 hours. Night shift is equivalent to 14 consecutive days of night-time work usually between 19:00 and 07:00 hours. Rollover shift is equivalent to 7 consecutive days of day-time work (same working hours as for day shift) then alternate to 7 consecutive days of night-time work (same working hours as for night shift), or *vice versa*.

Some worked only day, but none worked only night or only rollover. Therefore, the variable work schedule was categorised as 1) day work only, 2) mixed I (some night/rollover + mainly day), 3) mixed II (mainly night/rollover + some day), and 4) missing. We then collapsed categories 2 (mixed I) and 3 (mixed II) into exposed (night/rollover shift) to represent workers that had ever worked some degree of night-time. For each individual, the duration of night/rollover shift was calculated by (i) cumulating the total number of years with



For peer review only - http://bmjopen.bmj.com/site/about/guidelines.xhtml

night shifts, (ii) cumulating the total number of years with rollover shifts, (iii) summarising the number of years worked with night and rollover shifts into a combined continuous variable, and (iv) categorising this variable according to median (unexposed, <1-6, >6) or quartile (unexposed, <1–2, >2–6, >6–10, >10) years of night/rollover shift. Total employment duration was categorised according to quartiles (0–1.9, 2–5.9, 6–10.9, 11–24), expressed in years.

*Chemical co-exposures and main occupational activity in last position*

A research group at the University of Bergen has developed expert-based job-exposure matrices (JEMs) for the NOPW cohort to identify and describe the degree of exposure to agents, mixtures or exposure situations with known or suspected carcinogenic potential among offshore workers on the Norwegian continental shelf employed in 1970–2005 (22, 33). The JEMs and their development have been described in detail elsewhere (22, 34). In brief, due to lack of exposure measurements, the assessment of the JEM for chlorinated degreasers was probability-oriented and based on summary documents (*e.g.* from company visits/interviews, risk assessment reports, sampling reports, product data sheets)on the exposure to tri- and tetrachloroethylene. Eight experts individually categorised the likelihood (unlikely, possible, probable) of chlorinated degreaser exposure (*i.e.* products containing tri- and tetrachloroethylene) for 27 job categories (33). Importantly, the exposure assessment of chlorinated degreasers was evaluated as “possible” for catering workers, meaning that some workers may have been exposed while the probability of exposure was low. For the benzene JEM, measurement data were used to construct a time-line of exposure and to rate exposure intensity of tasks specific to each job category. Then, task duration and frequency were combined to create a semi-quantitative benzene exposure burden score specific to job

For peer review only



For peer review only - http://bmjopen.bmj.com/site/about/guidelines.xhtml

category and time period (1965–1969, 1970–1979, 1980–1989, 1990–1998) (34). For each of the two agents, we generated a dichotomized variable indicating never or ever exposure.

Main occupational activity in each worker's last position was recorded as production and process, drilling and well maintenance, maintenance/inspection/deck construction, catering/office/administration, and miscellaneous.

*Covariates*

Age at first child and number of children were recorded in the questionnaire, categorized as 0, 1, 2, ≥3 children. Following the convention from the Million Women Study (35), females aged ≥53 years at baseline in 1998 were assumed postmenopausal. Education was recorded as compulsory, vocational, folk high school, upper secondary (the two latter collapsed into upper secondary), and university/college.

**Data analysis**

Cox regression, adapted to a stratified case-cohort design (29), was used to estimate hazard ratios (HRs) of breast cancer and 95% confidence intervals (CIs) associated with work schedule, chlorinated degreasers and benzene. Cases were assigned a weight of 1 and subcohort non-cases were given weights according to the inverse sampling fraction in their corresponding 5-year birth cohort stratum. In the analyses, age was used as the time-scale with subjects entering start of follow-up on 30 June 1999. Subjects were censored at the date of any cancer diagnosis (except non-melanoma skin cancer), emigration, death, or end of study, whichever came first. The 22 cases identified as members of the randomly drawn subcohort were analysed as cases only (weight=1) (29). Robust variance was used to compute standard errors of the HRs. The proportional hazards assumption was evaluated by Schoenfeld residuals.



For peer review only

For peer review only - http://bmjopen.bmj.com/site/about/guidelines.xhtml

Based on the literature and the available variables in NOPW, a directed acyclic graph (DAG) was drawn to help model the relationship between work schedule and breast cancer risk (Figure S1, Supplemental Material). Based on the DAG, we adjusted for age at first child, number of children, menopausal status at baseline and education in addition to attained age (timescale). Likewise, based on a DAG (Supplemental Material, Figure S2) we adjusted for age and education in the analyses of chlorinated degreasers and benzene in relation to breast cancer risk. We tested for linear trend by modelling ordinal variables as continuous variables. Tests of interaction between work schedule and chemical co-exposures, and between work schedule and menopausal status were conducted by including product terms of dichotomous variables in the models.

We had up to 39% missing in the covariates of the multivariable model. We used multiple imputation with chained equations, assuming missing at random (36), to impute 45 datasets. In each data set, we conducted analyses using the models described above, and estimates were combined using Rubin's rule (37). We report results with and without imputed data. We have extended the description of the imputations in the supplements where we also tried an alternative imputation model (Supplemental Material, Methods extension, Table S4).

Tests for significance were two-sided, and P-values <0.05 were considered to represent statistical significance. Data analyses were performed using Stata version 16.1 (StataCorp, College Station, TX, USA).

## RESULTS

Table 1 shows baseline characteristics of the 86 cases and 514 non-cases in the study sample. Mean age at baseline was 45 years for cases and 42 years for non-cases. Among the cases and non-cases, mean employment duration was 8.4 and 6.9 years, the most frequent main occupational activity in last position was catering/office/administration (73% *vs.* 71%,



For peer review only - http://bmjopen.bmj.com/site/about/guidelines.xhtml

For peer review only

respectively), most had day work only (55% *vs.* 53%), and 37% of the cases *vs.* 42% of the non-cases were exposed to night/rollover shift. Twenty-seven percent of the cases had >6 years of night/rollover shift compared with 18% of non-cases. Up to 68% of the cases and 77% of the non-cases may have been exposed to chlorinated degreasers. For benzene, 15% of the cases and 18% of the non-cases were exposed. Mean age at breast cancer diagnosis was 56 years, whereof 78% were ER-positive and 69% PR-positive, while 70% were HER2-negative (Supplemental Material, Table S1). Fifty-four percent of the tumours were classified as luminal A, 12% as luminal B, and 13% as triple negative subtype.

No interaction was found between work schedule and menopausal status when examining risk of overall or any breast cancer subtypes. The P-values of the interaction terms in the models were between 0.279 and 0.639, we therefore do not present results stratified by menopausal status. Results from complete-case and multiple imputation analyses were largely similar across all analyses (Tables 2-4), and we refer to the multiple imputation results in the following.

No association was found between breast cancer risk and total employment duration ($P_{trend}$=0.733) (Table 2). The HR of breast cancer in relation to work schedule involving night/rollover shift was 0.87 (95% CI: 0.52, 1.46), and no significant association was found with duration of night/rollover shift ($P_{trend}$=0.748). Sensitivity analyses of duration of night/rollover shift by quartile among the exposed showed similar result ($P_{trend}$=0.944) (Supplemental Material, Table S2).

There was no significant association between main occupational activity in last position and breast cancer risk (Table 3). No significant associations with breast cancer risk were found for chlorinated degreaser or benzene exposure (HRs (95% CIs) 0.89 (0.52, 1.52) and 0.90 (0.46, 1.79), respectively, for ever *vs*. never, Table 3). No significant interaction was



For peer review only - http://bmjopen.bmj.com/site/about/guidelines.xhtml

For peer review only

found between work schedule and chemical co-exposures ($P_{interaction\ chlorinated\ degreasers}$=0.725; $P_{interaction\ benzene}$=0.175) (Supplemental Material, Table S3).

There was no significant association between work schedule and risk of any breast cancer subtypes. For the breast cancer subtypes that did not express sex hormone receptors we found non-significantly elevated risks for work schedule involving night/rollover shift compared to day work only (HR=1.53, 95% CI: 0.50, 4.72 for ER-negative tumours; HR=1.69, 95% CI: 0.58, 4.87 for PR-negative tumours; HR=2.23, 95% CI: 0.58, 8.55 for triple negative tumours) (Table 4).

## DISCUSSION

In this prospective study of female offshore workers, we observed no association between overall breast cancer risk and night/rollover shift, duration of such work, or total employment duration. Some indication of increased risks was observed for ER-negative and PR-negative breast cancer subtypes, but these findings were not statistically significant and were based on few observations. We did not find evidence for interaction between work schedule and chemical co-exposures in relation to risk of breast cancer.

The literature is inconclusive on the association between night shift work and breast cancer risk. A report from the Nurses Health Study (9) found an elevated risk among nurses who had long (≥20 years) cumulative night shift work and started their career early, which is also in accordance with other studies (10, 11, 17). One explanation for this positive associations between short-term night shift work and breast cancer risk may be that breast tissue is particularly susceptible to circadian disruption at a younger age (9). However, in line with our main findings, recent studies did not find an association between night shift work and breast cancer risk (13-15). Importantly, the most recent meta-analysis, stratifying on short- and long-term night shift work, found an increased breast cancer risk related to short-term, but not long-term night shift work (38). Possible reasons that may explain our negative



For peer review only - http://bmjopen.bmj.com/site/about/guidelines.xhtml

results are that, despite the extreme and unique work pattern, offshore workers have a four weeks off-duty period following each standard two-week tour (6). During the off-duty period, it is possible that workers could rest and readjust their circadian rhythm, and hence reduce the effect of night shift work on breast cancer risk. Further, the continuous shift work pattern (7–14 consecutive nights) of offshore workers might facilitate some adaptation and thereby represent a lower degree of circadian disruption, which in turn might reduce the risk of breast cancer (5).

To our knowledge, this is the first study of night shift work and co-exposure to chlorinated degreaser and benzene in relation to breast cancer risk. The hypothesis that night shift work may potentiate chemical exposure by desynchronising detoxification mechanisms (26) was not confirmed, as we did not find any statistically significant interactions, although it may be ascribed to lack of power and misclassification of exposure.

Some studies have investigated the relationship between night shift work and breast cancer subtypes. Night shift work has been associated with sex hormone receptor-positive (ER-positive or PR-positive) tumours (14, 17, 18, 39), which is in line with experimental evidence that melatonin suppression enhances proliferation of ER-positive cell lines (40). Zhu et al. (41) suggested that melatonin disturbance could instigate two independent pathways that lead to both ER-positive and ER-negative tumours. This would be in line with our findings of increased risk of ER-negative tumours among night-shift workers, which are consistent with the study by Rabstein et al. (19).

Our prospective analysis has several strengths. First, linkage to nationwide cancer and population registries ensured high quality cancer data and complete follow-up. Second, we had access to independent chemical exposure estimates developed by industrial hygiene experts specifically for cancer studies in our cohort. Third, offshore workers have extreme work schedules and the NOPW cohort therefore provides particularly interesting data to study



For peer review only - http://bmjopen.bmj.com/site/about/guidelines.xhtml

the association with breast cancer. Although, self-reported work history (*i.e.* exposure) was collected prior to the breast cancer diagnoses, and has been shown to be robust to recall errors (42), we cannot rule out some differential misclassification as a result of collapsing exposure categories, possibly yielding biased HRs both towards and away from the null (43, 44). The females in the NOPW cohort were quite young at baseline (mean age 42), which may explain the relatively few cases. Also, the low case numbers in some exposure categories reduced the statistical power of detecting significant deviations from the null hypothesis. Comparing our results with the pooled-analysis of Cordina-Duverger *et al.* (17), which had larger sample size and reported a positive association between night shift work and breast cancer (all receptor subtypes combined), the two studies do not contradict each other (HR=0.87, 95% CI: 0.52, 1.46 *vs.* OR=1.12, 95% CI: 1.00, 1.25). Our study had relatively few cases resulting in low power and wide confidence intervals. The limited power may leave real interactions undetected. In an attempt to reduce the impact of limited power, we tested interactions with binary variables (ever/never exposure). The probability-oriented JEM for chlorinated degreasers evaluated catering workers as 'possibly exposed' and assigned the same exposure level despite the fact that exposure may vary by job tasks. Hence, the actual fraction of workers exposed to chlorinated degreasers in this job-category is uncertain. We also lacked information on some potential confounders (*e.g.* chronotype, exogenous hormone use, family history and breast feeding history), and we had no information on exposures during follow-up.

In conclusion, our findings in this prospective cohort do not provide supporting evidence that night/rollover shift, exposure to chlorinated degreasers or to benzene are associated with breast cancer risk in female offshore petroleum workers. However, case numbers were low for analyses with combined exposures and for analyses of breast cancer by receptor subtype. Therefore, further studies with larger sample sizes are warranted.



For peer review only - http://bmjopen.bmj.com/site/about/guidelines.xhtml

**FUNDING**

This work was supported by the Research Council of Norway grant number 280537.

**CONFLICTS OF INTEREST**

None declared. JSS and TKG have received a grant from the Research Council of Norway (governmental agency) that awarded an industry-collaborative grant to the Cancer Registry of Norway (governmental agency) in 2019 to establish a cohort of offshore petroleum workers. A condition pertaining to industry-collaborative grants is that 20% (175,000 USD) of the grant was provided by the petroleum industry and 80% (700,000 USD) by the Research Council itself with the intention of joining forces for the common interest of occupational health among petroleum workers. The application process was governed by the Research Council without any involvement from the industry. The grant does not cover the PI's or any of the collaborators salary.

**AUTHOR CONTRIBUTIONS**

JSS and TKG conceived the study. FCL, MBV, KK, TER, RG, HDH, SOS, MB, JK, NR, QL, DTS, MCF, RB, NKS, TKG and JSS contributed to the project design. FCL performed the data analyses. FCL, RB, and JSS performed the data management. KK contributed with their expertise on breast cancer epidemiology. MB and JK contributed to chemical exposure assessment. FCL drafted the manuscript and all authors reviewed and revised it critically for important intellectual content and approved the final version for submission. FCL and JSS are the guarantors.

For peer review only



For peer review only - http://bmjopen.bmj.com/site/about/guidelines.xhtml

**ETHICS APPROVAL**

This study was performed at the CRN after legal and ethical approvals were obtained from the Norwegian Data Inspectorate, the Regional Committee for Medical Research Ethics (2018/1162), and the Norwegian Directorate of Health.

**DATA SHARING STATEMENT**

The data that support the findings of this study are available from the CRN (cohort data and cancer data) and the National Population Register (death and emigration data) but restrictions apply to the availability of these data, which were used under license for the current study, and so are not publicly available. Requests for data sharing/case pooling for projects with necessary approvals and legal basis according to the EU General Data Protection Regulation (GDPR) may be directed to principal investigator Dr Tom K Grimsrud; email: tom.k.grimsrud@kreftregisteret.no

For peer review only



For peer review only - http://bmjopen.bmj.com/site/about/guidelines.xhtml

## References

1. Bray F, Ferlay J, Soerjomataram I, Siegel RL, Torre LA, Jemal A. Global Cancer Statistics 2018: Globocan Estimates of Incidence and Mortality Worldwide for 36 Cancers in 185 Countries. CA: a cancer journal for clinicians. 2018;68(6):394-424.
2. Bray F, McCarron P, Parkin DM. The Changing Global Patterns of Female Breast Cancer Incidence and Mortality. Breast Cancer Res. 2004;6(6):229.
3. Francies FZ, Hull R, Khanyile R, Dlamini Z. Breast Cancer in Low-Middle Income Countries: Abnormality in Splicing and Lack of Targeted Treatment Options. Am J Cancer Res. 2020;10(5):1568-91.
4. Hansen J. Risk of Breast Cancer After Night- and Shift Work: Current Evidence and Ongoing Studies in Denmark. Cancer Causes & Control. 2006;17(4):531-7.
5. Stevens RG, Davis S. The Melatonin Hypothesis: Electric Power and Breast Cancer. Environ Health Persp. 1996;104(Suppl 1):135-40.
6. Parkes K. Working hours in the offshore petroleum industry Current knowledge and research needs regarding extended work periods and shift work offshore. In: Conference On Work Time Arrangements in the Petroleum Industry, 2007: Norway: Petroleum Safety Authority; 2007.
7. ILO. C171 - Night Work Convention No. 171 (C171). [Webpage]. Geneva, Switzerland: International Labour Organization: [cited 2020.29.09]. Available from: https://www.ilo.org/dyn/normlex/en/f?p=NORMLEXPUB:12100:0::NO::P12100_INSTRUMENT_ID:312316
8. ILO. R178 - Night Work Recommendation No.178 (R178). [Webpage]. Geneva, Switzerland: International Labour Organization: [cited 2020.29.09]. Available from: https://www.ilo.org/dyn/normlex/en/f?p=NORMLEXPUB:12100:0::NO::P12100_ILO_CODE:R178
9. Wegrzyn LR, Tamimi RM, Rosner BA, Brown SB, Stevens RG, Eliassen AH, et al. Rotating Night-Shift Work and the Risk of Breast Cancer in the Nurses' Health Studies. Am J Epidemiol. 2017;186(5):532-40.
10. Åkerstedt T, Knutsson A, Narusyte J, Svedberg P, Kecklund G, Alexanderson K. Night Work and Breast Cancer in Women: a Swedish Cohort Study. BMJ Open. 2015;5(4):e008127.
11. Hansen J, Lassen CF. Nested Case–Control Study of Night Shift Work and Breast Cancer Risk Among Women in the Danish Military. Occup Environ Med. 2012;69(8):551-6.
12. Lie JS, Kjuus H, Zienolddiny S, Haugen A, Stevens RG, Kjærheim K. Night Work and Breast Cancer Risk Among Norwegian Nurses: Assessment by Different Exposure Metrics. Am J Epidemiol. 2011;173(11):1272-9.
13. Jones ME, Schoemaker MJ, McFadden EV, Wright LB, Johns LE, Swerdlow AJ. Night Shift Work and Risk of Breast Cancer in Women: The Generations Study Cohort. Br J Cancer. 2019;121(2):172-9.
14. Vistisen HT, Garde AH, Frydenberg M, Christiansen P, Hansen AM, Hansen J, et al. Short-Term Effects of Night Shift Work on Breast Cancer Risk: A Cohort Study of Payroll Data. Scand J Work Environ Health. 2017;43(1):59-67.
15. Travis RC, Balkwill A, Fensom GK, Appleby PN, Reeves GK, Wang XS, et al. Night Shift Work and Breast Cancer Incidence: Three Prospective Studies and Meta-Analysis of Published Studies. J Natl Cancer Inst. 2016;108(12):djw169.
16. Koppes LL, Geuskens GA, Pronk A, Vermeulen RC, de Vroome EM. Night Work and Breast Cancer Risk in a General Population Prospective Cohort Study in the Netherlands. Eur J Epidemiol. 2014;29(8):577-84.
17. Cordina-Duverger E, Menegaux F, Popa A, Rabstein S, Harth V, Pesch B, et al. Night Shift Work and Breast Cancer: A Pooled Analysis of Population-Based Case–Control Studies With Complete Work History. Eur J Epidemiol. 2018;33(4):369-79.
18. Cordina-Duverger E, Koudou Y, Truong T, Arveux P, Kerbrat P, Menegaux F, et al. Night Work and Breast Cancer Risk Defined by Human Epidermal Growth Factor Receptor-2 (HER2) and Hormone Receptor Status: A Population-Based Case-Control Study in France. Chronobiology international. 2016;33(6):783-7.



For peer review only - http://bmjopen.bmj.com/site/about/guidelines.xhtml

19. Rabstein S, Harth V, Pesch B, Pallapies D, Lotz A, Justenhoven C, et al. Night Work and Breast Cancer Estrogen Receptor Status--Results From the German Genica Study. Scand J Work Environ Health. 2013;39(5):448-55.
20. IARC. Night Shift Work. IARC Monogr Identif Carcinog Hazards Hum. 2020;124:1-371.
21. Bratveit M, Kirkeleit J, Hollund BE, Moen BE. Biological monitoring of benzene exposure for process operators during ordinary activity in the upstream petroleum industry. The Annals of occupational hygiene. 2007;51(5):487-94.
22. Steinsvåg K, Bratveit M, Moen BE. Exposure to Carcinogens for Defined Job Categories in Norway's Offshore Petroleum Industry, 1970 to 2005. Occup Environ Med. 2007;64(4):250-8.
23. Garcia E, Hurley S, Nelson DO, Hertz A, Reynolds P. Hazardous Air Pollutants and Breast Cancer Risk in California Teachers: A Cohort Study. Environmental health : a global access science source. 2015;14(1):14.
24. Pedersen JE, Strandberg-Larsen K, Andersson M, Hansen J. Occupational Exposure to Specific Organic Solvents and Risk of Subtypes of Breast Cancer in a Large Population of Danish Women, 1964-2016. Occup Environ Med. 2020;oemed-2020-106865. doi: 10.1136/oemed-2020-106865. Online ahead of print.
25. Masri S, Sassone-Corsi P. The Emerging Link Between Cancer, Metabolism, and Circadian Rhythms. Nat Med 2018;24:1795-803.
26. Carmona-Antoñanzas G, Santi M, Migaud H, Vera LM. Light- and Clock-Control of Genes Involved in Detoxification. Chronobiology international. 2017;34(8):1026-41.
27. Stenehjem JS, Babigumira R, Hosgood HD, Veierød MB, Samuelsen SO, Bråtveit M, et al. Cohort Profile: The Norwegian Offshore Petroleum Workers (NOPW) Cohort. Int J Epidemiol. 2020;dyaa107.
28. Stenehjem JS, Kjaerheim K, Rabanal KS, Grimsrud TK. Cancer Incidence Among 41,000 Offshore Oil Industry Workers. Occup Med (Lond). 2014;64(7):539-45.
29. Borgan O, Langholz B, Samuelsen SO, Goldstein L, Pogoda J. Exposure Stratified Case-Cohort Designs. Lifetime Data Anal. 2000;6(1):39-58.
30. Larsen IK, Smastuen M, Johannesen TB, Langmark F, Parkin DM, Bray F, et al. Data Quality at the Cancer Registry of Norway: An Overview of Comparability, Completeness, Validity and Timeliness. Eur J Cancer. 2009;45(7):1218-31.
31. Goldhirsch A, Wood WC, Coates AS, Gelber RD, Thürlimann B, Senn H.J. Strategies for Subtypes-Dealing With the Diversity of Breast Cancer: Highlights of the St. Gallen International Expert Consensus on the Primary Therapy of Early Breast Cancer 2011. Annals of oncology : official journal of the European Society for Medical Oncology. 2011;22(8):1736-47.
32. Stenehjem JS, Babigumira R, Friesen MC, Grimsrud TK. Harmonizing Work History Data in Epidemiologic Studies With Overlapping Employment Records. American journal of industrial medicine. 2019;62(5):422-9.
33. Steinsvag K, Bratveit M, Moen BE, Kromhout H. Inter-rater agreement in the assessment of exposure to carcinogens in the offshore petroleum industry. Occupational and environmental medicine. 2007;64(9):582-8.
34. Bråtveit M, Kirkeleit J, Hollund BE, Vågnes KS. Development of a Retrospective JEM for Benzene in the Norwegian Oil and Gas Industry. Occup Environ Med. 2011;68(Suppl 1):A26-A.
35. Beral V. Breast Cancer and Hormone-Replacement Therapy in the Million Women Study. Lancet (London, England). 2003;362(9382):419-27.
36. Bartlett JW, Seaman SR, White IR, Carpenter JR. Multiple Imputation of Covariates by Fully Conditional Specification: Accommodating the Substantive Model. Stat Methods Med Res. 2015;24(4):462-87.
37. Rubin DB. Multiple Imputation for Nonresponse in Surveys. Hoboken, New Jersey: John Wiley & Sons; 2004. 131 p.
38. Manouchehri E, Taghipour A, Ghavami V, Ebadi A, Homaei F, Latifnejad Roudsari R. Night-Shift Work Duration and Breast Cancer Risk: An Updated Systematic Review and Meta-Analysis. BMC women's health. 2021;21(1):89.



For peer review only - http://bmjopen.bmj.com/site/about/guidelines.xhtml

39. Lie JS, Kjuus H, Zienolddiny S, Haugen A, Kjærheim K. Breast Cancer Among Nurses: Is the Intensity of Night Work Related to Hormone Receptor Status? American journal of epidemiology. 2013;178(1):110-7.
40. Girgert R, Bartsch C, Hill SM, Kreienberg R, Hanf V. Tracking the Elusive Antiestrogenic Effect of Melatonin: A New Methodological Approach. Neuro endocrinology letters. 2003;24(6):440-4.
41. Zhu K, Bernard LJ, Levine RS, Williams SM. Estrogen Receptor Status of Breast Cancer: A Marker of Different Stages of Tumor or Different Entities of the Disease? Med Hypotheses. 1997;49(1):69-75.
42. Wärneryd B, Thorslund M, Ostlin P. The Quality of Retrospective Questions About Occupational History-a Comparison Between Survey and Census Data. Scandinavian journal of social medicine. 1991;19(1):7-13.
43. Wacholder S, Dosemeci M, Lubin JH. Blind Assignment of Exposure Does Not Always Prevent Differential Misclassification. American journal of epidemiology. 1991;134(4):433-7.
44. Jurek AM, Greenland S, Maldonado G, Church TR. Proper Interpretation of Non-Differential Misclassification Effects: Expectations vs Observations. Int J Epidemiol. 2005;34(3):680-7.

For peer review only



For peer review only - http://bmjopen.bmj.com/site/about/guidelines.xhtml

# TABLES

**Table 1.** Baseline Characteristics of Females in the Norwegian Offshore Petroleum Workers (NOPW) Cohort.

| Variables | Cases (n=86) | Non-cases (n= 514) |
|---|---|---|
| Age at baseline in 1998 (years), mean (range) | 45 (25-65) | 42 (19-74) |
| | | |
| **Anthropometric factors** | | |
| Height (cm), mean (range)[a] | 167 (155-184) | 167 (151-187) |
| Weight (kg), mean (range)[a] | 66 (49-95) | 66 (43-163) |
| BMI ($kg/m^2$), n (%) | | |
| 12-18.4 | 1 (1) | 13 (2) |
| 18.5-24.9 | 63 (73) | 343 (67) |
| 25.0-29.9 | 15 (18) | 121 (24) |
| ≥30.0 | 5 (6) | 29 (6) |
| Missing | 2 (2) | 8 (1) |
| | | |
| **Reproductive history** | | |
| Number of children, n (%) | | |
| 0 | 29 (34) | 151 (29) |
| 1 | 24 (28) | 85 (16) |
| 2 | 13 (15) | 147 (29) |
| ≥3 | 20 (23) | 123 (24) |
| Missing | 0 (0) | 8 (2) |
| Age at first child, mean (range)[a] | 26 (16-38) | 24 (16-41) |
| Postmenopausal at baseline, n (%)[b] | 18 (21) | 111 (22) |
| | | |
| **Social-demographic history** | | |
| Education, n (%) | | |
| Compulsory | 18 (21) | 106 (21) |
| Vocational | 17 (20) | 118 (23) |
| Upper secondary | 23 (27) | 148 (29) |
| University/college | 24 (27) | 138 (26) |
| Missing | 4 (5) | 4 (1) |
| | | |
| **Work history** | | |
| Main occupational activity in last position, n (%) | | |
| Production and process | 2 (2) | 23 (4) |
| Drilling and well maintenance | 3 (3) | 22 (4) |
| Maintenance/inspection/deck construction | 9 (11) | 59 (12) |
| Catering/office/administration | 63 (73) | 364 (71) |
| Miscellaneous | 9 (11) | 39 (8) |
| Missing | 0 (0) | 7 (1) |
| Total employment duration (years), mean (range) | 8.4 (0.3-20) | 6.9 (0.1-24) |
| Total employment duration quartile, n (%) | | |
| Quartile 1 (0-1.9 years) | 13 (15) | 124 (24) |
| Quartile 2 (2-5.9 years) | 23 (27) | 146 (28) |
| Quartile 3 (6-10.9 years) | 26 (30) | 121 (24) |
| Quartile 4 (11-24 years) | 24 (28) | 123 (24) |
| Work schedule, n (%) | | |
| Day work only | 47 (55) | 275 (53) |
| Mixed I (some night/rollover + mainly day) | 1 (1) | 18 (4) |
| Mixed II (mainly night/rollover + some day) | 31 (36) | 196 (38) |
| Missing | 7 (8) | 25 (5) |
| Duration by work schedule [a,c] | | |
| Day work (years), mean (range) | 6.4 (0.3-20) | 5.7 (0.1-21) |
| Night work (years), mean (range) | 3.6 (2.0-5.3) | 2.0 (0.1-11) |
| Rollover shift (years), mean (range) | 9.3 (1.0-18) | 6.8 (0.1-22) |
| Duration of night/rollover shift, n (%) | | |
| Unexposed (0 years, day work only) | 47 (55) | 275 (53) |
| ≤Median night/rollover (<1-6 years) [d] | 9 (10) | 121 (24) |
| >Median night/rollover (>6 years) [d] | 23 (27) | 93 (18) |
| Missing | 7 (8) | 25 (5) |
| | | |
| **Chemical co-exposure** | | |
| Chlorinated degreasers duration, n (%) [e] | | |
| Unexposed (0 years) | 28 (32) | 121 (23) |



For peer review only - http://bmjopen.bmj.com/site/about/guidelines.xhtml

| | | |
|---|---|---|
| ≤Median (<1–5 years) | 22 (26) | 214 (42) |
| >Median (6–23 years) | 36 (42) | 179 (35) |
| Benzene duration, n (%) | | |
| Unexposed (0 years) | 73 (85) | 424 (82) |
| Exposed (<1–23 years) | 13 (15) | 90 (18) |
| [a] Missing in continuous variables: Height (n=8); Weight (n=9); Age at first child (n=193); Day work duration (n=32); Night work duration (n=32); Rollover shift duration (n=32); Night + Rollover shift duration (n=32) | | |
| [b] Postmenopause assumed at ≥ 53 years of age | | |
| [c] Workers with complete missing information on day, night and rollover (n=32) were excluded when calculating the mean | | |
| [d] Workers with day work mainly but also worked night/rollover shift were included in this category | | |
| [e] Importantly, the exposure assessment of chlorinated degreasers was probability-based by experts and evaluated as "possible" for catering workers. | | |

For peer review only

**Table 2**. Hazard Ratios of Female Breast Cancer According to Work Schedule in the Norwegian Offshore Petroleum Workers (NOPW) Cohort

| | Complete case analysis (n=367) | | | Multiple imputation (n=600; 86 cases) |
|---|---|---|---|---|
| **Work schedule variable** | **No. of participants** | **No. of cases** | **HR (95% CI)** | **HR (95% CI)** |
| Total employment duration [a,b] | | | | |
| Quartile 1 (0-1.9 years) | 79 | 9 | 1.00 (reference) | 1.00 (reference) |
| Quartile 2 (2-5.9 years) | 99 | 16 | 1.59 (0.58, 4.35) | 1.50 (0.69, 3.27) |
| Quartile 3 (6-10.9 years) | 90 | 8 | 0.68 (0.24, 1.94) | 1.64 (0.78, 3.50) |
| Quartile 4 (11-24 years) | 99 | 16 | 1.21 (0.46, 3.13) | 1.19 (0.54, 2.61) |
| P-trend [c] | | | 0.864 | 0.733 |
| Work schedule involving night/rollover shift [a,b] | | | | |
| Unexposed (day work only) | 217 | 29 | 1.00 (reference) | 1.00 (reference) |
| Exposed (night/rollover shift) | 150 | 20 | 1.06 (0.57, 1.97) | 0.87 (0.52, 1.46) |
| | | | | |
| Duration of night/rollover shift [a,b] | | | | |
| Unexposed (0 years, day work only) | 217 | 29 | 1.00 (reference) | 1.00 (reference) |
| ≤Median night/rollover (<1-6 years) | 68 | 6 | 0.72 (0.28, 1.86) | 0.56 (0.26, 1.22) |
| >Median night/rollover (>6 years) | 82 | 14 | 1.34 (0.67, 2.72) | 1.21 (0.67, 2.18) |
| P-trend [c] | | | 0.523 | 0.748 |

Abbreviations: No=number, HR=hazard ratio, CI=confidence interval

[a] Adjusted for age, age at first child, number of children, menopause status, and education

[b] complete work history i.e. up to 8 employments as an offshore worker

[c] Modelled as a continuous variable to test for linear trend

For peer review only



For peer review only - http://bmjopen.bmj.com/site/about/guidelines.xhtml

| **Table 3**. Hazard Ratios of Female Breast Cancer According to Chemical Co-Exposure in the Norwegian Offshore Petroleum Workers (NOPW) Cohort | | | | |
|---|---|---|---|---|
| | **Complete case analysis (n=367)** | | | **Multiple imputation (n=600; 86 cases)** |
| **Chemical exposure variable** | **No. of participants** | **No. of cases** | **HR (95% CI)** | **HR (95% CI)** |
| Main occupational activity in last position [a] | | | | |
| Production/drilling/maintenance | 58 | 8 | 1.17 (0.47, 2.88) | 0.92 (0.48, 1.79) |
| Catering/office/administration | 280 | 35 | 1.00 (reference) | 1.00 (reference) |
| Miscellaneous | 29 | 6 | 1.70 (0.63, 4.53) | 1.19 (0.54, 2.63) |
| | | | | |
| Chlorinated degreasers [a,b] | | | | |
| Never | 95 | 17 | 1.00 (reference) | 1.00 (reference) |
| Ever | 272 | 32 | 0.72 (0.35, 1.48) | 0.89 (0.52, 1.52) |
| | | | | |
| Benzene [a] | | | | |
| Never | 309 | 40 | 1.00 (reference) | 1.00 (reference) |
| Ever | 58 | 9 | 1.54 (0.65, 3.64) | 0.90 (0.46, 1.79) |

Abbreviations: No=number, HR=hazard ratio, CI=confidence interval

[a] Adjusted for age and education

[b] Importantly, the exposure assessment of chlorinated degreasers was probability-based by experts and evaluated as “possible” for catering workers.

For peer review only

**Table 4** Hazard Ratios of Female Breast Cancer by Receptor Status Subtypes in Relation to Work Schedule in the Norwegian Offshore Petroleum Workers (NOPW) Cohort

| | | Complete case analysis (n=367) | | | Multiple imputation (n=600; 86 cases) |
|---|---|---|---|---|---|
| **Breast cancer subtype** | **Work schedule variable** | **No. of participants** | **No. of cases** | **HR (95% CI)** | **HR (95% CI)** |
| ER-positive | Work schedule involving night/rollover shift [a] | | | | |
| | Unexposed (day work only) | 217 | 23 | 1.00 (reference) | 1.00 (reference) |
| | Exposed (night/rollover shift) | 150 | 16 | 1.00 (0.50, 2.00) | 0.75 (0.41, 1.36) |
| ER-negative | Work schedule involving night/rollover shift [a] | | | | |
| | Unexposed (day work only) | 217 | 5 | 1.00 (reference) | 1.00 (reference) |
| | Exposed (night/rollover shift) | 150 | 2 | 0.78 (0.14, 4.46) | 1.53 (0.50, 4.72) |
| PR-positive | Work schedule involving night/rollover shift [a] | | | | |
| | Unexposed (day work only) | 217 | 25 | 1.00 (reference) | 1.00 (reference) |
| | Exposed (night/rollover shift) | 150 | 13 | 0.80 (0.39, 1.68) | 0.67 (0.36, 1.26) |
| PR-negative | Work schedule involving night/rollover shift [a] | | | | |
| | Unexposed (day work only) | 217 | 3 | 1.00 (reference) | 1.00 (reference) |
| | Exposed (night/rollover shift) | 150 | 5 | 2.10 (0.55, 8.08) | 1.69 (0.58, 4.87) |
| HER2-positive | Work schedule involving night/rollover shift [a] | | | | |
| | Unexposed (day work only) | 217 | 7 | 1.00 (reference) | 1.00 (reference) |
| | Exposed (night/rollover shift) | 150 | 1 | 0.29 (0.04, 2.14) | 0.22 (0.05, 0.99) |
| HER2-negative | Work schedule involving night/rollover shift [a] | | | | |
| | Unexposed (day work only) | 217 | 21 | 1.00 (reference) | 1.00 (reference) |
| | Exposed (night/rollover shift) | 150 | 14 | 1.02 (0.49, 2.15) | 0.88 (0.47, 1.62) |
| Luminal A | Work schedule involving night/rollover shift [a] | | | | |
| | Unexposed (day work only) | 217 | 17 | 1.00 (reference) | 1.00 (reference) |
| | Exposed (night/rollover shift) | 150 | 11 | 0.92 (0.39, 2.14) | 0.69 (0.33, 1.46) |
| Luminal B | Work schedule involving night/rollover shift [a] | | | | |
| | Unexposed (day work only) | 217 | 5 | 1.00 (reference) | 1.00 (reference) |
| | Exposed (night/rollover shift) | 150 | 1 | 0.38 (0.05, 2.70) | 0.25 (0.05, 1.13) |
| Triple negative | Work schedule involving night/rollover shift [a] | | | | |
| | Unexposed (day work only) | 217 | 3 | 1.00 (reference) | 1.00 (reference) |
| | Exposed (night/rollover shift) | 150 | 2 | 1.36 (0.16, 11) | 2.23 (0.58, 8.55) |

Abbreviations: No=number, HR=hazard ratio, CI=confidence interval, ER=oestrogen receptor, PR=progesterone receptor, HER2=human epidermal growth factor receptor 2

[a] Adjusted for age, age at first child, number of children, menopause status, and education

For peer review only



For peer review only - http://bmjopen.bmj.com/site/about/guidelines.xhtml

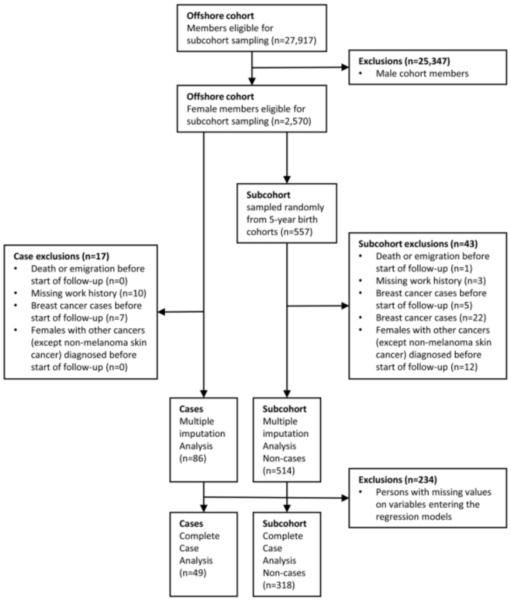


Figure 1. Overview of study design and exclusions

275x190mm (300 x 300 DPI)

**SUPPLEMENTAL MATERIAL**

**Content:**

- Methods' extension on multiple imputations
- Table S1
- Table S2
- Table S3
- Table S4
- Directed acyclic graphs (DAGs)
  - Figure S1
  - Figure S2

For peer review only



For peer review only - http://bmjopen.bmj.com/site/about/guidelines.xhtml

## Methods' extension

Multiple imputations

*Complete case analysis (n=367)*

As shown in Figure 1, the study sample for our complete case analysis included 49 breast cancer cases (with complete information on all variables in the regression model).

*Analysis of imputed data (n=600)*

As shown in Figure 1, our study sample yielded 86 breast cancer cases when those missing data retained. In this sample, 39% (n=233) had missing data on one or more of the following variables (missing numbers are displayed per variable):

- Work schedule involving night/rollover shift: n=32
- Duration of night/rollover shift: n=32
- Number of children: n=8
- Age at first child: n=192
- Education: n=8
- Main occupational activity in last position: n=7

Assuming missing at random, we used multiple imputation with chained equations and followed a 2-steps procedure:

1. We identified differences between these samples for date of birth, total employment duration, and breast cancer diagnosis, and used these variables as predictors together with the variables from the estimation model (i.e. work schedule involving night/rollover shift, duration of night/rollover shift, number of children, age at first child, education, and main



For peer review only - http://bmjopen.bmj.com/site/about/guidelines.xhtml

occupational activity in last position) to simultaneously predict missing data on all variables. Linear and multinomial logit models were used to predict missing data for continuous and categorical variables, respectively. The imputation model was ran 45 times, using the rule of thumb of running at least as many times as the percentage of missing data (White et al., 2011).

2. After step 1, we replicated the complete case analyses shown in Table 2, Table 3, and Table 4 on the imputed dataset (n=600).

In a sensitivity analysis (Table S4), we also tried an alternative imputation model where we excluded the variable 'age at first child', which had the highest number of missing (n=192, 39%). This reduced the total number of missing to n=53 (9%), but did not changed the results materially (Table S4).

Reference

White IR, Royston P, Wood AM. Multiple imputation using chained equations: Issues and guidance for practice. Statistics in medicine 2011; 30: 377-99.



For peer review only - http://bmjopen.bmj.com/site/about/guidelines.xhtml

# Table S1

| **Table S1.** Characteristics of Female Breast Cancer Cases in the Norwegian Offshore Petroleum Workers (NOPW) Cohort. | |
|---|---|
| | **Breast Cancer Cases [a] (n=86)** |
| Age at diagnosis, mean (range) | 56 (38-80) |
| Clinical stage, n (%) | |
| I: No metastasis | 38 (44) |
| II: Regional lymph node metastasis | 29 (34) |
| III: Local infiltration into skin | 12 (14) |
| IV: Distant metastasis | 4 (5) |
| Unspecified | 3 (3) |
| Oestrogen receptors (ER), n (%) | |
| ER-positive | 67 (78) |
| ER-negative | 13 (15) |
| Missing | 6 (7) |
| Progesterone receptors (PR), n (%) | |
| PR-positive | 59 (69) |
| PR-negative | 21 (24) |
| Missing | 6 (7) |
| Human epidermal growth factor receptor-2 (HER2), n (%) | |
| HER2-positive | 12 (14) |
| HER2-negative | 60 (70) |
| Missing | 14 (16) |
| Luminal A: HER2-negative and ER-positive and/or PR-positive, n (%) | |
| Yes | 46 (54) |
| No | 37 (43) |
| Missing | 3 (3) |
| Luminal B: HER2-positive and ER-positive and/or PR-positive, n (%) | |
| Yes | 10 (12) |
| No | 73 (85) |
| Missing | 3 (3) |
| Triple negative: HER2-negative and ER-negative and PR-negative, n (%) | |
| Yes | 11 (13) |
| No | 72 (84) |
| Missing | 3 (3) |
| [a] According to International Classification of Diseases $10^{th}$ edition : C50 | |

For peer review only



For peer review only - http://bmjopen.bmj.com/site/about/guidelines.xhtml

# Table S2

| **Table S2**. Hazard Ratios of Breast Cancer According to duration of night + rollover shift quartile in the Norwegian Offshore Petroleum Workers (NOPW) Cohort | | | | |
|---|---|---|---|---|
| | **Complete case analysis (n=367)** | | | **Multiple imputation (n=600; 86 cases)** |
| **Work schedule variable** | **No. of participants** | **No. of cases** | **HR (95% CI)** | **HR (95% CI)** |
| Duration of night/rollover shift [a,b] | | | | |
| Unexposed (0 years, day work only) | 217 | 29 | 1.00 (reference) | 1.00 (reference) |
| Quartile 1 night/rollover (<1-2 years) | 28 | 3 | 0.99 (0.28, 3.52) | 0.78 (0.28, 2.14) |
| Quartile 2 night/rollover (>2-5 years) | 32 | 2 | 0.52 (0.11, 2.47) | 0.49 (0.14, 1.71) |
| Quartile 3 night/rollover (>5-10 years) | 36 | 4 | 0.77 (0.24, 2.48) | 1.02 (0.47, 2.24) |
| Quartile 4 night/rollover (>10 years) | 54 | 11 | 1.62 (0.73, 3.59) | 1.10 (0.51, 2.37) |
| P-trend [c] | | | 0.506 | 0.944 |
| Abbreviations: No=number, HR=hazard ratio, CI=confidence interval | | | | |
| [a] Adjusted for age, age at first child, number of children, menopause status, and education | | | | |
| [b] complete work history i.e. up to 8 employments as an offshore worker | | | | |
| [c] Modelled as a continuous variable to test for linear trend | | | | |

For peer review only

# Table S3

**Table S3**. Hazard Ratios of Breast Cancer According to Work Schedule and Chemical Co-exposures in the Norwegian Offshore Petroleum Workers (NOPW) Cohort, multiple imputation analysis (n=601)

| | Chlorinated degreasers [a] | | |
|---|---|---|---|
| **Work schedule variable [b,d]** | **Unexposed** | **Exposed** | **P Value** |
| Unexposed (day work only) | | | |
| n (%) | 99 (17) | 223 (39) | |
| HR [c] (95% CI) | 1.00 (reference) | 0.91 (0.45, 1.84) | |
| Exposed (night/rollover shift) | | | |
| n (%) | 43 (8) | 204 (36) | |
| HR [c] (95% CI) | 1.01 (0.40, 2.55) | 0.79 (0.37, 1.71) | |
| $P_{interaction}$ | | | 0.725 |
| | **Benzene** | | |
| **Work schedule variable [b,d]** | **Unexposed** | **Exposed** | **P Value** |
| Unexposed (day work only) | | | |
| n (%) | 269 (48) | 53 (9) | |
| HR [c] (95% CI) | 1.00 (reference) | 0.60 (0.21, 1.70) | |
| Exposed (night/rollover shift) | | | |
| n (%) | 199 (35) | 48 (8) | |
| HR [c] (95% CI) | 0.74 (0.41, 1.32) | 1.11 (0.46, 2.70) | |
| $P_{interaction}$ | | | 0.175 |

Abbreviations: HR=hazard ratio, CI=confidence interval

[a] Importantly, the exposure assessment of chlorinated degreasers was probability-based by experts and evaluated as “possible” for catering workers.

[b] Complete work history i.e. up to 8 employments as an offshore worker

[c] Adjusted for age, age at first child, number of children, menopause status, and education

[d] Missing: n=32 for both chlorinated degreasers and benzene.

For peer review only



For peer review only - http://bmjopen.bmj.com/site/about/guidelines.xhtml

# Table S4

| **Table S4**. Hazard Ratios of Female Breast Cancer According to Work Schedule in the Norwegian Offshore Petroleum Workers (NOPW) Cohort. Sensitivity Analyses of models adjusted for age at first child *versus* models without adjustment for age at first child. | | |
|---|---|---|
| | **Multiple imputation, including 'age at first child' (n=600; 86 cases)** [a,b] | **Multiple imputation, excluding 'age at first child' (n=600; 86 cases)** [b,c] |
| **Work schedule variable** | **HR (95% CI)** | **HR (95% CI)** |
| Total employment duration | | |
| Quartile 1 (0-1.9 years) | 1.00 (reference) | 1.00 (reference) |
| Quartile 2 (2-5.9 years) | 1.50 (0.69, 3.27) | 1.53 (0.71, 3.31) |
| Quartile 3 (6-10.9 years) | 1.64 (0.78, 3.50) | 1.65 (0.78, 3.47) |
| Quartile 4 (11-24 years) | 1.19 (0.54, 2.61) | 1.22 (0.56, 2.67) |
| P-trend [d] | 0.733 | 0.685 |
| Work schedule involving night/rollover shift | | |
| Unexposed (day work only) | 1.00 (reference) | 1.00 (reference) |
| Exposed (night/rollover shift) | 0.87 (0.52, 1.46) | 0.87 (0.52, 1.45) |
| | | |
| Duration of night/rollover shift | | |
| Unexposed (0 years, day work only) | 1.00 (reference) | 1.00 (reference) |
| ≤Median night/rollover (<1-6 years) | 0.56 (0.26, 1.22) | 0.55 (0.25, 1.19) |
| >Median night/rollover (>6 years) | 1.21 (0.67, 2.18) | 1.21 (0.67, 2.17) |
| P-trend [d] | 0.748 | 0.752 |
| Abbreviations: HR=hazard ratio, CI=confidence interval | | |
| [a] Adjusted for age, age at first child, number of children, menopause status, and education. 39% (n=233) missing data imputed. | | |
| [b] complete work history i.e. up to 8 employments as an offshore worker | | |
| [c] Adjusted for variable in footnote "a" but without age at first child. 9% (n=53) missing data imputed. | | |
| [d] Modelled as a continuous variable to test for linear trend | | |

For peer review only



For peer review only - http://bmjopen.bmj.com/site/about/guidelines.xhtml

**Directed acyclic graphs (DAGs)**

DAGs were produced by the DAGitty v3.0 software (Textor et al., 2016). Please note that the green circle denotes the exposure, the blue circle with I denotes the outcome, the red circles denote the ancestor of the exposure AND the outcome (i.e. confounders, which are included in the models), and blue circles denote ancestor of the outcome (not included in the models).

<u>Reference</u>

Textor J, Zander B, Gilthorpe MK, Liskiewicz M, Ellison G. Robust causal inference using directed acyclic graphs: the R package 'dagitty'. Int J Epidemiol. 2016;45(6):1887-1894.
Website: www.dagitty.net

<u>Models estimating breast cancer risk in relation to work schedule</u>

In the following, we show DAG for each model with breast cancer as the outcome (as displayed in Tables 2 and 4) in relation to work schedule, age, menopausal status, age at first child, number of children, and education. We made the following assumptions:

- Age and education were not caused by any of the other variables in the DAG
- Menopausal status was affected by age
- Age at first child was affected by education
- Number of children was affected by age at first child

For peer review only

**Figure S1**

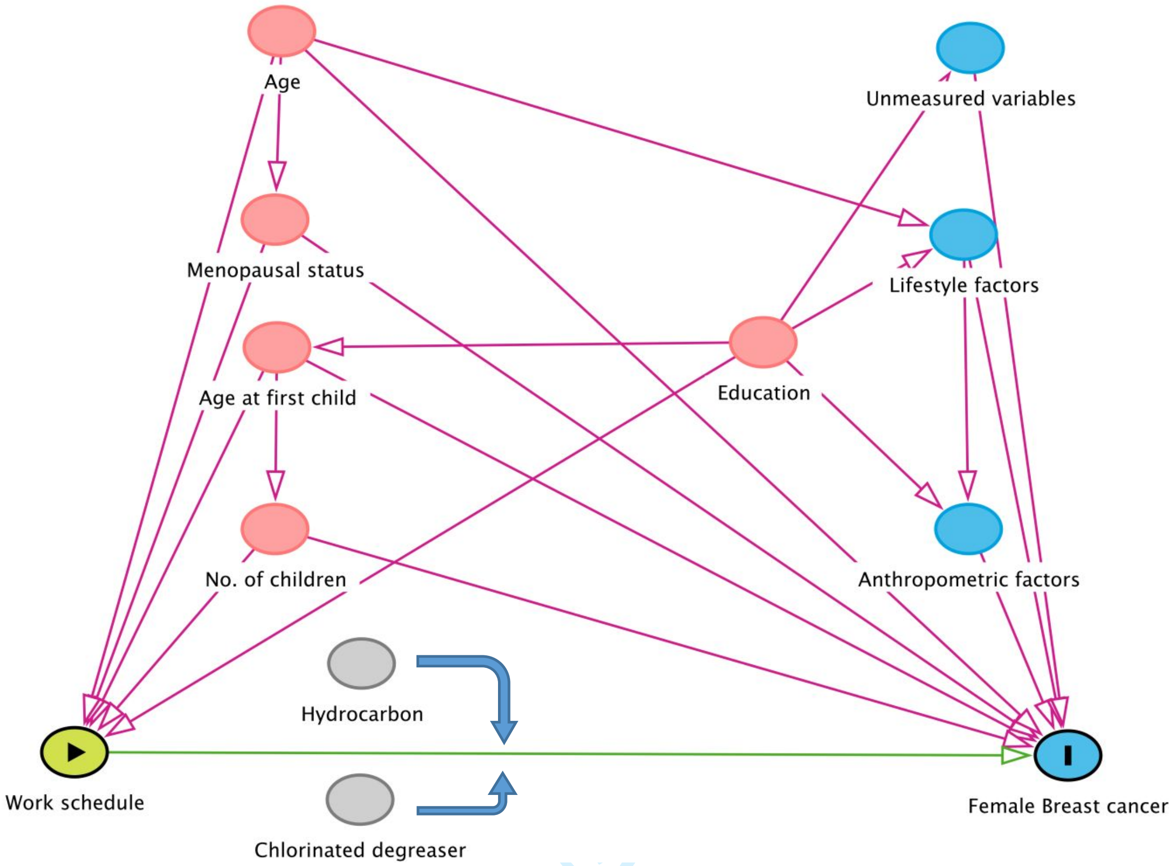


| Lifestyle factors: | Antropometric factors: | Unmeasured variables: |
|---|---|---|
| Fibre intake | Height | Chronotype |
| Multivitamine intake | Weight | Exogenous hormones use |
| Dietary fat intake | | Breast feeding |
| Alcohol intake | | Family history |
| Smoking | | |
| Physical activity | | |



For peer review only - http://bmjopen.bmj.com/site/about/guidelines.xhtml

Models estimating breast cancer risk in relation to chemical co-exposures

In the following, we show DAG for each model with breast cancer as the outcome (as displayed in Tables 3) in relation to age, education, main occupational activity in last position. We made the following assumptions:

- Age, and education were not caused by any of the other variables in the DAG
- Main occupational activity in last position was affected by education, and is an instrument of chemical co-exposure.

For peer review only



For peer review only - http://bmjopen.bmj.com/site/about/guidelines.xhtml

**Figure S2**

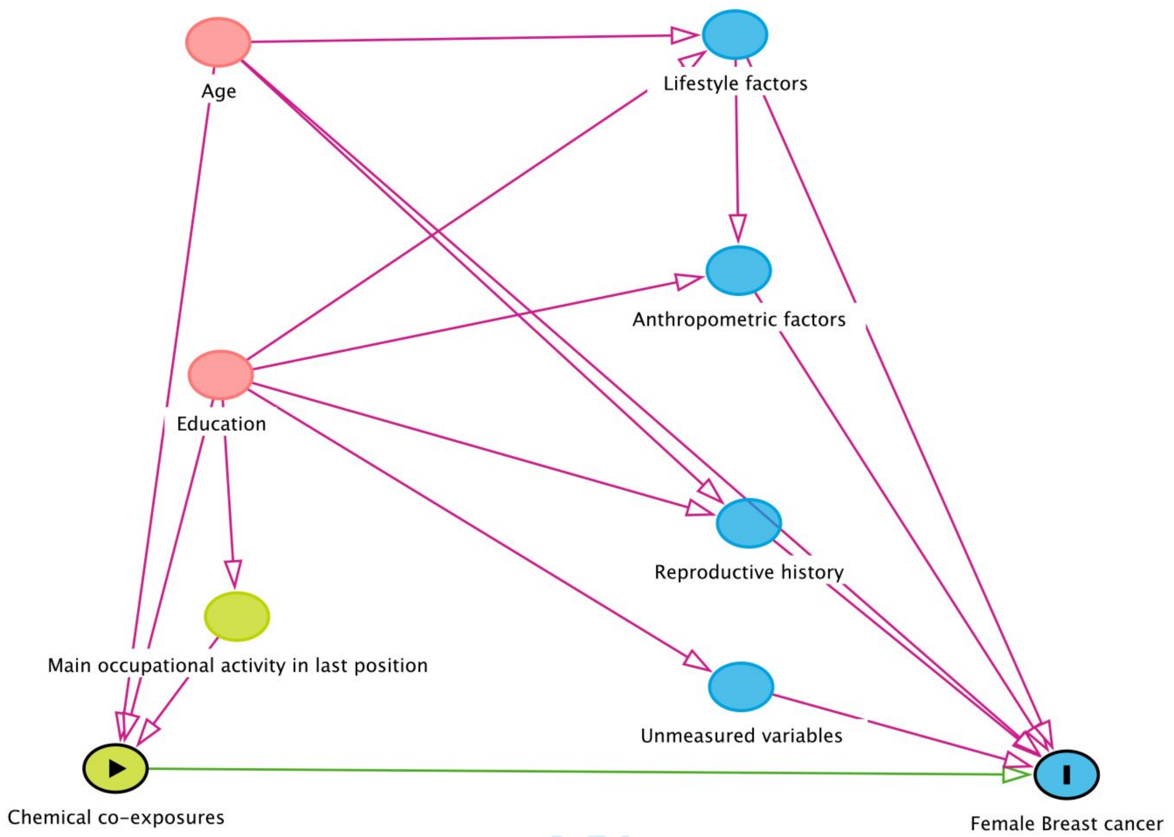


| **Chemical co-exposures**: | **Lifestyle factors:** | **Antropometric factors:** | **Reproductive history:** | **Unmeasured variables:** |
|---|---|---|---|---|
| Chlorinated degreaser | Fibre intake | Height | Age at first child | Exogenous hormones use |
| Benzene | Multivitamine intake | Weight | Number of children | Breast feeding |
| | Dietary fat intake | | Menopausal status | Family history |
| | Alcohol intake | | | |
| | Smoking | | | |
| | Physical activity | | | |



For peer review only - http://bmjopen.bmj.com/site/about/guidelines.xhtml

# SUPPLEMENTAL MATERIAL

**Content:**

- Methods’ extension on multiple imputations
- Table S1
- Table S2
- Table S3
- Table S4
- Directed acyclic graphs (DAGs)
    - Figure S1
    - Figure S2

For peer review only



For peer review only - http://bmjopen.bmj.com/site/about/guidelines.xhtml

## Methods' extension

### Multiple imputations

*Complete case analysis (n=367)*

As shown in Figure 1, the study sample for our complete case analysis included 49 breast cancer cases (with complete information on all variables in the regression model).

*Analysis of imputed data (n=600)*

As shown in Figure 1, our study sample yielded 86 breast cancer cases when those missing data retained. In this sample, 39% (n=233) had missing data on one or more of the following variables (missing numbers are displayed per variable):

- Work schedule involving night/rollover shift: n=32
- Duration of night/rollover shift: n=32
- Number of children: n=8
- Age at first child: n=192
- Education: n=8
- Main occupational activity in last position: n=7

Assuming missing at random, we used multiple imputation with chained equations and followed a 2-steps procedure:

1. We identified differences between these samples for date of birth, total employment duration, and breast cancer diagnosis, and used these variables as predictors together with the variables from the estimation model (i.e. work schedule involving night/rollover shift, duration of night/rollover shift, number of children, age at first child, education, and main



For peer review only - http://bmjopen.bmj.com/site/about/guidelines.xhtml

occupational activity in last position) to simultaneously predict missing data on all variables. Linear and multinomial logit models were used to predict missing data for continuous and categorical variables, respectively. The imputation model was ran 45 times, using the rule of thumb of running at least as many times as the percentage of missing data (White et al., 2011).

2. After step 1, we replicated the complete case analyses shown in Table 2, Table 3, and Table 4 on the imputed dataset (n=600).

In a sensitivity analysis (Table S4), we also tried an alternative imputation model where we excluded the variable 'age at first child', which had the highest number of missing (n=192, 39%). This reduced the total number of missing to n=53 (9%), but did not changed the results materially (Table S4).

Reference

White IR, Royston P, Wood AM. Multiple imputation using chained equations: Issues and guidance for practice. Statistics in medicine 2011; 30: 377-99.

For peer review only



For peer review only - http://bmjopen.bmj.com/site/about/guidelines.xhtml

# Table S1

| **Table S1.** Characteristics of Female Breast Cancer Cases in the Norwegian Offshore Petroleum Workers (NOPW) Cohort. | |
|---|---|
| | **Breast Cancer Cases [a] (n=86)** |
| Age at diagnosis, mean (range) | 56 (38-80) |
| Clinical stage, n (%) | |
| I: No metastasis | 38 (44) |
| II: Regional lymph node metastasis | 29 (34) |
| III: Local infiltration into skin | 12 (14) |
| IV: Distant metastasis | 4 (5) |
| Unspecified | 3 (3) |
| Oestrogen receptors (ER), n (%) | |
| ER-positive | 67 (78) |
| ER-negative | 13 (15) |
| Missing | 6 (7) |
| Progesterone receptors (PR), n (%) | |
| PR-positive | 59 (69) |
| PR-negative | 21 (24) |
| Missing | 6 (7) |
| Human epidermal growth factor receptor-2 (HER2), n (%) | |
| HER2-positive | 12 (14) |
| HER2-negative | 60 (70) |
| Missing | 14 (16) |
| Luminal A: HER2-negative and ER-positive and/or PR-positive, n (%) | |
| Yes | 46 (54) |
| No | 37 (43) |
| Missing | 3 (3) |
| Luminal B: HER2-positive and ER-positive and/or PR-positive, n (%) | |
| Yes | 10 (12) |
| No | 73 (85) |
| Missing | 3 (3) |
| Triple negative: HER2-negative and ER-negative and PR-negative, n (%) | |
| Yes | 11 (13) |
| No | 72 (84) |
| Missing | 3 (3) |
| [a] According to International Classification of Diseases 10th edition : C50 | |

For peer review only

# Table S2

| **Table S2**. Hazard Ratios of Breast Cancer According to duration of night + rollover shift quartile in the Norwegian Offshore Petroleum Workers (NOPW) Cohort | | | | |
|---|---|---|---|---|
| | **Complete case analysis (n=367)** | | | **Multiple imputation (n=~~601~~600; 86 cases)** |
| **Work schedule variable** | **No. of participants** | **No. of cases** | **HR (95% CI)** | **HR (95% CI)** |
| Duration of night/rollover shift [a,b] | | | | |
| Unexposed (0 years, day work only) | 217 | 29 | 1.00 (reference) | 1.00 (reference) |
| Quartile 1 night/rollover (<1-2 years) | 28 | 3 | 0.99 (0.28, 3.52) | 0.78 (0.28, 2.14) |
| Quartile 2 night/rollover (>2-5 years) | 32 | 2 | 0.52 (0.11, 2.47) | 0.49 (0.14, 1.71) |
| Quartile 3 night/rollover (>5-10 years) | 36 | 4 | 0.77 (0.24, 2.48) | 1.02 (0.47, 2.24) |
| Quartile 4 night/rollover (>10 years) | 54 | 11 | 1.62 (0.73, 3.59) | 1.10 (0.51, 2.37) |
| P-trend [c] | | | 0.506 | 0.944 |
| Abbreviations: No=number, HR=hazard ratio, CI=confidence interval | | | | |
| [a] Adjusted for age, age at first child, number of children, menopause status, and education | | | | |
| [b] complete work history i.e. up to 8 employments as an offshore worker | | | | |
| [c] Modelled as a continuous variable to test for linear trend | | | | |



For peer review only - http://bmjopen.bmj.com/site/about/guidelines.xhtml

# Table S3

| **Table S3**. Hazard Ratios of Breast Cancer According to Work Schedule and Chemical Co-exposures in the Norwegian Offshore Petroleum Workers (NOPW) Cohort, multiple imputation analysis (n=601) | | | |
|---|---|---|---|
| | **Chlorinated degreasers [a]** | | |
| **Work schedule variable [b,d]** | **Unexposed** | **Exposed** | **P Value** |
| Unexposed (day work only) | | | |
| n (%) | 99 (17) | 223 (39) | |
| HR [c] (95% CI) | 1.00 (reference) | 0.91 (0.45, 1.84) | |
| Exposed (night/rollover shift) | | | |
| n (%) | 43 (8) | 204 (36) | |
| HR [c] (95% CI) | 1.01 (0.40, 2.55) | 0.79 (0.37, 1.71) | |
| $P_{interaction}$ | | | 0.725 |
| | **Benzene** | | |
| **Work schedule variable [b,d]** | **Unexposed** | **Exposed** | **P Value** |
| Unexposed (day work only) | | | |
| n (%) | 269 (48) | 53 (9) | |
| HR [c] (95% CI) | 1.00 (reference) | 0.60 (0.21, 1.70) | |
| Exposed (night/rollover shift) | | | |
| n (%) | 199 (35) | 48 (8) | |
| HR [c] (95% CI) | 0.74 (0.41, 1.32) | 1.11 (0.46, 2.70) | |
| $P_{interaction}$ | | | 0.175 |
| Abbreviations: HR=hazard ratio, CI=confidence interval | | | |
| [a] Importantly, the exposure assessment of chlorinated degreasers was probability-based by experts and evaluated as "possible" for catering workers. | | | |
| [b] Complete work history i.e. up to 8 employments as an offshore worker | | | |
| [c] Adjusted for age, age at first child, number of children, menopause status, and education | | | |
| [d] Missing: n=32 for both chlorinated degreasers and benzene. | | | |



For peer review only - http://bmjopen.bmj.com/site/about/guidelines.xhtml

# Table S4

| **Table S4**. Hazard Ratios of Female Breast Cancer According to Work Schedule in the Norwegian Offshore Petroleum Workers (NOPW) Cohort. Sensitivity Analyses of models adjusted for age at first child *versus* models without adjustment for age at first child. | | |
|---|---|---|
| | **Multiple imputation, including 'age at first child' (n=600; 86 cases)** [a,b] | **Multiple imputation, excluding 'age at first child' (n=600; 86 cases)** [b,c] |
| **Work schedule variable** | **HR (95% CI)** | **HR (95% CI)** |
| Total employment duration | | |
| Quartile 1 (0-1.9 years) | 1.00 (reference) | 1.00 (reference) |
| Quartile 2 (2-5.9 years) | 1.50 (0.69, 3.27) | 1.53 (0.71, 3.31) |
| Quartile 3 (6-10.9 years) | 1.64 (0.78, 3.50) | 1.65 (0.78, 3.47) |
| Quartile 4 (11-24 years) | 1.19 (0.54, 2.61) | 1.22 (0.56, 2.67) |
| P-trend [d] | 0.733 | 0.685 |
| Work schedule involving night/rollover shift | | |
| Unexposed (day work only) | 1.00 (reference) | 1.00 (reference) |
| Exposed (night/rollover shift) | 0.87 (0.52, 1.46) | 0.87 (0.52, 1.45) |
| | | |
| Duration of night/rollover shift | | |
| Unexposed (0 years, day work only) | 1.00 (reference) | 1.00 (reference) |
| ≤Median night/rollover (<1-6 years) | 0.56 (0.26, 1.22) | 0.55 (0.25, 1.19) |
| >Median night/rollover (>6 years) | 1.21 (0.67, 2.18) | 1.21 (0.67, 2.17) |
| P-trend [d] | 0.748 | 0.752 |
| Abbreviations: HR=hazard ratio, CI=confidence interval | | |
| [a] Adjusted for age, age at first child, number of children, menopause status, and education. 39% (n=233) missing data imputed. | | |
| [b] complete work history i.e. up to 8 employments as an offshore worker | | |
| [c] Adjusted for variable in footnote "a" but without age at first child. 9% (n=53) missing data imputed. | | |
| [d] Modelled as a continuous variable to test for linear trend | | |

For peer review only

**Directed acyclic graphs (DAGs)**

DAGs were produced by the DAGitty v3.0 software (Textor et al., 2016). Please note that the green circle denotes the exposure, the blue circle with I denotes the outcome, the red circles denote the ancestor of the exposure AND the outcome (i.e. confounders, which are included in the models), and blue circles denote ancestor of the outcome (not included in the models).

Reference
Textor J, Zander B, Gilthorpe MK, Liskiewicz M, Ellison G. Robust causal inference using directed acyclic graphs: the R package 'dagitty'. Int J Epidemiol. 2016;45(6):1887-1894.
Website: www.dagitty.net

Models estimating breast cancer risk in relation to work schedule
In the following, we show DAG for each model with breast cancer as the outcome (as displayed in Tables 2 and 4) in relation to work schedule, age, menopausal status, age at first child, number of children, and education. We made the following assumptions:

- Age and education were not caused by any of the other variables in the DAG
- Menopausal status was affected by age
- Age at first child was affected by education
- Number of children was affected by age at first child

For peer review only

**Figure S1**

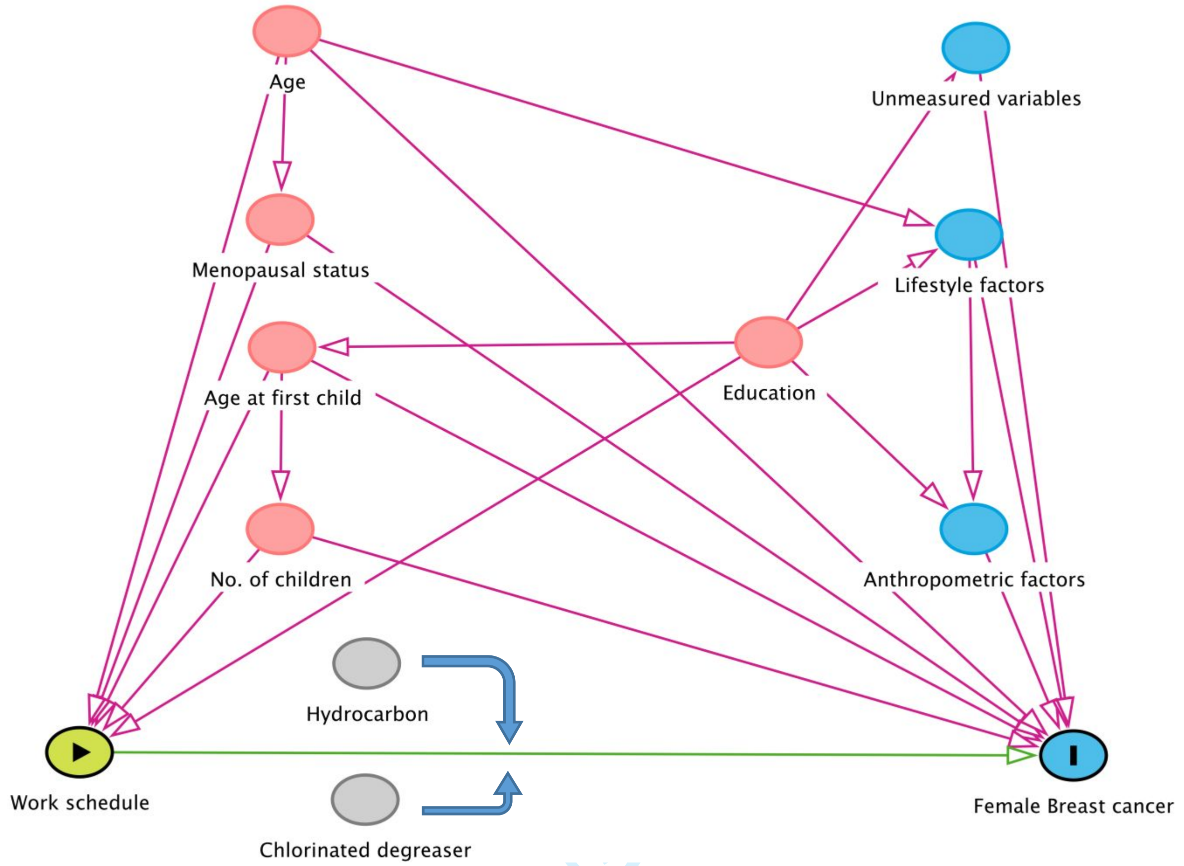


| Lifestyle factors: | Antropometric factors: | Unmeasured variables: |
|---|---|---|
| Fibre intake | Height | Chronotype |
| Multivitamine intake | Weight | Exogenous hormones use |
| Dietary fat intake | | Breast feeding |
| Alcohol intake | | Family history |
| Smoking | | |
| Physical activity | | |



For peer review only - http://bmjopen.bmj.com/site/about/guidelines.xhtml

Models estimating breast cancer risk in relation to chemical co-exposures

In the following, we show DAG for each model with breast cancer as the outcome (as displayed in Tables 3) in relation to age, education, main occupational activity in last position. We made the following assumptions:

- Age, and education were not caused by any of the other variables in the DAG
- Main occupational activity in last position was affected by education, and is an instrument of chemical co-exposure.

For peer review only



For peer review only - http://bmjopen.bmj.com/site/about/guidelines.xhtml

**Figure S2**

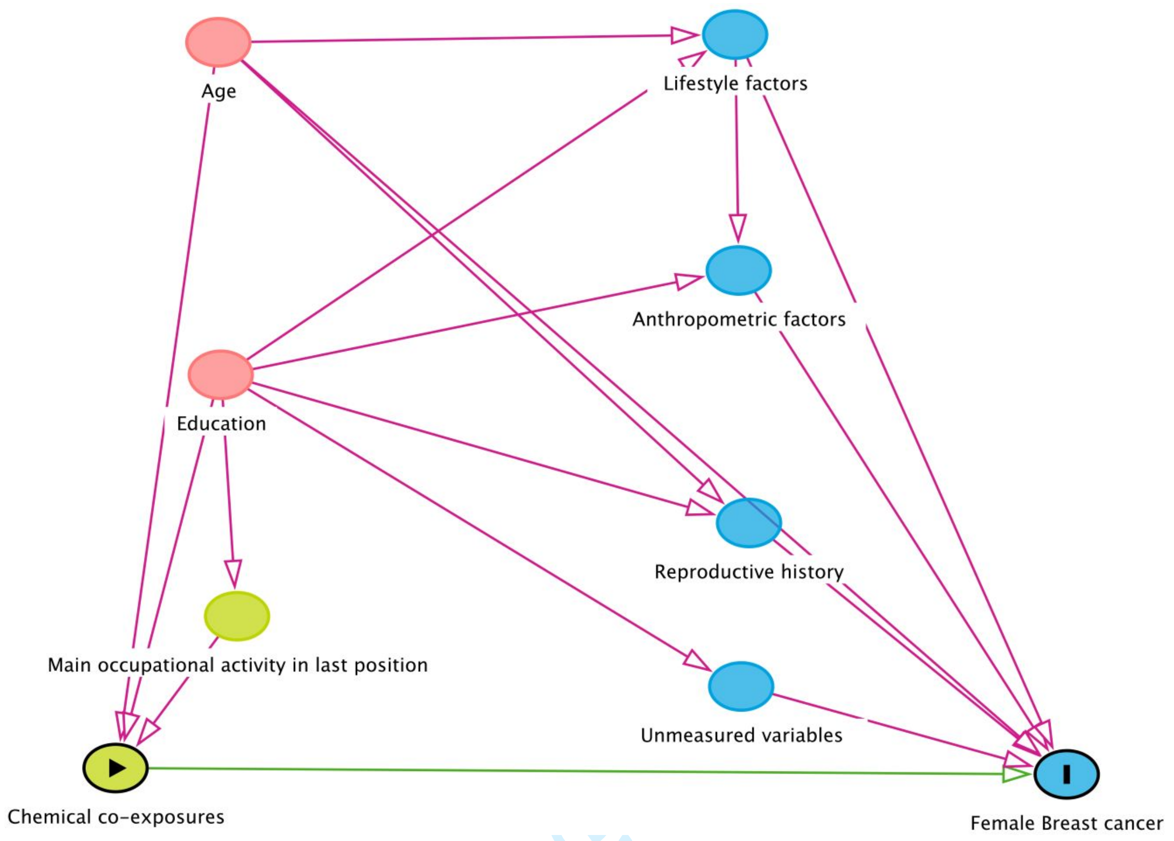


| **Chemical co-exposures**: | **Lifestyle factors:** | **Antropometric factors:** | **Reproductive history:** | **Unmeasured variables:** |
|---|---|---|---|---|
| Chlorinated degreaser | Fibre intake | Height | Age at first child | Exogenous hormones use |
| Benzene | Multivitamine intake | Weight | Number of children | Breast feeding |
| | Dietary fat intake | | Menopausal status | Family history |
| | Alcohol intake | | | |
| | Smoking | | | |
| | Physical activity | | | |



For peer review only - http://bmjopen.bmj.com/site/about/guidelines.xhtml

STROBE Statement—Checklist of items that should be included in reports of ***cohort studies***

| | Item No | Recommendation | Page No |
|---|---|---|---|
| **Title and abstract** | 1 | (*a*) Indicate the study's design with a commonly used term in the title or the abstract<br>(*b*) Provide in the abstract an informative and balanced summary of what was done and what was found | p. 1-2 |
| **Introduction** | | | |
| Background/rationale | 2 | Explain the scientific background and rationale for the investigation being reported | p. 4-5 |
| Objectives | 3 | State specific objectives, including any prespecified hypotheses | p. 5 |
| **Methods** | | | |
| Study design | 4 | Present key elements of study design early in the paper | p. 6 |
| Setting | 5 | Describe the setting, locations, and relevant dates, including periods of recruitment, exposure, follow-up, and data collection | p. 5-6 |
| Participants | 6 | (*a*) Give the eligibility criteria, and the sources and methods of selection of participants. Describe methods of follow-up<br>(*b*) For matched studies, give matching criteria and number of exposed and unexposed | p. 7-8 |
| Variables | 7 | Clearly define all outcomes, exposures, predictors, potential confounders, and effect modifiers. Give diagnostic criteria, if applicable | p. 6-10 |
| Data sources/ measurement | 8* | For each variable of interest, give sources of data and details of methods of assessment (measurement). Describe comparability of assessment methods if there is more than one group | p. 6-10 |
| Bias | 9 | Describe any efforts to address potential sources of bias | p. 7-11 |
| Study size | 10 | Explain how the study size was arrived at | p. 7-8, Figure 1 |
| Quantitative variables | 11 | Explain how quantitative variables were handled in the analyses. If applicable, describe which groupings were chosen and why | p. 6-11 |
| Statistical methods | 12 | (*a*) Describe all statistical methods, including those used to control for confounding<br>(*b*) Describe any methods used to examine subgroups and interactions<br>(*c*) Explain how missing data were addressed<br>(*d*) If applicable, explain how loss to follow-up was addressed<br>(*e*) Describe any sensitivity analyses | p. 10-11 |
| **Results** | | | |
| Participants | 13* | (a) Report numbers of individuals at each stage of study—eg numbers potentially eligible, examined for eligibility, confirmed eligible, included in the study, completing follow-up, and analysed<br>(b) Give reasons for non-participation at each stage<br>(c) Consider use of a flow diagram | p. 7-8, Figure 1 |
| Descriptive data | 14* | (a) Give characteristics of study participants (eg demographic, clinical, social) and information on exposures and potential confounders | p. 11-12, Table 1 + Table S1 |


For peer review only - http://bmjopen.bmj.com/site/about/guidelines.xhtml

| | | | |
|---|---|---|---|
| | | (b) Indicate number of participants with missing data for each variable of interest<br>(c) Summarise follow-up time (eg, average and total amount) | |
| Outcome data | 15* | Report numbers of outcome events or summary measures over time | p. 11-13, Figure 1 |

For peer review only


For peer review only - http://bmjopen.bmj.com/site/about/guidelines.xhtml

| | | | |
|---|---|---|---|
| Main results | 16 | (*a*) Give unadjusted estimates and, if applicable, confounder-adjusted estimates and their precision (eg, 95% confidence interval). Make clear which confounders were adjusted for and why they were included<br>(*b*) Report category boundaries when continuous variables were categorized<br>(*c*) If relevant, consider translating estimates of relative risk into absolute risk for a meaningful time period | p. 12, Table 2 |
| Other analyses | 17 | Report other analyses done—eg analyses of subgroups and interactions, and sensitivity analyses | p. 11-12, Table 3 + Table 4 + Table S2 + Table S3 + Table S4 |
| **Discussion** | | | |
| Key results | 18 | Summarise key results with reference to study objectives | p. 13 |
| Limitations | 19 | Discuss limitations of the study, taking into account sources of potential bias or imprecision. Discuss both direction and magnitude of any potential bias | p. 14-15 |
| Interpretation | 20 | Give a cautious overall interpretation of results considering objectives, limitations, multiplicity of analyses, results from similar studies, and other relevant evidence | p. 13-15 |
| Generalisability | 21 | Discuss the generalisability (external validity) of the study results | p. 15 |
| **Other information** | | | |
| Funding | 22 | Give the source of funding and the role of the funders for the present study and, if applicable, for the original study on which the present article is based | p. 16 |

*Give information separately for exposed and unexposed groups.

**Note:** An Explanation and Elaboration article discusses each checklist item and gives methodological background and published examples of transparent reporting. The STROBE checklist is best used in conjunction with this article (freely available on the Web sites of PLoS Medicine at http://www.plosmedicine.org/, Annals of Internal Medicine at http://www.annals.org/, and Epidemiology at http://www.epidem.com/). Information on the STROBE Initiative is available at http://www.strobe-statement.org.

For peer review only



For peer review only - http://bmjopen.bmj.com/site/about/guidelines.xhtml

BMJ Open

# Night shift work, chemical co-exposures and risk of female breast cancer in the Norwegian Offshore Petroleum Workers (NOPW) cohort: a prospectively-recruited case-cohort study.

| | |
|---|---|
| Journal: | *BMJ Open* |
| Manuscript ID | bmjopen-2021-056396.R1 |
| Article Type: | Original research |
| Date Submitted by the Author: | 04-Nov-2021 |
| Complete List of Authors: | Liu, Fei-Chih; Cancer Registry of Norway, Department of Research; University of Oslo Faculty of Medicine, Department of Biostatistics<br>Veierød, Marit ; University of Oslo Faculty of Medicine, Department of Biostatistics<br>Kjærheim, Kristina; Cancer Registry of Norway, Department of Research<br>Robsahm, Trude; Cancer Registry of Norway, Department of Research<br>Ghiasvand, Reza; Cancer Registry of Norway, Department of Research; Oslo University Hospital, Oslo Centre for Biostatistics and Epidemiology<br>Hosgood, Dean; Albert Einstein College of Medicine, Department of Epidemiology and Population Health<br>Samuelsen, Sven Ove; University of Oslo, Department of Mathematics<br>Bråtveit, Magne; Universitetet i Bergen Det medisinsk-odontologiske fakultet, Department of Global Public Health and Primary Care<br>Kirkeleit, Jorun; Universitetet i Bergen Det medisinsk-odontologiske fakultet, Department of Global Health and Primary Care<br>Rothman, Nat; National Cancer Institute, Occupational and Environmental Epidemiology<br>Lan, Qing; National Cancer Institute, Occupational and Environmental Epidemiology<br>Silverman, Debra; National Cancer Institute, Occupational and Environmental Epidemiology<br>Friesen, Melissa C.; National Cancer Institute, Occupational and Environmental Epidemiology<br>Babigumira, Ronnie; Cancer Registry of Norway, Department of Research<br>Shala, Nita; Cancer Registry of Norway, Department of Research; University of Oslo Faculty of Medicine, Department of Biostatistics<br>Grimsrud, Tom; Cancer Registry of Norway, Department of Research<br>Stenehjem, Jo; Cancer Registry of Norway, Department of Research |
| <b>Primary Subject Heading</b>: | Epidemiology |
| Secondary Subject Heading: | Oncology, Public health, Occupational and environmental medicine |
| Keywords: | EPIDEMIOLOGY, Breast tumours < ONCOLOGY, OCCUPATIONAL & INDUSTRIAL MEDICINE |
| | |

*I, the Submitting Author has the right to grant and does grant on behalf of all authors of the Work (as defined in the below author licence), an exclusive licence and/or a non-exclusive licence for contributions from authors who are: i) UK Crown employees; ii) where BMJ has agreed a CC-BY licence shall apply, and/or iii) in accordance with the terms applicable for US Federal Government officers or employees acting as part of their official duties; on a worldwide, perpetual, irrevocable, royalty-free basis to BMJ Publishing Group Ltd ("BMJ") its licensees and where the relevant Journal is co-owned by BMJ to the co-owners of the Journal, to publish the Work in this journal and any other BMJ products and to exploit all rights, as set out in our licence.*

*The Submitting Author accepts and understands that any supply made under these terms is made by BMJ to the Submitting Author unless you are acting as an employee on behalf of your employer or a postgraduate student of an affiliated institution which is paying any applicable article publishing charge ("APC") for Open Access articles. Where the Submitting Author wishes to make the Work available on an Open Access basis (and intends to pay the relevant APC), the terms of reuse of such Open Access shall be governed by a Creative Commons licence – details of these licences and which Creative Commons licence will apply to this Work are set out in our licence referred to above.*

*Other than as permitted in any relevant BMJ Author's Self Archiving Policies, I confirm this Work has not been accepted for publication elsewhere, is not being considered for publication elsewhere and does not duplicate material already published. I confirm all authors consent to publication of this Work and authorise the granting of this licence.*

For peer review only

## MAIN DOCUMENT

**Title:** Night shift work, chemical co-exposures and risk of female breast cancer in the Norwegian Offshore Petroleum Workers (NOPW) cohort: a prospectively-recruited case-cohort study.

**Authors:** Fei-Chih Liu[1,2], Marit Bragelien Veierød[2], Kristina Kjærheim[1], Trude Eid Robsahm[1], Reza Ghiasvand[1,3], H. Dean Hosgood[4], Sven Ove Samuelsen[5], Magne Bråtveit[6], Jorun Kirkeleit[6], Nathaniel Rothman[7], Qing Lan[7], Debra T. Silverman[7], Melissa C. Friesen[7], Ronnie Babigumira[1], Nita K. Shala[1,2], Tom K. Grimsrud[1], Jo S. Stenehjem[1].

1) Department of Research, Cancer Registry of Norway, Institute of Population based Cancer Research, Oslo, Norway.
2) Oslo Centre for Biostatistics and Epidemiology, Department of Biostatistics, Institute of Basic Medical Sciences, University of Oslo, Norway.
3) Oslo Centre for Biostatistics and Epidemiology, Oslo University Hospital, Norway.
4) Department of Epidemiology and Population Health, Albert Einstein College of Medicine, Bronx, NY, United States.
5) Department of Mathematics, University of Oslo, Oslo, Norway.
6) Department of Global Public Health and Primary Care, University of Bergen, Bergen, Norway.
7) Occupational and Environmental Epidemiology Branch, Division of Cancer Epidemiology and Genetics, National Cancer Institute, Bethesda, MD, United States

Address correspondence to:
Jo S. Stenehjem, PhD
Post: Cancer Registry of Norway, P.O. Box 5313 Majorstuen, NO-0304 Oslo, Norway
E-mail: jo.stenehjem@kreftregisteret.no
Phone: +47 905 09 570

Word-count: 222 (abstract) 3,519 (main text)

Number of references: 47

Number of tables and figures: 5

Conflict of interest: None declared.



For peer review only - http://bmjopen.bmj.com/site/about/guidelines.xhtml

## ABSTRACT

**Objectives:** This study examined the association between night shift work and risk of breast cancer, overall and by hormone receptor subtype, among females in the Norwegian Offshore Petroleum Workers (NOPW) cohort. We also examined the association of co-exposure (chlorinated degreasers and benzene) and breast cancer risk, and possible interaction with work schedule.

**Design:** Prospectively-recruited case-cohort study within the NOPW cohort.

**Setting:** Female offshore petroleum workers active on the Norwegian continental shelf.

**Participants**: 600 female workers (86 cases and 514 non-cases) were included in the study. We excluded workers that died or emigrated before start of follow-up, had missing work history, were diagnosed with breast cancer or other prior malignancy (except non-melanoma skin cancer) before start of follow-up.

**Results:** No overall association was found between breast cancer risk and work schedule (hazard ratio=0.87, 95% confidence interval: 0.52, 1.46 for work schedule involving night shift *vs*. day shift only). There was no significant association between work schedule and risk of any breast cancer subtype. No significant interactions were found between work schedule and chemical co-exposures (breast cancer overall $P_{interaction\ chlorinated\ degreasers}$=0.725 and $P_{interaction\ benzene}$=0.175).

**Conclusions:** Our results did not provide supporting evidence that work schedule involving night shift affects breast cancer risk in female offshore petroleum workers, but should be considered cautiously due to few cases. Further studies with larger sample sizes are warranted.




For peer review only - http://bmjopen.bmj.com/site/about/guidelines.xhtml

**Strengths and limitations of this study**

- Complete cancer and population registry data ensure complete follow-up.
- Comprehensive work history information of night/rollover shift duration for up to eight employments per worker.
- Job-exposure matrices of chemical exposure developed specifically for cancer studies in the cohort.
- Females in the cohort were young at baseline, which yielded relatively few breast cancer cases.
- We lacked information on family history of breast cancer, exogenous hormone use and chronotype.

For peer review only



For peer review only - http://bmjopen.bmj.com/site/about/guidelines.xhtml

## INTRODUCTION

Breast cancer is the most frequent cancer among females and the incidence is higher in industrialised compared to developing countries (1). The increase seen in industrialised societies has mainly been attributed to reproduction and lifestyle factors (2, 3). However, the use of artificial light during work at night, leading to disruption of the internal circadian clock and suppressed melatonin production, may accelerate the transformation of normal cells to malignant mammary cells and promote the risk of breast cancer (4, 5).

Norwegian offshore workers employed at oil and gas installations have an extreme touring work pattern (6). A standard two-week tour on Norwegian installations is minimum 12 hours of work seven days per week. The work schedule is either 14 day shifts, 14 night shifts or 14 rollover shifts (starting with 7 days followed by 7 nights or *vice versa*) (6). During a night shift, offshore workers are constantly exposed to artificial light (6). These extreme working hours meet the definition of night shift work (≥7 hours of work including the period from midnight to 05:00 hours) (7, 8).

The relationship between night shift work and breast cancer is still unclear. Several studies have reported a positive association between night shift work and breast cancer (9-12), while others did not (13-16). A challenge has been the lack of high-quality exposure data on light during night time and detailed data on night shift work schedules (4). A recent pooled analysis of five population-based case-control studies with complete work history reported an increased risk of breast cancer in pre-menopausal females who had high intensity and long duration of night shift work (17). Further, night shift work may be associated with an increased risk of oestrogen receptor (ER)-positive, progesterone receptor (PR)-positive or human epidermal growth factor receptor 2 (HER2)-positive breast cancer (18-21). However, night shift work has also been reported to increase the risk of ER-negative breast cancer (22),



For peer review only - http://bmjopen.bmj.com/site/about/guidelines.xhtml

For peer review only

and whether the association between night shift work and breast cancer varies by subtype remains unclear.

Based on the limited evidence for the carcinogenicity of night shift work, the International Agency for Research on Cancer (IARC) recently reconfirmed night shift work as a probable carcinogen to humans (Group 2A) (23), asking for improved exposure assessment in future studies to reduce heterogeneity between studies and allow for better comparisons. IARC also encouraged better reporting of potential co-exposures to occupational carcinogens. On offshore petroleum installations, some female workers may be exposed to a number of carcinogens, but in particular benzene during drilling, production and maintenance (24, 25), and historically chlorinated degreasers during cleaning of metal parts (trichloroethylene) or dry-cleaning of work clothes (tetrachloroethylene) (21). Cohort and population-based studies have linked benzene and tetrachloroethylene to increased breast cancer risk (26, 27), and recently, night shift work has received increased attention as a possible effect modifier of the association between chemical exposure and cancer by desynchronising detoxification mechanisms (28, 29). To our knowledge, chemical exposure and night shift work have not been examined simultaneously in relation to breast cancer risk.

The Norwegian Offshore Petroleum Workers (NOPW) cohort holds information on work schedule for each individual employed 1965–1998 and job exposure matrices (JEMs) capturing chemical exposures in the offshore work environment (30). The aim of the present study was to examine the association between shift work and breast cancer risk, overall and by receptor status subtypes, among prospectively-recruited females in the NOPW cohort. We also examined the association of chemical exposures (chlorinated degreasers and benzene) and breast cancer risk, and possible multiplicative interactions with work schedule.



For peer review only - http://bmjopen.bmj.com/site/about/guidelines.xhtml

# MATERIALS AND METHODS

## Patient and public involvement

This study was conducted without patient involvement.

## Study population and study design

The NOPW cohort was established in 1998 by the Cancer Registry of Norway (CRN). Questionnaires were filled in by current or former male and female offshore petroleum workers with a minimum of 20 days work on the Norwegian continental shelf between 1965 and 1998 (30). In total, 27,917 workers were included (estimated response rate 69%) in the NOPW cohort (31). Details on the cohort and its establishment have been published elsewhere (30, 31). All workers filled in an informed consent for participation in the study. Necessary legal and ethical approvals have been obtained from the Norwegian Data Inspectorate, the Regional Committee for Medical Research Ethics (2018/1162), and the Norwegian Directorate of Health.

We used a stratified case-cohort design (32) as complete information on work history had to be extracted manually from the questionnaires for breast cancer cases and a random subsample of the cohort (hereafter the 'subcohort'). The case-cohort design allows prospective models such as Cox-regression, and the subcohort may be used as controls for different types of cancer cases, as it is not matched to specific cases.

## Identification of cancer cases

The NOPW cohort was linked to the CRN and the National Population Register for information on cancer, death and emigration, by using the unique personal identification numbers assigned to all Norwegian citizens (33). Follow-up started 1 July 1999 and ended 31 December 2017. Reporting of incident cancers to the CRN is mandatory in Norway and the



For peer review only - http://bmjopen.bmj.com/site/about/guidelines.xhtml

degree of completeness and validity is high, with morphology verified for 99.3% of the breast cancers (33). Cancer cases were defined according to the International Classification of Diseases, Tenth Revision (ICD-10), C50 for breast cancer. Immunohistochemistry and pathology reports were submitted routinely to the CRN to assess breast cancer receptor status.

**Study sample**

A total of 25,347 males were excluded from the full cohort, and breast cancer cases and the subcohort were identified among the remaining 2,570 female offshore workers (Figure 1). Among the cases, we applied the exclusion criteria (i) death or emigration before start of follow-up (n=0) (ii) missing work history (n=10), (iii) breast cancer before start of follow-up (n=7), and (iv) other prior malignancy except non-melanoma skin cancer before start of follow-up (mainly squamous cell carcinoma as the CRN does not routinely record information on basal cell carcinoma of the skin [n=0]), resulting in 86 breast cancer cases in the analyses (Figure 1).

The initial subcohort (n=557) was sampled from the female cohort (n=2,570) and was established in two steps to secure at least 5 non-cases per case. First, we oversampled (i.e. selected all) the female cohort members born 1920–1939 and 1975–1979 (n=102) to secure comparable non-cases for the oldest and youngest cases. Second, for those born 1940–1974, we drew at random within strata of 5-year birth cohorts 455 female workers, who were frequency matched to the birth year distribution of all potentially occupational cancers estimated for 2017 (n=91). We excluded 43 females that died or emigrated before start of follow-up (n=1), had missing work history (n=3), were diagnosed with breast cancer (n=5) or other prior malignancy (except non-melanoma skin cancer [n=12]) before start of follow-up, or were diagnosed with breast cancer during follow-up (cases, n=22). Thus, for our analyses, 514 females remained in the subcohort (Figure 1).



For peer review only - http://bmjopen.bmj.com/site/about/guidelines.xhtml

**Exposure assessment**

*Work schedule*

We handled work history data of 514 female subcohort members and 86 cases who reported up to eight employments each. The process of harmonising overlapping employment records was handled by collapsing jobs within the same category and by splitting jobs of different categories into proportionally equal parts, according to a method described previously (34).

The self-reported work schedule for each job was recorded as either day, night, or rollover shift for two-week tours. Day shift is equivalent to 14 consecutive days of day-time work usually between 07:00 and 19:00 hours. Night shift is equivalent to 14 consecutive days of night-time work usually between 19:00 and 07:00 hours. Rollover shift is equivalent to 7 consecutive days of day-time work (same working hours as for day shift) then alternate to 7 consecutive days of night-time work (same working hours as for night shift), or *vice versa*.

Some worked only day, but none worked only night or only rollover. Therefore, the variable work schedule was categorised as 1) day work only, 2) mixed I (some night/rollover + mainly day), 3) mixed II (mainly night/rollover + some day), and 4) missing. We then collapsed categories 2 (mixed I) and 3 (mixed II) into exposed (night/rollover shift) to represent workers that had ever worked some degree of night-time. For each individual, the duration of night/rollover shift was calculated by (i) cumulating the total number of years with night shifts, (ii) cumulating the total number of years with rollover shifts, (iii) summarising the number of years worked with night and rollover shifts into a combined continuous variable, and (iv) categorising this variable according to median (unexposed, <1-6, >6) or quartile (unexposed, <1–2, >2–6, >6–10, >10) of years of night/rollover shift. Total employment duration was categorised according to quartiles (0–1.9, 2–5.9, 6–10.9, 11–24), expressed in years.



For peer review only - http://bmjopen.bmj.com/site/about/guidelines.xhtml

*Chemical co-exposures and main occupational activity in last position*

A research group at the University of Bergen has developed expert-based job-exposure matrices (JEMs) for the NOPW cohort to identify and describe the degree of exposure to agents, mixtures or exposure situations with known or suspected carcinogenic potential among offshore workers on the Norwegian continental shelf 1970–2005 (25, 35). The JEMs and their development have been described in detail elsewhere (25, 36). In brief, due to lack of exposure measurements, the assessment of the JEM for chlorinated degreasers was probability-oriented and based on summary documents (*e.g.* from company visits/interviews, risk assessment reports, sampling reports, product data sheets) on the exposure to tri- and tetrachloroethylene. Eight experts individually categorised the likelihood (unlikely, possible, probable) of chlorinated degreaser exposure (*i.e.* products containing tri- and tetrachloroethylene) for 27 job categories (35). Importantly, the exposure assessment of chlorinated degreasers was evaluated as "possible" for catering workers, meaning that some workers may have been exposed while the probability of exposure was low. For the benzene JEM, measurement data were used to construct a time-line of exposure and to rate exposure intensity of tasks specific to each job category. Then, task duration and frequency were combined to create a semi-quantitative benzene exposure burden score specific to job category and time period (1965–1969, 1970–1979, 1980–1989, 1990–1998) (36). For each of the two agents, we generated a dichotomized variable indicating never or ever exposure.

Main occupational activity in each worker's last position was recorded as production and process, drilling and well maintenance, maintenance/inspection/deck construction, catering/office/administration, and miscellaneous.

*Covariates*



For peer review only - http://bmjopen.bmj.com/site/about/guidelines.xhtml

Age at first child and number of children were recorded in the questionnaire, categorized as 0, 1, 2, ≥3 children. Females aged ≥53 years at baseline in 1998 were assumed postmenopausal, according to the convention from the Million Women Study (37) which also was adopted by the Norwegian Women and Cancer study (38) that is representative of the Norwegian female population. In supplemental analyses, we also used age ≥51 as cut-point to define postmenopause (Supplemental Material, Table S1, Table S2), which also has been used in previous study (39). Education was recorded as compulsory, vocational, folk high school, upper secondary (the two latter collapsed into upper secondary), and university/college.

**Data analysis**

Cox regression, adapted to a stratified case-cohort design (32), was used to estimate hazard ratios (HRs) of breast cancer and 95% confidence intervals (CIs) associated with work schedule, chlorinated degreasers and benzene. Cases were assigned a weight of 1 and subcohort non-cases were given weights according to the inverse sampling fraction in their corresponding 5-year birth cohort stratum. In the analyses, age was used as the time-scale with subjects entering start of follow-up on 30 June 1999. Subjects were censored at the date of any cancer diagnosis (except non-melanoma skin cancer), emigration, death, or end of study, whichever came first. The 22 cases identified as members of the randomly drawn subcohort were analysed as cases only (weight=1) (32). Robust variance was used to compute standard errors of the HRs. The proportional hazards assumption was evaluated by Schoenfeld residuals.

Based on the literature and the available variables in NOPW, a directed acyclic graph (DAG) was drawn to help model the relationship between work schedule and breast cancer risk (Figure S1, Supplemental Material). Based on the DAG, we adjusted for age at first child, number of children, menopausal status at baseline and education in addition to attained age



For peer review only - http://bmjopen.bmj.com/site/about/guidelines.xhtml

(timescale). Likewise, based on a DAG (Supplemental Material, Figure S2) we adjusted for age and education in the analyses of chlorinated degreasers and benzene in relation to breast cancer risk. We tested for linear trend by modelling ordinal variables as continuous variables. Tests of interaction between work schedule and chemical co-exposures, and between work schedule and menopausal status were conducted by including product terms of dichotomous variables in the models.

We had up to 39% missing in the covariates of the multivariable model. We used multiple imputation with chained equations, assuming missing at random (40), to impute 45 datasets. In each data set, we conducted analyses using the models described above, and estimates were combined using Rubin's rule (41). We report results with and without imputed data. We have extended the description of the imputations in the supplements where we also tried an alternative imputation model (Supplemental Material, Methods extension, Table S3).

Tests for significance were two-sided, and P-values <0.05 were considered as evidence against the null hypothesis. Data analyses were performed using Stata version 16.1 (StataCorp, College Station, TX, USA).

## RESULTS

Table 1 shows baseline characteristics of the 86 cases and 514 non-cases in the study sample. Mean age at baseline was 45 years for cases and 42 years for non-cases. Among the cases and non-cases, mean employment duration was 8.4 and 6.9 years, the most frequent main occupational activity in last position was catering/office/administration (73% *vs.* 71%, respectively), most had day work only (55% *vs.* 53%), and 37% of the cases *vs.* 42% of the non-cases were exposed to night/rollover shift. Twenty-seven percent of the cases had >6 years of night/rollover shift compared with 18% of non-cases. Up to 68% of the cases and 77% of the non-cases may have been exposed to chlorinated degreasers. For benzene, 15% of



For peer review only - http://bmjopen.bmj.com/site/about/guidelines.xhtml

the cases and 18% of the non-cases were exposed. Mean age at breast cancer diagnosis was 56 years, whereof 78% were ER-positive and 69% PR-positive, while 70% were HER2-negative (Supplemental Material, Table S4).

No interaction was found between work schedule and menopausal status when examining risk of overall or any breast cancer subtypes. The P-values of the interaction terms in the models were between 0.279 and 0.639, we therefore do not present results stratified by menopausal status. Results from complete-case and multiple imputation analyses were largely similar across all analyses (Tables 2-4), and we refer to the multiple imputation results in the following.

No association was found between breast cancer risk and total employment duration ($P_{trend}$=0.733) (Table 2). The HR of breast cancer in relation to work schedule ever involving night/rollover shift was 0.87 (95% CI: 0.52, 1.46) *vs.* never, and no significant association was found with duration of night/rollover shift ($P_{trend}$=0.748). Sensitivity analyses of duration of night/rollover shift by quartile among the exposed showed similar result ($P_{trend}$=0.944) (Supplemental Material, Table S5).

There was no significant association between main occupational activity in last position and breast cancer risk (Table 3). No significant associations with breast cancer risk were found for chlorinated degreaser or benzene exposure (HRs (95% CIs) 0.89 (0.52, 1.52) and 0.90 (0.46, 1.79), respectively, for ever *vs*. never, Table 3). No significant interaction was found between work schedule and chemical co-exposures ($P_{interaction\ chlorinated\ degreasers}$=0.725; $P_{interaction\ benzene}$=0.175) (Supplemental Material, Table S6).

There was no significant association between work schedule and risk of any breast cancer subtypes. For the breast cancer subtypes that did not express sex hormone receptors we found non-significantly elevated risks for work schedule involving night/rollover shift compared to day work only (HR=1.53, 95% CI: 0.50, 4.72 for ER-negative tumours;



For peer review only - http://bmjopen.bmj.com/site/about/guidelines.xhtml

HR=1.69, 95% CI: 0.58, 4.87 for PR-negative tumours; HR=2.22, 95% CI: 0.58, 8.51 for ER-negative and PR-negative and HER2-negative tumours) (Table 4).

## DISCUSSION

In this study of prospectively-recruited female offshore workers, we observed no association between overall breast cancer risk and night/rollover shift, duration of such work, or total employment duration. Some indication of increased risks was observed for ER-negative and PR-negative breast cancer subtypes, but these findings were not statistically significant and were based on few observations. We did not find evidence for interaction between work schedule and chemical co-exposures in relation to risk of breast cancer.

The literature is inconclusive on the association between night shift work and breast cancer risk. A report from the Nurses' Health Study (9) found an elevated risk among nurses who had long (≥20 years) cumulative night shift work and started their career early, which is also in accordance with other studies (10, 11, 17). One explanation for these positive associations between long-term night shift work and breast cancer risk may be that breast tissue is particularly susceptible to circadian disruption at a younger age (9). However, in line with our main findings, more recent studies did not find an association between night shift work and breast cancer (13-15). Importantly, the most recent meta-analysis, stratifying on short- and long-term night shift work, found an increased breast cancer risk related to short-term, but not long-term night shift work (42). Possible reasons that may explain our negative results are that, despite the extreme and unique work pattern, offshore workers have a four-week off-duty period following each standard two-week tour (6). During the off-duty period, it is possible that workers could rest and readjust their circadian rhythm, and hence reduce the potential effect of night shift work on breast cancer risk. Further, the continuous shift work pattern (7–14 consecutive nights) of offshore workers might facilitate some adaptation and



For peer review only - http://bmjopen.bmj.com/site/about/guidelines.xhtml

thereby represent a lower degree of circadian disruption, which in turn might reduce the risk of breast cancer (5).

To our knowledge, this is the first study of night shift work and co-exposure to chlorinated degreaser and benzene in relation to breast cancer risk. The hypothesis that night shift work may potentiate chemical exposure by desynchronising detoxification mechanisms (29) was not confirmed, as we did not find any statistically significant interactions, although it may be ascribed to lack of power and misclassification of exposure.

Some studies have investigated the relationship between night shift work and breast cancer subtypes. Night shift work has been associated with sex hormone receptor-positive (ER-positive or PR-positive) tumours (14, 17, 18, 20), which is in line with experimental evidence that melatonin suppression enhances proliferation of ER-positive cell lines (43). Zhu et al. (44) suggested that melatonin disturbance could instigate two independent pathways that lead to both ER-positive and ER-negative tumours. This would be in line with our findings of increased risk of ER-negative tumours among night-shift workers, which are consistent with the study by Rabstein et al. (22).

Our study has several strengths. First, linkage to nationwide cancer and population registries ensured high quality cancer data and complete follow-up. Second, we had access to independent chemical exposure estimates developed by industrial hygiene experts specifically for cancer studies in our cohort. Third, offshore workers have extreme work schedules and the NOPW cohort therefore provides particularly interesting data to study the association with breast cancer. Although, self-reported work history (*i.e.* exposure) was collected prior to the breast cancer diagnoses, and has been shown to be robust to recall errors (45), we cannot rule out some differential misclassification as a result of collapsing exposure categories (46, 47). However, the results of analysis with categorical and continuous exposure gave similar results (Table 2). The females in the NOPW cohort were quite young at baseline (mean age 42),



For peer review only - http://bmjopen.bmj.com/site/about/guidelines.xhtml

which may explain the relatively few cases. Also, the low case numbers in some exposure categories reduced the statistical power of detecting significant deviations from the null hypothesis. Comparing our results with the pooled-analysis of Cordina-Duverger *et al*. (17), which had larger sample size and reported a positive association between night shift work and breast cancer (all receptor subtypes combined), the two studies do not contradict each other (HR=0.87, 95% CI: 0.52, 1.46 in our study *vs.* OR=1.12, 95% CI: 1.00, 1.25 in Cordina-Duverger *et al.*). Our study had relatively few cases resulting in low power and wide confidence intervals. The limited power may leave real interactions undetected. In an attempt to reduce the impact of limited power, we tested interactions with binary variables (ever/never exposure). The probability-oriented JEM for chlorinated degreasers classified catering workers as 'possibly exposed' and assigned the same exposure level despite the fact that exposure may vary by job tasks. Hence, the actual fraction of workers exposed to chlorinated degreasers in this job-category is uncertain. We also lacked information on some potential confounders (*e.g.* chronotype, exogenous hormone use, family history and breast feeding history), and we had no information on exposures during follow-up.

In conclusion, our findings in this prospectively-recruited cohort need to be interpreted carefully due to limited statistical power, but pointed towards that night/rollover shift, exposure to chlorinated degreasers or to benzene may not be associated with increased breast cancer risk in female offshore petroleum workers. However, further studies with larger sample sizes are warranted for analyses with combined exposures and for analyses of breast cancer by receptor subtype.



For peer review only - http://bmjopen.bmj.com/site/about/guidelines.xhtml

**FUNDING**

This work was supported by the Research Council of Norway grant number 280537.

**CONFLICTS OF INTEREST**

None declared. JSS and TKG have received a grant from the Research Council of Norway (governmental agency) that awarded an industry-collaborative grant to the Cancer Registry of Norway (governmental agency) in 2019 to establish a cohort of offshore petroleum workers. A condition pertaining to industry-collaborative grants is that 20% (175,000 USD) of the grant was provided by the petroleum industry and 80% (700,000 USD) by the Research Council itself with the intention of joining forces for the common interest of occupational health among petroleum workers. The application process was governed by the Research Council without any involvement from the industry. The grant does not cover the PI's or any of the collaborators salary.

**AUTHOR CONTRIBUTIONS**

JSS and TKG conceived the study. FCL, MBV, KK, TER, RG, HDH, SOS, MB, JK, NR, QL, DTS, MCF, RB, NKS, TKG and JSS contributed to the project design. FCL performed the data analyses. FCL, RB, and JSS performed the data management. KK contributed with their expertise on breast cancer epidemiology. MB and JK contributed to chemical exposure assessment. FCL drafted the manuscript and all authors reviewed and revised it critically for important intellectual content and approved the final version for submission. FCL and JSS are the guarantors.



For peer review only - http://bmjopen.bmj.com/site/about/guidelines.xhtml

## ETHICS APPROVAL

This study was performed at the CRN after legal and ethical approvals were obtained from the Norwegian Data Inspectorate, the Regional Committee for Medical Research Ethics (2018/1162), and the Norwegian Directorate of Health.

## DATA SHARING STATEMENT

The data that support the findings of this study are available from the CRN (cohort data and cancer data) and the National Population Register (death and emigration data) but restrictions apply to the availability of these data, which were used under license for the current study, and so are not publicly available. Requests for data sharing/case pooling for projects with necessary approvals and legal basis according to the EU General Data Protection Regulation (GDPR) may be directed to principal investigator Dr Tom K Grimsrud; email: tom.k.grimsrud@kreftregisteret.no

For peer review only



For peer review only - http://bmjopen.bmj.com/site/about/guidelines.xhtml

# References

1. Bray F, Ferlay J, Soerjomataram I, Siegel RL, Torre LA, Jemal A. Global Cancer Statistics 2018: Globocan Estimates of Incidence and Mortality Worldwide for 36 Cancers in 185 Countries. CA Cancer J Clin. 2018;68(6):394-424.
2. Bray F, McCarron P, Parkin DM. The Changing Global Patterns of Female Breast Cancer Incidence and Mortality. Breast Cancer Res. 2004;6(6):229.
3. Francies FZ, Hull R, Khanyile R, Dlamini Z. Breast Cancer in Low-Middle Income Countries: Abnormality in Splicing and Lack of Targeted Treatment Options. Am J Cancer Res. 2020;10(5):1568-91.
4. Hansen J. Risk of Breast Cancer After Night- and Shift Work: Current Evidence and Ongoing Studies in Denmark. Cancer Causes & Control. 2006;17(4):531-7.
5. Stevens RG, Davis S. The Melatonin Hypothesis: Electric Power and Breast Cancer. Environ Health Persp. 1996;104(Suppl 1):135-40.
6. Parkes K. Working Hours in the Offshore Petroleum Industry Current Knowledge and Research Needs Regarding Extended Work Periods and Shift Work Offshore. In: Conference On Work Time Arrangements in the Petroleum Industry, 2007: Norway: Petroleum Safety Authority; 2007.
7. ILO. C171 - Night Work Convention No. 171 (C171). [Webpage]. Geneva, Switzerland: International Labour Organization: [cited 2020.29.09]. Available from: https://www.ilo.org/dyn/normlex/en/f?p=NORMLEXPUB:12100:0::NO::P12100_INSTRUMENT_ID:312316
8. ILO. R178 - Night Work Recommendation No.178 (R178). [Webpage]. Geneva, Switzerland: International Labour Organization: [cited 2020.29.09]. Available from: https://www.ilo.org/dyn/normlex/en/f?p=NORMLEXPUB:12100:0::NO::P12100_ILO_CODE:R178
9. Wegrzyn LR, Tamimi RM, Rosner BA, Brown SB, Stevens RG, Eliassen AH, et al. Rotating Night-Shift Work and the Risk of Breast Cancer in the Nurses' Health Studies. Am J Epidemiol. 2017;186(5):532-40.
10. Åkerstedt T, Knutsson A, Narusyte J, Svedberg P, Kecklund G, Alexanderson K. Night Work and Breast Cancer in Women: a Swedish Cohort Study. BMJ Open. 2015;5(4):e008127.
11. Hansen J, Lassen CF. Nested Case–Control Study of Night Shift Work and Breast Cancer Risk Among Women in the Danish Military. Occup Environ Med. 2012;69(8):551-6.
12. Lie JS, Kjuus H, Zienolddiny S, Haugen A, Stevens RG, Kjærheim K. Night Work and Breast Cancer Risk Among Norwegian Nurses: Assessment by Different Exposure Metrics. Am J Epidemiol. 2011;173(11):1272-9.
13. Jones ME, Schoemaker MJ, McFadden EV, Wright LB, Johns LE, Swerdlow AJ. Night Shift Work and Risk of Breast Cancer in Women: The Generations Study Cohort. Br J Cancer. 2019;121(2):172-9.
14. Vistisen HT, Garde AH, Frydenberg M, Christiansen P, Hansen AM, Hansen J, et al. Short-Term Effects of Night Shift Work on Breast Cancer Risk: A Cohort Study of Payroll Data. Scand J Work Environ Health. 2017;43(1):59-67.
15. Travis RC, Balkwill A, Fensom GK, Appleby PN, Reeves GK, Wang XS, et al. Night Shift Work and Breast Cancer Incidence: Three Prospective Studies and Meta-Analysis of Published Studies. J Natl Cancer Inst. 2016;108(12):djw169.
16. Koppes LL, Geuskens GA, Pronk A, Vermeulen RC, de Vroome EM. Night Work and Breast Cancer Risk in a General Population Prospective Cohort Study in the Netherlands. Eur J Epidemiol. 2014;29(8):577-84.
17. Cordina-Duverger E, Menegaux F, Popa A, Rabstein S, Harth V, Pesch B, et al. Night Shift Work and Breast Cancer: A Pooled Analysis of Population-Based Case–Control Studies With Complete Work History. Eur J Epidemiol. 2018;33(4):369-79.
18. Cordina-Duverger E, Koudou Y, Truong T, Arveux P, Kerbrat P, Menegaux F, et al. Night Work and Breast Cancer Risk Defined by Human Epidermal Growth Factor Receptor-2 (HER2) and Hormone Receptor Status: A Population-Based Case-Control Study in France. Chronobiol Int. 2016;33(6):783-7.



For peer review only - http://bmjopen.bmj.com/site/about/guidelines.xhtml

19. Schernhammer ES, Laden F, Speizer FE, Willett WC, Hunter DJ, Kawachi I, et al. Rotating Night Shifts and Risk of Breast Cancer in Women Participating in the Nurses' Health Study. J Natl Cancer Inst. 2001;93(20):1563-8.
20. Lie JS, Kjuus H, Zienolddiny S, Haugen A, Kjærheim K. Breast Cancer Among Nurses: Is the Intensity of Night Work Related to Hormone Receptor Status? Am J Epidemiol. 2013;178(1):110-7.
21. Papantoniou K, Castaño-Vinyals G, Espinosa A, Aragonés N, Pérez-Gómez B, Ardanaz E, et al. Breast Cancer Risk and Night Shift Work in a Case–Control Study in a Spanish Population. Eur J Epidemiol. 2016;31(9):867-78.
22. Rabstein S, Harth V, Pesch B, Pallapies D, Lotz A, Justenhoven C, et al. Night Work and Breast Cancer Estrogen Receptor Status--Results From the German Genica Study. Scand J Work Environ Health. 2013;39(5):448-55.
23. IARC. Night Shift Work. IARC Monogr Identif Carcinog Hazards Hum. 2020;124:1-371.
24. Bratveit M, Kirkeleit J, Hollund BE, Moen BE. Biological Monitoring of Benzene Exposure for Process Operators during Ordinary Activity in the Upstream Petroleum Industry. Ann Occup Hyg. 2007;51(5):487-94.
25. Steinsvåg K, Bratveit M, Moen BE. Exposure to Carcinogens for Defined Job Categories in Norway's Offshore Petroleum Industry, 1970 to 2005. Occup Environ Med. 2007;64(4):250-8.
26. Garcia E, Hurley S, Nelson DO, Hertz A, Reynolds P. Hazardous Air Pollutants and Breast Cancer Risk in California Teachers: A Cohort Study. Environmental health : a global access science source. 2015;14(1):14.
27. Pedersen JE, Strandberg-Larsen K, Andersson M, Hansen J. Occupational Exposure to Specific Organic Solvents and Risk of Subtypes of Breast Cancer in a Large Population of Danish Women, 1964-2016. Occup Environ Med. 2020;oemed-2020-106865. doi: 10.1136/oemed-2020-106865. Online ahead of print.
28. Masri S, Sassone-Corsi P. The Emerging Link Between Cancer, Metabolism, and Circadian Rhythms. Nat Med 2018;24:1795-803.
29. Carmona-Antoñanzas G, Santi M, Migaud H, Vera LM. Light- and Clock-Control of Genes Involved in Detoxification. Chronobiol Int. 2017;34(8):1026-41.
30. Stenehjem JS, Babigumira R, Hosgood HD, Veierød MB, Samuelsen SO, Bråtveit M, et al. Cohort Profile: The Norwegian Offshore Petroleum Workers (NOPW) Cohort. Int J Epidemiol. 2020;dyaa107.
31. Stenehjem JS, Kjaerheim K, Rabanal KS, Grimsrud TK. Cancer Incidence Among 41,000 Offshore Oil Industry Workers. Occup Med (Lond). 2014;64(7):539-45.
32. Borgan O, Langholz B, Samuelsen SO, Goldstein L, Pogoda J. Exposure Stratified Case-Cohort Designs. Lifetime Data Anal. 2000;6(1):39-58.
33. Larsen IK, Smastuen M, Johannesen TB, Langmark F, Parkin DM, Bray F, et al. Data Quality at the Cancer Registry of Norway: An Overview of Comparability, Completeness, Validity and Timeliness. Eur J Cancer. 2009;45(7):1218-31.
34. Stenehjem JS, Babigumira R, Friesen MC, Grimsrud TK. Harmonizing Work History Data in Epidemiologic Studies With Overlapping Employment Records. Am J Ind Med. 2019;62(5):422-9.
35. Steinsvag K, Bratveit M, Moen BE, Kromhout H. Inter-Rater Agreement in the Assessment of Exposure to Carcinogens in the Offshore Petroleum Industry. Occup Environ Med. 2007;64(9):582-8.
36. Bråtveit M, Kirkeleit J, Hollund BE, Vågnes KS. Development of a Retrospective JEM for Benzene in the Norwegian Oil and Gas Industry. Occup Environ Med. 2011;68(Suppl 1):A26-A.
37. Beral V. Breast Cancer and Hormone-Replacement Therapy in the Million Women Study. Lancet. 2003;362(9382):419-27.
38. Busund M, Bugge NS, Braaten T, Waaseth M, Rylander C, Lund E. Progestin-Only and Combined Oral Contraceptives and Receptor-Defined Premenopausal Breast Cancer Risk: The Norwegian Women and Cancer Study. Int J Cancer. 2018;142(11):2293-302.
39. Bjelland EK, Hofvind S, Byberg L, Eskild A. The Relation of Age at Menarche with Age at Natural Menopause: A Population Study of 336 788 Women in Norway. Hum Reprod. 2018;33(6):1149-57.



For peer review only - http://bmjopen.bmj.com/site/about/guidelines.xhtml

40. Bartlett JW, Seaman SR, White IR, Carpenter JR. Multiple Imputation of Covariates by Fully Conditional Specification: Accommodating the Substantive Model. Stat Methods Med Res. 2015;24(4):462-87.
41. Rubin DB. Multiple Imputation for Nonresponse in Surveys. Hoboken, New Jersey: John Wiley & Sons; 2004. 131 p.
42. Manouchehri E, Taghipour A, Ghavami V, Ebadi A, Homaei F, Latifnejad Roudsari R. Night-Shift Work Duration and Breast Cancer Risk: An Updated Systematic Review and Meta-Analysis. BMC women's health. 2021;21(1):89.
43. Girgert R, Bartsch C, Hill SM, Kreienberg R, Hanf V. Tracking the Elusive Antiestrogenic Effect of Melatonin: A New Methodological Approach. Neuro Endocrinol Lett. 2003;24(6):440-4.
44. Zhu K, Bernard LJ, Levine RS, Williams SM. Estrogen Receptor Status of Breast Cancer: A Marker of Different Stages of Tumor or Different Entities of the Disease? Med Hypotheses. 1997;49(1):69-75.
45. Wärneryd B, Thorslund M, Ostlin P. The Quality of Retrospective Questions About Occupational History-a Comparison Between Survey and Census Data. Scand J Soc Med. 1991;19(1):7-13.
46. Wacholder S, Dosemeci M, Lubin JH. Blind Assignment of Exposure Does Not Always Prevent Differential Misclassification. Am J Epidemiol. 1991;134(4):433-7.
47. Jurek AM, Greenland S, Maldonado G, Church TR. Proper Interpretation of Non-Differential Misclassification Effects: Expectations vs Observations. Int J Epidemiol. 2005;34(3):680-7.

For peer review only



For peer review only - http://bmjopen.bmj.com/site/about/guidelines.xhtml

# TABLES

| Table 1. Baseline Characteristics of Females in the Norwegian Offshore Petroleum Workers (NOPW) Cohort. | | |
|---|---|---|
| **Variables** | **Cases (n=86)** | **Non-cases (n= 514)** |
| Age at baseline in 1998 (years), mean (range) | 45 (25-65) | 42 (19-74) |
| | | |
| **Anthropometric factors** | | |
| Height (cm), mean (range)[a] | 167 (155-184) | 167 (151-187) |
| Weight (kg), mean (range)[a] | 66 (49-95) | 66 (43-163) |
| BMI (kg/m$^2$), n (%) | | |
| 12-18.4 | 1 (1) | 13 (2) |
| 18.5-24.9 | 63 (73) | 343 (67) |
| 25.0-29.9 | 15 (18) | 121 (24) |
| ≥30.0 | 5 (6) | 29 (6) |
| Missing | 2 (2) | 8 (1) |
| | | |
| **Reproductive history** | | |
| Number of children, n (%) | | |
| 0 | 29 (34) | 151 (29) |
| 1 | 24 (28) | 85 (16) |
| 2 | 13 (15) | 147 (29) |
| ≥3 | 20 (23) | 123 (24) |
| Missing | 0 (0) | 8 (2) |
| Age at first child, mean (range)[a] | 26 (16-38) | 24 (16-41) |
| Postmenopausal at baseline, n (%)[b] | 18 (21) | 111 (22) |
| | | |
| **Social-demographic history** | | |
| Education, n (%) | | |
| Compulsory | 18 (21) | 106 (21) |
| Vocational | 17 (20) | 118 (23) |
| Upper secondary | 23 (27) | 148 (29) |
| University/college | 24 (27) | 138 (26) |
| Missing | 4 (5) | 4 (1) |
| | | |
| **Work history** | | |
| Main occupational activity in last position, n (%) | | |
| Production and process | 2 (2) | 23 (4) |
| Drilling and well maintenance | 3 (3) | 22 (4) |
| Maintenance/inspection/deck construction | 9 (11) | 59 (12) |
| Catering/office/administration | 63 (73) | 364 (71) |
| Miscellaneous | 9 (11) | 39 (8) |
| Missing | 0 (0) | 7 (1) |
| Total employment duration (years), mean (range) | 8.4 (0.3-20) | 6.9 (0.1-24) |
| Total employment duration quartile, n (%) | | |
| Quartile 1 (0-1.9 years) | 13 (15) | 124 (24) |
| Quartile 2 (2-5.9 years) | 23 (27) | 146 (28) |
| Quartile 3 (6-10.9 years) | 26 (30) | 121 (24) |
| Quartile 4 (11-24 years) | 24 (28) | 123 (24) |
| Work schedule, n (%) | | |
| Day work only | 47 (55) | 275 (53) |
| Mixed I (some night/rollover + mainly day) | 1 (1) | 18 (4) |
| Mixed II (mainly night/rollover + some day) | 31 (36) | 196 (38) |
| Missing | 7 (8) | 25 (5) |
| Duration by work schedule [a,c] | | |
| Day work (years), mean (range) | 6.4 (0.3-20) | 5.7 (0.1-21) |
| Night work (years), mean (range) | 3.6 (2.0-5.3) | 2.0 (0.1-11) |
| Rollover shift (years), mean (range) | 9.3 (1.0-18) | 6.8 (0.1-22) |
| Duration of night/rollover shift, n (%) | | |
| Unexposed (0 years, day work only) | 47 (55) | 275 (53) |
| ≤Median night/rollover (<1-6 years) [d] | 9 (10) | 121 (24) |
| >Median night/rollover (>6 years) [d] | 23 (27) | 93 (18) |
| Missing | 7 (8) | 25 (5) |
| | | |
| **Chemical co-exposure** | | |
| Chlorinated degreasers duration, n (%) [e] | | |
| Unexposed (0 years) | 28 (32) | 121 (23) |



For peer review only - http://bmjopen.bmj.com/site/about/guidelines.xhtml

| | | |
|---|---|---|
| ≤Median (<1–5 years) | 22 (26) | 214 (42) |
| >Median (6–23 years) | 36 (42) | 179 (35) |
| Benzene duration, n (%) | | |
| Unexposed (0 years) | 73 (85) | 424 (82) |
| Exposed (<1–23 years) | 13 (15) | 90 (18) |

[a] Missing in continuous variables: Height (n=8); Weight (n=9); Age at first child (n=193); Day work duration (n=32); Night work duration (n=32); Rollover shift duration (n=32); Night + Rollover shift duration (n=32)

[b] Postmenopause assumed at ≥ 53 years of age

[c] Workers with complete missing information on day, night and rollover (n=32) were excluded when calculating the mean

[d] Workers with day work mainly but also worked night/rollover shift were included in this category

[e] Importantly, the exposure assessment of chlorinated degreasers was probability-based by experts and evaluated as "possible" for catering workers.

For peer review only



For peer review only - http://bmjopen.bmj.com/site/about/guidelines.xhtml

| **Table 2**. Hazard Ratios of Female Breast Cancer According to Work Schedule in the Norwegian Offshore Petroleum Workers (NOPW) Cohort | | | | |
|---|---|---|---|---|
| | **Complete case analysis (n=367)** | | | **Multiple imputation (n=600; 86 cases)** |
| **Work schedule variable** | **No. of participants** | **No. of cases** | **HR (95% CI)** | **HR (95% CI)** |
| Total employment duration [a,b] | | | | |
| Quartile 1 (0-1.9 years) | 79 | 9 | 1.00 (reference) | 1.00 (reference) |
| Quartile 2 (2-5.9 years) | 99 | 16 | 1.59 (0.58, 4.35) | 1.50 (0.69, 3.27) |
| Quartile 3 (6-10.9 years) | 90 | 8 | 0.68 (0.24, 1.94) | 1.64 (0.78, 3.50) |
| Quartile 4 (11-24 years) | 99 | 16 | 1.21 (0.46, 3.13) | 1.19 (0.54, 2.61) |
| P-trend [c1] | | | 0.864 | 0.733 |
| Work schedule involving night/rollover shift [a,b] | | | | |
| Unexposed (day work only) | 217 | 29 | 1.00 (reference) | 1.00 (reference) |
| Exposed (night/rollover shift) | 150 | 20 | 1.06 (0.57, 1.97) | 0.87 (0.52, 1.46) |
| | | | | |
| Duration of night/rollover shift [a,b] | | | | |
| Unexposed (0 years, day work only) | 217 | 29 | 1.00 (reference) | 1.00 (reference) |
| ≤Median night/rollover (<1-6 years) | 68 | 6 | 0.72 (0.28, 1.86) | 0.56 (0.26, 1.22) |
| >Median night/rollover (>6 years) | 82 | 14 | 1.34 (0.67, 2.72) | 1.21 (0.67, 2.18) |
| P-trend [c2] | | | 0.523 | 0.748 |
| Abbreviations: No=number, HR=hazard ratio, CI=confidence interval | | | | |
| [a] Adjusted for age, age at first child, number of children, menopause status, and education | | | | |
| [b] complete work history i.e. up to 8 employments as an offshore worker | | | | |
| [c1] Modelled as a continuous variable to test for linear trend, HR-complete case 0.97 (95% CI: 0.72, 1.31) and HR-imputed 1.04 (95% CI: 0.83, 1.30). | | | | |
| [c2] Modelled as a continuous variable to test for linear trend, HR-complete case 1.13 (95% CI: 0.78, 1.63) and HR-imputed 1.05 (95% CI: 0.77, 1.44). | | | | |

For peer review only



For peer review only - http://bmjopen.bmj.com/site/about/guidelines.xhtml

**Table 3**. Hazard Ratios of Female Breast Cancer According to Chemical Co-Exposure in the Norwegian Offshore Petroleum Workers (NOPW) Cohort

| | Complete case analysis (n=367) | | | Multiple imputation (n=600; 86 cases) |
|---|---|---|---|---|
| **Chemical exposure variable** | **No. of participants** | **No. of cases** | **HR (95% CI)** | **HR (95% CI)** |
| Main occupational activity in last position [a] | | | | |
| Production/drilling/maintenance | 58 | 8 | 1.17 (0.47, 2.88) | 0.92 (0.48, 1.79) |
| Catering/office/administration | 280 | 35 | 1.00 (reference) | 1.00 (reference) |
| Miscellaneous | 29 | 6 | 1.70 (0.63, 4.53) | 1.19 (0.54, 2.63) |
| | | | | |
| Chlorinated degreasers [a,b] | | | | |
| Never | 95 | 17 | 1.00 (reference) | 1.00 (reference) |
| Ever | 272 | 32 | 0.72 (0.35, 1.48) | 0.89 (0.52, 1.52) |
| | | | | |
| Benzene [a] | | | | |
| Never | 309 | 40 | 1.00 (reference) | 1.00 (reference) |
| Ever | 58 | 9 | 1.54 (0.65, 3.64) | 0.90 (0.46, 1.79) |

Abbreviations: No=number, HR=hazard ratio, CI=confidence interval

[a] Adjusted for age and education

[b] Importantly, the exposure assessment of chlorinated degreasers was probability-based by experts and evaluated as “possible” for catering workers.

For peer review only

**Table 4** Hazard Ratios of Female Breast Cancer by Receptor Status Subtypes in Relation to Work Schedule in the Norwegian Offshore Petroleum Workers (NOPW) Cohort

| | | Complete case analysis (n=367) | | | Multiple imputation (n=600; 86 cases) |
|---|---|---|---|---|---|
| **Breast cancer subtype** | **Work schedule variable** | **No. of participants** | **No. of cases** | **HR (95% CI)** | **HR (95% CI)** |
| ER-positive | Work schedule involving night/rollover shift [a] | | | | |
| | Unexposed (day work only) | 217 | 23 | 1.00 (reference) | 1.00 (reference) |
| | Exposed (night/rollover shift) | 150 | 16 | 1.00 (0.50, 2.00) | 0.75 (0.41, 1.36) |
| ER-negative | Work schedule involving night/rollover shift [a] | | | | |
| | Unexposed (day work only) | 217 | 5 | 1.00 (reference) | 1.00 (reference) |
| | Exposed (night/rollover shift) | 150 | 2 | 0.78 (0.14, 4.46) | 1.53 (0.50, 4.72) |
| PR-positive | Work schedule involving night/rollover shift [a] | | | | |
| | Unexposed (day work only) | 217 | 25 | 1.00 (reference) | 1.00 (reference) |
| | Exposed (night/rollover shift) | 150 | 13 | 0.80 (0.39, 1.68) | 0.67 (0.36, 1.26) |
| PR-negative | Work schedule involving night/rollover shift [a] | | | | |
| | Unexposed (day work only) | 217 | 3 | 1.00 (reference) | 1.00 (reference) |
| | Exposed (night/rollover shift) | 150 | 5 | 2.10 (0.55, 8.08) | 1.69 (0.58, 4.87) |
| HER2-positive | Work schedule involving night/rollover shift [a] | | | | |
| | Unexposed (day work only) | 217 | 7 | 1.00 (reference) | 1.00 (reference) |
| | Exposed (night/rollover shift) | 150 | 1 | 0.29 (0.04, 2.14) | 0.22 (0.05, 0.99) |
| HER2-negative | Work schedule involving night/rollover shift [a] | | | | |
| | Unexposed (day work only) | 217 | 21 | 1.00 (reference) | 1.00 (reference) |
| | Exposed (night/rollover shift) | 150 | 14 | 1.02 (0.49, 2.15) | 0.88 (0.47, 1.62) |
| ER-positive and PR-positive and HER2-negative | Work schedule involving night/rollover shift [a] | | | | |
| | Unexposed (day work only) | 217 | 17 | 1.00 (reference) | 1.00 (reference) |
| | Exposed (night/rollover shift) | 150 | 9 | 0.79 (0.32, 1.95) | 0.58 (0.26, 1.28) |
| ER-positive and PR-positive and HER2-positive | Work schedule involving night/rollover shift [a] | | | | |
| | Unexposed (day work only) | 217 | 5 | 1.00 (reference) | 1.00 (reference) |
| | Exposed (night/rollover shift) | 150 | 1 | 0.38 (0.05, 2.70) | 0.29 (0.06, 1.29) |
| ER-negative and PR-negative and HER2-positive | Work schedule involving night/rollover shift [a] | | | | |
| | Unexposed (day work only) | 217 | 0 | 1.00 (reference) | 1.00 (reference) |
| | Exposed (night/rollover shift) | 150 | 0 | - | - |
| ER-negative and PR-negative and HER2-negative | Work schedule involving night/rollover shift [a] | | | | |
| | Unexposed (day work only) | 217 | 3 | 1.00 (reference) | 1.00 (reference) |
| | Exposed (night/rollover shift) | 150 | 2 | 1.36 (0.16, 11) | 2.22 (0.58, 8.51) |

Abbreviations: No=number, HR=hazard ratio, CI=confidence interval, ER=oestrogen receptor, PR=progesterone receptor, HER2=human epidermal growth factor receptor 2

[a] Adjusted for age, age at first child, number of children, menopause status, and education



For peer review only - http://bmjopen.bmj.com/site/about/guidelines.xhtml

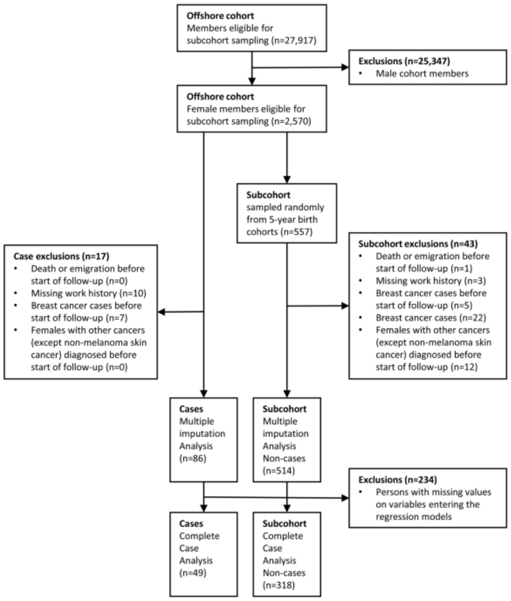


Figure 1. Overview of study design and exclusions.

275x190mm (300 x 300 DPI)

**SUPPLEMENTAL MATERIAL**

**Content:**

- Methods' extension on multiple imputations
- Table S1
- Table S2
- Table S3
- Table S4
- Table S5
- Table S6
- Directed acyclic graphs (DAGs)
    - Figure S1
    - Figure S2

For peer review only



For peer review only - http://bmjopen.bmj.com/site/about/guidelines.xhtml

**Methods' extension**

<u>Multiple imputations</u>

*Complete case analysis (n=367)*

As shown in Figure 1, the study sample for our complete case analysis included 49 breast cancer cases (with complete information on all variables in the regression model).

*Analysis of imputed data (n=600)*

As shown in Figure 1, our study sample yielded 86 breast cancer cases when those missing data retained. In this sample, 39% (n=233) had missing data on one or more of the following variables (missing numbers are displayed per variable):

- Work schedule involving night/rollover shift: n=32
- Duration of night/rollover shift: n=32
- Number of children: n=8
- Age at first child: n=192
- Education: n=8
- Main occupational activity in last position: n=7

Assuming missing at random, we used multiple imputation with chained equations and followed a 2-steps procedure:

1. We identified differences between these samples for date of birth, total employment duration, and breast cancer diagnosis, and used these variables as predictors together with the variables from the estimation model (i.e. work schedule involving night/rollover shift, duration of night/rollover shift, number of children, age at first child, education, and main



For peer review only - http://bmjopen.bmj.com/site/about/guidelines.xhtml

occupational activity in last position) to simultaneously predict missing data on all variables. Linear and multinomial logit models were used to predict missing data for continuous and categorical variables, respectively. The imputation model was ran 45 times, using the rule of thumb of running at least as many times as the percentage of missing data (White et al., 2011).

2. After step 1, we replicated the complete case analyses shown in Table 2, Table 3, and Table 4 on the imputed dataset (n=600).

In a sensitivity analysis (Table S4), we also tried an alternative imputation model where we excluded the variable ‘age at first child’, which had the highest number of missing (n=192, 39%). This reduced the total number of missing to n=53 (9%), but did not changed the results materially (Table S4).

Reference

White IR, Royston P, Wood AM. Multiple imputation using chained equations: Issues and guidance for practice. Statistics in medicine 2011; 30: 377-99.

For peer review only



For peer review only - http://bmjopen.bmj.com/site/about/guidelines.xhtml

# Table S1

| **Table S1**. Hazard Ratios of Female Breast Cancer According to Work Schedule in the Norwegian Offshore Petroleum Workers (NOPW) Cohort, sensitivity analyses with menopause cut-off at age ≥51 | | | | |
|---|---|---|---|---|
| | **Complete case analysis (n=367)** | | | **Multiple imputation (n=600; 86 cases)** |
| **Work schedule variable** | **No. of participants** | **No. of cases** | **HR (95% CI)** | **HR (95% CI)** |
| Total employment duration [a,b] | | | | |
| Quartile 1 (0-1.9 years) | 79 | 9 | 1.00 (reference) | 1.00 (reference) |
| Quartile 2 (2-5.9 years) | 99 | 16 | 1.62 (0.59, 4.42) | 1.48 (0.68, 3.23) |
| Quartile 3 (6-10.9 years) | 90 | 8 | 0.69 (0.24, 2.00) | 1.61 (0.77, 3.38) |
| Quartile 4 (11-24 years) | 99 | 16 | 1.19 (0.46, 3.04) | 1.17 (0.54, 2.55) |
| P-trend [c] | | | 0.834 | 0.765 |
| Work schedule involving night/rollover shift [a,b] | | | | |
| Unexposed (day work only) | 217 | 29 | 1.00 (reference) | 1.00 (reference) |
| Exposed (night/rollover shift) | 150 | 20 | 1.05 (0.57, 1.96) | 0.86 (0.51, 1.45) |
| | | | | |
| Duration of night/rollover shift [a,b] | | | | |
| Unexposed (0 years, day work only) | 217 | 29 | 1.00 (reference) | 1.00 (reference) |
| ≤Median night/rollover (<1-6 years) | 68 | 6 | 0.71 (0.27, 1.86) | 0.55 (0.25, 1.21) |
| >Median night/rollover (>6 years) | 82 | 14 | 1.33 (0.66, 2.68) | 1.21 (0.67, 2.18) |
| P-trend [c] | | | 0.543 | 0.753 |
| Abbreviations: No=number, HR=hazard ratio, CI=confidence interval | | | | |
| [a] Adjusted for age, age at first child, number of children, menopause status (≥51 years as cut-off), and education | | | | |
| [b] complete work history i.e. up to 8 employments as an offshore worker | | | | |
| [c] Modelled as a continuous variable to test for linear trend. | | | | |

For peer review only

# Table S2

**Table S2.** Hazard Ratios of Female Breast Cancer by Receptor Status Subtypes in Relation to Work Schedule in the Norwegian Offshore Petroleum Workers (NOPW) Cohort, sensitivity analyses with menopause cut-off at age ≥51

| | | Complete case analysis (n=367) | | | Multiple imputation (n=600; 86 cases) |
|---|---|---|---|---|---|
| **Breast cancer subtype** | **Work schedule variable** | **No. of participants** | **No. of cases** | **HR (95% CI)** | **HR (95% CI)** |
| ER-positive | Work schedule involving night/rollover shift [a] | | | | |
| | Unexposed (day work only) | 217 | 23 | 1.00 (reference) | 1.00 (reference) |
| | Exposed (night/rollover shift) | 150 | 16 | 1.00 (0.50, 2.01) | 0.74 (0.41, 1.34) |
| ER-negative | Work schedule involving night/rollover shift [a] | | | | |
| | Unexposed (day work only) | 217 | 5 | 1.00 (reference) | 1.00 (reference) |
| | Exposed (night/rollover shift) | 150 | 2 | 0.76 (0.14, 4.17) | 1.53 (0.50, 4.70) |
| PR-positive | Work schedule involving night/rollover shift [a] | | | | |
| | Unexposed (day work only) | 217 | 25 | 1.00 (reference) | 1.00 (reference) |
| | Exposed (night/rollover shift) | 150 | 13 | 0.79 (0.38, 1.64) | 0.66 (0.35, 1.24) |
| PR-negative | Work schedule involving night/rollover shift [a] | | | | |
| | Unexposed (day work only) | 217 | 3 | 1.00 (reference) | 1.00 (reference) |
| | Exposed (night/rollover shift) | 150 | 5 | 2.08 (0.55, 7.88) | 1.72 (0.59, 5.02) |
| HER2-positive | Work schedule involving night/rollover shift [a] | | | | |
| | Unexposed (day work only) | 217 | 7 | 1.00 (reference) | 1.00 (reference) |
| | Exposed (night/rollover shift) | 150 | 1 | 0.27 (0.04, 1.91) | 0.21 (0.05, 0.98) |
| HER2-negative | Work schedule involving night/rollover shift [a] | | | | |
| | Unexposed (day work only) | 217 | 21 | 1.00 (reference) | 1.00 (reference) |
| | Exposed (night/rollover shift) | 150 | 14 | 1.04 (0.50, 2.16) | 0.88 (0.48, 1.62) |
| ER-positive and PR-positive and HER2-negative | Work schedule involving night/rollover shift [a] | | | | |
| | Unexposed (day work only) | 217 | 17 | 1.00 (reference) | 1.00 (reference) |
| | Exposed (night/rollover shift) | 150 | 9 | 0.80 (0.33, 1.94) | 0.58 (0.26, 1.28) |
| ER-positive and PR-positive and HER2-positive | Work schedule involving night/rollover shift [a] | | | | |
| | Unexposed (day work only) | 217 | 5 | 1.00 (reference) | 1.00 (reference) |
| | Exposed (night/rollover shift) | 150 | 1 | 0.35 (0.05, 2.36) | 0.27 (0.06, 1.26) |
| ER-negative and PR-negative and HER2-positive | Work schedule involving night/rollover shift [a] | | | | |
| | Unexposed (day work only) | 217 | 0 | 1.00 (reference) | 1.00 (reference) |
| | Exposed (night/rollover shift) | 150 | 0 | - | - |
| ER-negative and PR-negative and HER2-negative | Work schedule involving night/rollover shift [a] | | | | |
| | Unexposed (day work only) | 217 | 3 | 1.00 (reference) | 1.00 (reference) |
| | Exposed (night/rollover shift) | 150 | 2 | 1.34 (0.17, 11) | 2.22 (0.58, 8.45) |

Abbreviations: No=number, HR=hazard ratio, CI=confidence interval, ER=oestrogen receptor, PR=progesterone receptor, HER2=human epidermal growth factor receptor 2

[a] Adjusted for age, age at first child, number of children, menopause status (≥51 years as cut-off), and education



For peer review only - http://bmjopen.bmj.com/site/about/guidelines.xhtml

# Table S3

| **Table S3**. Hazard Ratios of Female Breast Cancer According to Work Schedule in the Norwegian Offshore Petroleum Workers (NOPW) Cohort. Sensitivity Analyses of models adjusted for age at first child *versus* models without adjustment for age at first child. | | |
|---|---|---|
| | **Multiple imputation, including 'age at first child' (n=600; 86 cases) [a,b]** | **Multiple imputation, excluding 'age at first child' (n=600; 86 cases) [b,c]** |
| **Work schedule variable** | **HR (95% CI)** | **HR (95% CI)** |
| Total employment duration | | |
| Quartile 1 (0-1.9 years) | 1.00 (reference) | 1.00 (reference) |
| Quartile 2 (2-5.9 years) | 1.50 (0.69, 3.27) | 1.53 (0.71, 3.31) |
| Quartile 3 (6-10.9 years) | 1.64 (0.78, 3.50) | 1.65 (0.78, 3.47) |
| Quartile 4 (11-24 years) | 1.19 (0.54, 2.61) | 1.22 (0.56, 2.67) |
| P-trend [d1] | 0.733 | 0.685 |
| Work schedule involving night/rollover shift | | |
| Unexposed (day work only) | 1.00 (reference) | 1.00 (reference) |
| Exposed (night/rollover shift) | 0.87 (0.52, 1.46) | 0.87 (0.52, 1.45) |
| | | |
| Duration of night/rollover shift | | |
| Unexposed (0 years, day work only) | 1.00 (reference) | 1.00 (reference) |
| ≤Median night/rollover (<1-6 years) | 0.56 (0.26, 1.22) | 0.55 (0.25, 1.19) |
| >Median night/rollover (>6 years) | 1.21 (0.67, 2.18) | 1.21 (0.67, 2.17) |
| P-trend [d2] | 0.748 | 0.752 |
| Abbreviations: HR=hazard ratio, CI=confidence interval | | |
| [a] Adjusted for age, age at first child, number of children, menopause status (≥53 years as cut-off), and education. 39% (n=233) missing data imputed. | | |
| [b] complete work history i.e. up to 8 employments as an offshore worker | | |
| [c] Adjusted for variable in footnote "a" but without age at first child. 9% (n=53) missing data imputed. | | |
| [d1] Modelled as a continuous variable to test for linear trend. HR for excluding 'age at first child' 1.05 (95% CI: 0.84-1.31). | | |
| [d2] Modelled as a continuous variable to test for linear trend. HR for excluding 'age at first child' 1.05 (95% CI: 0.77-1.44). | | |

For peer review only

# Table S4

| **Table S4.** Characteristics of Female Breast Cancer Cases in the Norwegian Offshore Petroleum Workers (NOPW) Cohort. | |
|---|---|
| | **Breast Cancer Cases [a] (n=86)** |
| Age at diagnosis, mean (range) | 56 (38-80) |
| Clinical stage, n (%) | |
| I: No metastasis | 38 (44) |
| II: Regional lymph node metastasis | 29 (34) |
| III: Local infiltration into skin | 12 (14) |
| IV: Distant metastasis | 4 (5) |
| Unspecified | 3 (3) |
| Oestrogen receptors (ER), n (%) | |
| ER-positive | 67 (78) |
| ER-negative | 13 (15) |
| Missing | 6 (7) |
| Progesterone receptors (PR), n (%) | |
| PR-positive | 59 (69) |
| PR-negative | 21 (24) |
| Missing | 6 (7) |
| Human epidermal growth factor receptor-2 (HER2), n (%) | |
| HER2-positive | 12 (14) |
| HER2-negative | 60 (70) |
| Missing | 14 (16) |
| ER-positive and PR-positive and HER2-negative, n (%) | |
| Yes | 39 (45) |
| No | 30 (35) |
| Missing | 17 (20) |
| ER-positive and PR-positive and HER2-positive, n (%) | |
| Yes | 9 (10) |
| No | 60 (70) |
| Missing | 17 (20) |
| ER-negative and PR-negative and HER2-positive, n (%) | |
| Yes | 0 (0) |
| No | 69 (80) |
| Missing | 17 (20) |
| ER-negative and PR-negative and HER2-negative | |
| Yes | 11 (13) |
| No | 58 (67) |
| Missing | 17 (20) |
| [a] According to International Classification of Diseases 10th edition : C50 | |

For peer review only



For peer review only - http://bmjopen.bmj.com/site/about/guidelines.xhtml

# Table S5

| **Table S5**. Hazard Ratios of Breast Cancer According to duration of night + rollover shift quartile in the Norwegian Offshore Petroleum Workers (NOPW) Cohort | | | | |
|---|---|---|---|---|
| | **Complete case analysis (n=367)** | | | **Multiple imputation (n=600; 86 cases)** |
| **Work schedule variable** | **No. of participants** | **No. of cases** | **HR (95% CI)** | **HR (95% CI)** |
| Duration of night/rollover shift [a,b] | | | | |
| Unexposed (0 years, day work only) | 217 | 29 | 1.00 (reference) | 1.00 (reference) |
| Quartile 1 night/rollover (<1-2 years) | 28 | 3 | 0.99 (0.28, 3.52) | 0.78 (0.28, 2.14) |
| Quartile 2 night/rollover (>2-5 years) | 32 | 2 | 0.52 (0.11, 2.47) | 0.49 (0.14, 1.71) |
| Quartile 3 night/rollover (>5-10 years) | 36 | 4 | 0.77 (0.24, 2.48) | 1.02 (0.47, 2.24) |
| Quartile 4 night/rollover (>10 years) | 54 | 11 | 1.62 (0.73, 3.59) | 1.10 (0.51, 2.37) |
| P-trend [c] | | | 0.506 | 0.944 |
| Abbreviations: No=number, HR=hazard ratio, CI=confidence interval | | | | |
| [a] Adjusted for age, age at first child, number of children, menopause status (≥53 years as cut-off), and education | | | | |
| [b] complete work history i.e. up to 8 employments as an offshore worker | | | | |
| [c] Modelled as a continuous variable to test for linear trend | | | | |



For peer review only - http://bmjopen.bmj.com/site/about/guidelines.xhtml

# Table S6

| **Table S6**. Hazard Ratios of Breast Cancer According to Work Schedule and Chemical Co-exposures in the Norwegian Offshore Petroleum Workers (NOPW) Cohort, multiple imputation analysis (n=601) | | | |
|---|---|---|---|
| | **Chlorinated degreasers [a]** | | |
| **Work schedule variable [b,d]** | **Unexposed** | **Exposed** | **P Value** |
| Unexposed (day work only) | | | |
| n (%) | 99 (17) | 223 (39) | |
| HR [c] (95% CI) | 1.00 (reference) | 0.91 (0.45, 1.84) | |
| Exposed (night/rollover shift) | | | |
| n (%) | 43 (8) | 204 (36) | |
| HR [c] (95% CI) | 1.01 (0.40, 2.55) | 0.79 (0.37, 1.71) | |
| $P_{interaction}$ | | | 0.725 |
| | **Benzene** | | |
| **Work schedule variable [b,d]** | **Unexposed** | **Exposed** | **P Value** |
| Unexposed (day work only) | | | |
| n (%) | 269 (48) | 53 (9) | |
| HR [c] (95% CI) | 1.00 (reference) | 0.60 (0.21, 1.70) | |
| Exposed (night/rollover shift) | | | |
| n (%) | 199 (35) | 48 (8) | |
| HR [c] (95% CI) | 0.74 (0.41, 1.32) | 1.11 (0.46, 2.70) | |
| $P_{interaction}$ | | | 0.175 |
| Abbreviations: HR=hazard ratio, CI=confidence interval | | | |
| [a] Importantly, the exposure assessment of chlorinated degreasers was probability-based by experts and evaluated as “possible” for catering workers. | | | |
| [b] Complete work history i.e. up to 8 employments as an offshore worker | | | |
| [c] Adjusted for age, age at first child, number of children, menopause status, and education | | | |
| [d] Missing: n=32 for both chlorinated degreasers and benzene. | | | |

For peer review only

**Directed acyclic graphs (DAGs)**

DAGs were produced by the DAGitty v3.0 software (Textor et al., 2016). Please note that the green circle denotes the exposure, the blue circle with I denotes the outcome, the red circles denote the ancestor of the exposure AND the outcome (i.e. confounders, which are included in the models), and blue circles denote ancestor of the outcome (not included in the models).

Reference
Textor J, Zander B, Gilthorpe MK, Liskiewicz M, Ellison G. Robust causal inference using directed acyclic graphs: the R package 'dagitty'. Int J Epidemiol. 2016;45(6):1887-1894.
Website: www.dagitty.net

Models estimating breast cancer risk in relation to work schedule

In the following, we show DAG for each model with breast cancer as the outcome (as displayed in Tables 2 and 4) in relation to work schedule, age, menopausal status, age at first child, number of children, and education. We made the following assumptions:

- Age and education were not caused by any of the other variables in the DAG
- Menopausal status was affected by age
- Age at first child was affected by education
- Number of children was affected by age at first child

For peer review only



For peer review only - http://bmjopen.bmj.com/site/about/guidelines.xhtml

**Figure S1**

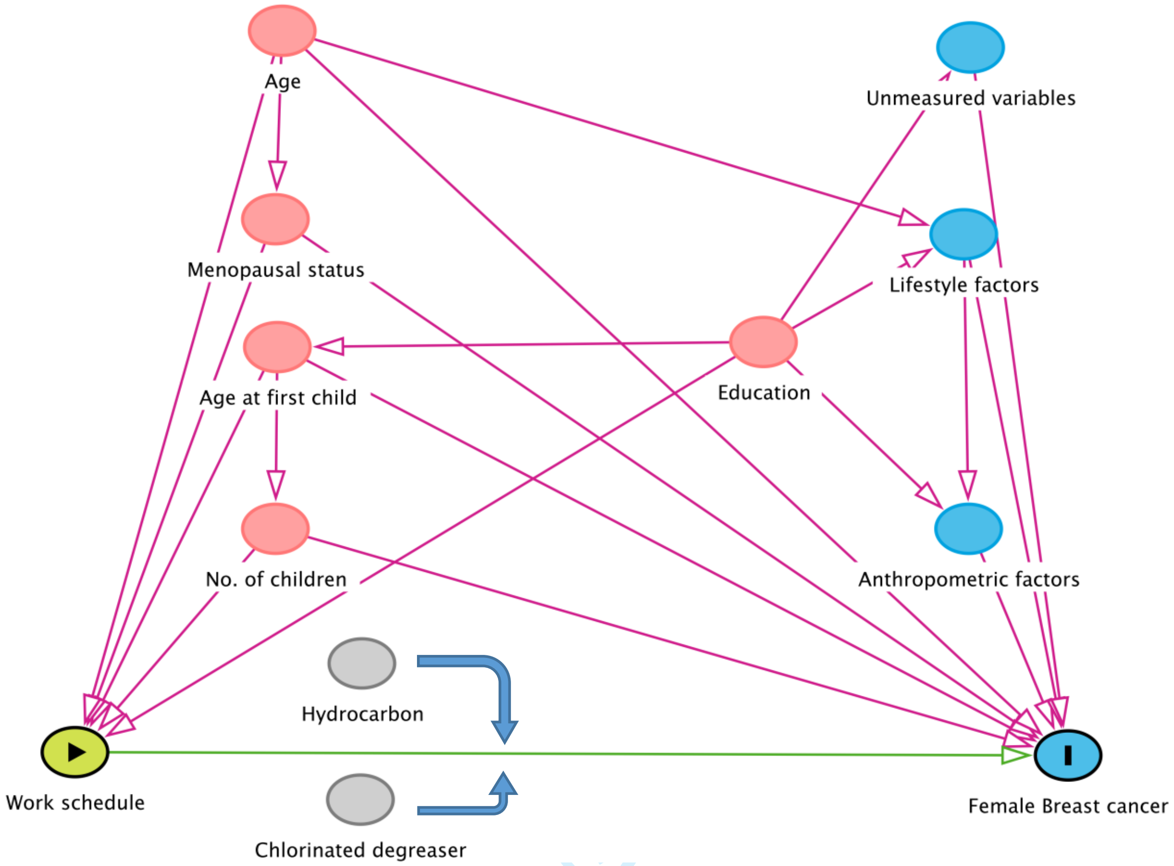


| Lifestyle factors: | Antropometric factors: | Unmeasured variables: |
|---|---|---|
| Fibre intake | Height | Chronotype |
| Multivitamine intake | Weight | Exogenous hormones use |
| Dietary fat intake | | Breast feeding |
| Alcohol intake | | Family history |
| Smoking | | |
| Physical activity | | |

view only



For peer review only - http://bmjopen.bmj.com/site/about/guidelines.xhtml

Models estimating breast cancer risk in relation to chemical co-exposures

In the following, we show DAG for each model with breast cancer as the outcome (as displayed in Tables 3) in relation to age, education, main occupational activity in last position. We made the following assumptions:

- Age, and education were not caused by any of the other variables in the DAG
- Main occupational activity in last position was affected by education, and is an instrument of chemical co-exposure.

For peer review only

**Figure S2**

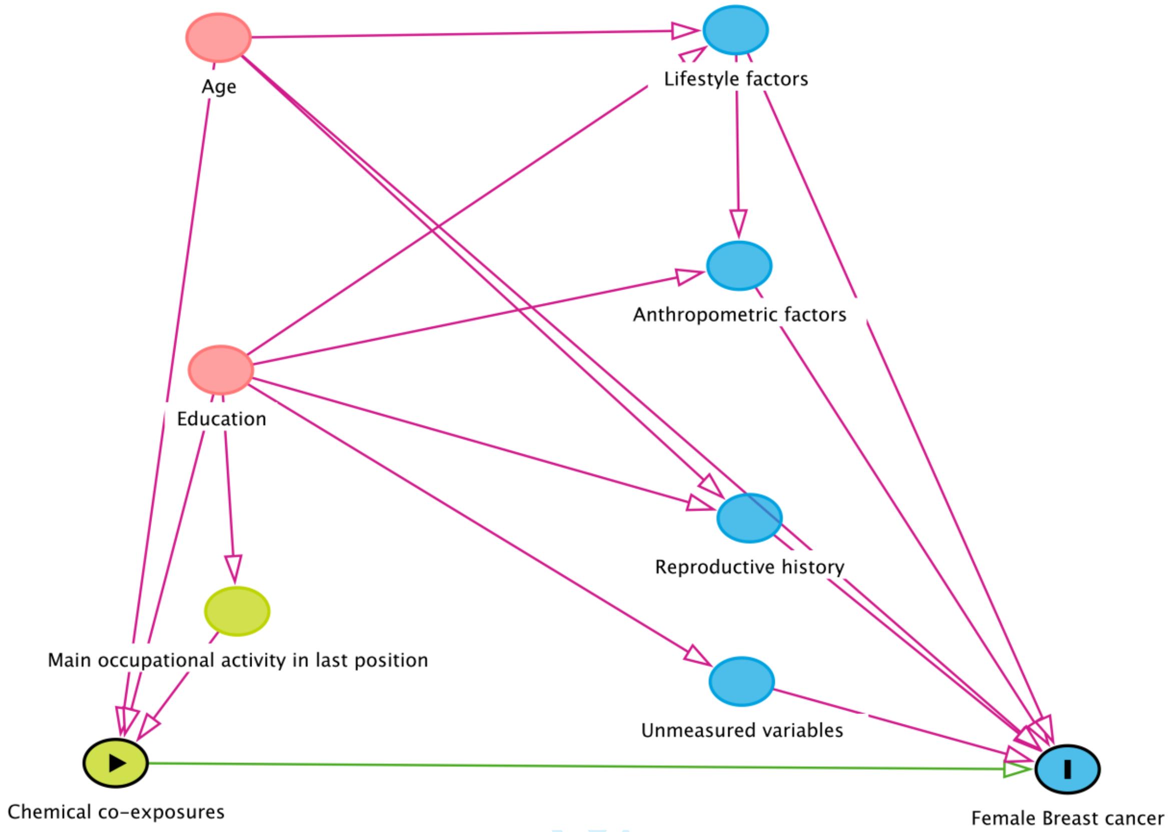


| **Chemical co-exposures**: | **Lifestyle factors:** | **Antropometric factors:** | **Reproductive history:** | **Unmeasured variables:** |
|---|---|---|---|---|
| Chlorinated degreaser | Fibre intake | Height | Age at first child | Exogenous hormones use |
| Benzene | Multivitamine intake | Weight | Number of children | Breast feeding |
| | Dietary fat intake | | Menopausal status | Family history |
| | Alcohol intake | | | |
| | Smoking | | | |
| | Physical activity | | | |



For peer review only - http://bmjopen.bmj.com/site/about/guidelines.xhtml

**SUPPLEMENTAL MATERIAL**

**Content:**

- Methods' extension on multiple imputations
- Table S1
- Table S2
- Table S3
- Table S4
- Table S5
- Table S6
- Directed acyclic graphs (DAGs)
    - Figure S1
    - Figure S2

For peer review only



For peer review only - http://bmjopen.bmj.com/site/about/guidelines.xhtml

**Methods' extension**

Multiple imputations

*Complete case analysis (n=367)*

As shown in Figure 1, the study sample for our complete case analysis included 49 breast cancer cases (with complete information on all variables in the regression model).

*Analysis of imputed data (n=600)*

As shown in Figure 1, our study sample yielded 86 breast cancer cases when those missing data retained. In this sample, 39% (n=233) had missing data on one or more of the following variables (missing numbers are displayed per variable):

- Work schedule involving night/rollover shift: n=32
- Duration of night/rollover shift: n=32
- Number of children: n=8
- Age at first child: n=192
- Education: n=8
- Main occupational activity in last position: n=7

Assuming missing at random, we used multiple imputation with chained equations and followed a 2-steps procedure:

1. We identified differences between these samples for date of birth, total employment duration, and breast cancer diagnosis, and used these variables as predictors together with the variables from the estimation model (i.e. work schedule involving night/rollover shift, duration of night/rollover shift, number of children, age at first child, education, and main



For peer review only - http://bmjopen.bmj.com/site/about/guidelines.xhtml

occupational activity in last position) to simultaneously predict missing data on all variables. Linear and multinomial logit models were used to predict missing data for continuous and categorical variables, respectively. The imputation model was ran 45 times, using the rule of thumb of running at least as many times as the percentage of missing data (White et al., 2011).

2. After step 1, we replicated the complete case analyses shown in Table 2, Table 3, and Table 4 on the imputed dataset (n=600).

In a sensitivity analysis (Table S4), we also tried an alternative imputation model where we excluded the variable 'age at first child', which had the highest number of missing (n=192, 39%). This reduced the total number of missing to n=53 (9%), but did not changed the results materially (Table S4).

Reference

White IR, Royston P, Wood AM. Multiple imputation using chained equations: Issues and guidance for practice. Statistics in medicine 2011; 30: 377-99.



For peer review only - http://bmjopen.bmj.com/site/about/guidelines.xhtml

# Table S1

| Table S1. ~~Characteristics~~Hazard Ratios of Female Breast Cancer ~~Cases~~According to Work Schedule in the Norwegian Offshore Petroleum Workers (NOPW) Cohort~~.~~, sensitivity analyses with menopause cut-off at age ≥51 | | | | |
|---|---|---|---|---|
| | **Complete case analysis (n=367)** | | | **Multiple imputation (n=600; 86 cases)** |
| **Work schedule variable** | **No. of participants** | **No. of cases** | **HR (95% CI)** | **HR (95% CI)** |
| Total employment duration [a,b] | | | | |
| Quartile 1 (0-1.9 years) | 79 | 9 | 1.00 (reference) | 1.00 (reference) |
| Quartile 2 (2-5.9 years) | 99 | 16 | 1.62 (0.59, 4.42) | 1.48 (0.68, 3.23) |
| Quartile 3 (6-10.9 years) | 90 | 8 | 0.69 (0.24, 2.00) | 1.61 (0.77, 3.38) |
| Quartile 4 (11-24 years) | 99 | 16 | 1.19 (0.46, 3.04) | 1.17 (0.54, 2.55) |
| P-trend [c] | | | 0.834 | 0.765 |
| Work schedule involving night/rollover shift [a,b] | | | | |
| Unexposed (day work only) | 217 | 29 | 1.00 (reference) | 1.00 (reference) |
| Exposed (night/rollover shift) | 150 | 20 | 1.05 (0.57, 1.96) | 0.86 (0.51, 1.45) |
| | | | | |
| Duration of night/rollover shift [a,b] | | | | |
| Unexposed (0 years, day work only) | 217 | 29 | 1.00 (reference) | 1.00 (reference) |
| ≤Median night/rollover (<1-6 years) | 68 | 6 | 0.71 (0.27, 1.86) | 0.55 (0.25, 1.21) |
| >Median night/rollover (>6 years) | 82 | 14 | 1.33 (0.66, 2.68) | 1.21 (0.67, 2.18) |
| P-trend [c] | | | 0.543 | 0.753 |
| Abbreviations: No=number, HR=hazard ratio, CI=confidence interval | | | | |
| [a] Adjusted for age, age at first child, number of children, menopause status (≥51 years as cut-off), and education | | | | |
| [b] complete work history i.e. up to 8 employments as an offshore worker | | | | |
| [c] Modelled as a continuous variable to test for linear trend. | | | | |

For peer review only

# Table S2

| Table S2. Hazard Ratios of Female Breast Cancer by Receptor Status Subtypes in Relation to Work Schedule in the Norwegian Offshore Petroleum Workers (NOPW) Cohort, sensitivity analyses with menopause cut-off at age ≥51 | | | | | |
|---|---|---|---|---|---|
| | | Complete case analysis (n=367) | | | Multiple imputation (n=600; 86 cases) |
| **Breast cancer subtype** | **Work schedule variable** | **No. of participants** | **No. of cases** | **HR (95% CI)** | **HR (95% CI)** |
| ER-positive | Work schedule involving night/rollover shift [a] | | | | |
| | Unexposed (day work only) | 217 | 23 | 1.00 (reference) | 1.00 (reference) |
| | Exposed (night/rollover shift) | 150 | 16 | 1.00 (0.50, 2.01) | 0.74 (0.41, 1.34) |
| ER-negative | Work schedule involving night/rollover shift [a] | | | | |
| | Unexposed (day work only) | 217 | 5 | 1.00 (reference) | 1.00 (reference) |
| | Exposed (night/rollover shift) | 150 | 2 | 0.76 (0.14, 4.17) | 1.53 (0.50, 4.70) |
| PR-positive | Work schedule involving night/rollover shift [a] | | | | |
| | Unexposed (day work only) | 217 | 25 | 1.00 (reference) | 1.00 (reference) |
| | Exposed (night/rollover shift) | 150 | 13 | 0.79 (0.38, 1.64) | 0.66 (0.35, 1.24) |
| PR-negative | Work schedule involving night/rollover shift [a] | | | | |
| | Unexposed (day work only) | 217 | 3 | 1.00 (reference) | 1.00 (reference) |
| | Exposed (night/rollover shift) | 150 | 5 | 2.08 (0.55, 7.88) | 1.72 (0.59, 5.02) |
| HER2-positive | Work schedule involving night/rollover shift [a] | | | | |
| | Unexposed (day work only) | 217 | 7 | 1.00 (reference) | 1.00 (reference) |
| | Exposed (night/rollover shift) | 150 | 1 | 0.27 (0.04, 1.91) | 0.21 (0.05, 0.98) |
| HER2-negative | Work schedule involving night/rollover shift [a] | | | | |
| | Unexposed (day work only) | 217 | 21 | 1.00 (reference) | 1.00 (reference) |
| | Exposed (night/rollover shift) | 150 | 14 | 1.04 (0.50, 2.16) | 0.88 (0.48, 1.62) |
| ER-positive and PR-positive and HER2-negative | Work schedule involving night/rollover shift [a] | | | | |
| | Unexposed (day work only) | 217 | 17 | 1.00 (reference) | 1.00 (reference) |
| | Exposed (night/rollover shift) | 150 | 9 | 0.80 (0.33, 1.94) | 0.58 (0.26, 1.28) |
| ER-positive and PR-positive and HER2-positive | Work schedule involving night/rollover shift [a] | | | | |
| | Unexposed (day work only) | 217 | 5 | 1.00 (reference) | 1.00 (reference) |
| | Exposed (night/rollover shift) | 150 | 1 | 0.35 (0.05, 2.36) | 0.27 (0.06, 1.26) |
| ER-negative and PR-negative and HER2-positive | Work schedule involving night/rollover shift [a] | | | | |
| | Unexposed (day work only) | 217 | 0 | 1.00 (reference) | 1.00 (reference) |
| | Exposed (night/rollover shift) | 150 | 0 | - | - |
| ER-negative and PR-negative and HER2-negative | Work schedule involving night/rollover shift [a] | | | | |
| | Unexposed (day work only) | 217 | 3 | 1.00 (reference) | 1.00 (reference) |
| | Exposed (night/rollover shift) | 150 | 2 | 1.34 (0.17, 11) | 2.22 (0.58, 8.45) |
| Abbreviations: No=number, HR=hazard ratio, CI=confidence interval, ER=oestrogen receptor, PR=progesterone receptor, HER2=human epidermal growth factor receptor 2 | | | | | |
| [a] Adjusted for age, age at first child, number of children, menopause status (≥51 years as cut-off), and education | | | | | |



For peer review only - http://bmjopen.bmj.com/site/about/guidelines.xhtml

# Table S3

| **Table S3**. Hazard Ratios of Female Breast Cancer According to Work Schedule in the Norwegian Offshore Petroleum Workers (NOPW) Cohort. Sensitivity Analyses of models adjusted for age at first child *versus* models without adjustment for age at first child. | | |
|---|---|---|
| | **Multiple imputation, including 'age at first child' (n=600; 86 cases) [a,b]** | **Multiple imputation, excluding 'age at first child' (n=600; 86 cases) [b,c]** |
| **Work schedule variable** | **HR (95% CI)** | **HR (95% CI)** |
| Total employment duration | | |
| Quartile 1 (0-1.9 years) | 1.00 (reference) | 1.00 (reference) |
| Quartile 2 (2-5.9 years) | 1.50 (0.69, 3.27) | 1.53 (0.71, 3.31) |
| Quartile 3 (6-10.9 years) | 1.64 (0.78, 3.50) | 1.65 (0.78, 3.47) |
| Quartile 4 (11-24 years) | 1.19 (0.54, 2.61) | 1.22 (0.56, 2.67) |
| P-trend [d1] | 0.733 | 0.685 |
| Work schedule involving night/rollover shift | | |
| Unexposed (day work only) | 1.00 (reference) | 1.00 (reference) |
| Exposed (night/rollover shift) | 0.87 (0.52, 1.46) | 0.87 (0.52, 1.45) |
| | | |
| Duration of night/rollover shift | | |
| Unexposed (0 years, day work only) | 1.00 (reference) | 1.00 (reference) |
| ≤Median night/rollover (<1-6 years) | 0.56 (0.26, 1.22) | 0.55 (0.25, 1.19) |
| >Median night/rollover (>6 years) | 1.21 (0.67, 2.18) | 1.21 (0.67, 2.17) |
| P-trend [d2] | 0.748 | 0.752 |
| Abbreviations: HR=hazard ratio, CI=confidence interval | | |
| [a] Adjusted for age, age at first child, number of children, menopause status (≥53 years as cut-off), and education. 39% (n=233) missing data imputed. | | |
| [b] complete work history i.e. up to 8 employments as an offshore worker | | |
| [c] Adjusted for variable in footnote "a" but without age at first child. 9% (n=53) missing data imputed. | | |
| [d1] Modelled as a continuous variable to test for linear trend. HR for excluding 'age at first child' 1.05 (95% CI: 0.84-1.31). | | |
| [d2] Modelled as a continuous variable to test for linear trend. HR for excluding 'age at first child' 1.05 (95% CI: 0.77-1.44). | | |

For peer review only



For peer review only - http://bmjopen.bmj.com/site/about/guidelines.xhtml

# Table S4

| **Table S4.** Characteristics of Female Breast Cancer Cases in the Norwegian Offshore Petroleum Workers (NOPW) Cohort. | |
|---|---|
| | **Breast Cancer Cases [a] (n=86)** |
| Age at diagnosis, mean (range) | 56 (38-80) |
| Clinical stage, n (%) | |
| I: No metastasis | 38 (44) |
| II: Regional lymph node metastasis | 29 (34) |
| III: Local infiltration into skin | 12 (14) |
| IV: Distant metastasis | 4 (5) |
| Unspecified | 3 (3) |
| Oestrogen receptors (ER), n (%) | |
| ER-positive | 67 (78) |
| ER-negative | 13 (15) |
| Missing | 6 (7) |
| Progesterone receptors (PR), n (%) | |
| PR-positive | 59 (69) |
| PR-negative | 21 (24) |
| Missing | 6 (7) |
| Human epidermal growth factor receptor-2 (HER2), n (%) | |
| HER2-positive | 12 (14) |
| HER2-negative | 60 (70) |
| Missing | 14 (16) |
| ~~Luminal A: HER2-negative and~~ ER-positive and~~/or~~ PR-positive and HER2-negative, n (%) | |
| Yes | ~~46 (54~~39 (45) |
| No | ~~37 (43~~30 (35) |
| Missing | ~~3 (3~~17 (20) |
| ~~Luminal B: HER2-positive and~~ ER-positive and~~/or~~ PR-positive and HER2-positive, n (%) | |
| Yes | 9 (10 ~~(12~~) |
| No | ~~73 (85~~60 (70) |
| Missing | ~~3 (3~~17 (20) |
| ER-negative and PR-negative and HER2-positive, n (%) | |
| Yes | 0 (0) |
| No | 69 (80) |
| Missing | 17 (20) |
| ~~Triple negative: HER2-negative and~~ ER-negative and PR-negative~~, n (%)~~ and HER2-negative | |
| Yes | 11 (13) |
| No | ~~72 (84~~58 (67) |
| Missing | ~~3 (3~~17 (20) |
| [a] According to International Classification of Diseases 10th edition : C50 | |



For peer review only - http://bmjopen.bmj.com/site/about/guidelines.xhtml

# Table S5

| **Table S5**. Hazard Ratios of Breast Cancer According to duration of night + rollover shift quartile in the Norwegian Offshore Petroleum Workers (NOPW) Cohort |
|---|



For peer review only - http://bmjopen.bmj.com/site/about/guidelines.xhtml

For peer review only

# Table S2

| Table S2. Hazard Ratios of Breast Cancer According to duration of night + rollover shift quartile in the Norwegian Offshore Petroleum Workers (NOPW) Cohort | | | | |
|---|---|---|---|---|
| | Complete case analysis (n=367) | | | Multiple imputation (n=600; 86 cases) |
| **Work schedule variable** | **No. of participants** | **No. of cases** | **HR (95% CI)** | **HR (95% CI)** |
| Duration of night/rollover shift [a,b] | | | | |
| Unexposed (0 years, day work only) | 217 | 29 | 1.00 (reference) | 1.00 (reference) |
| Quartile 1 night/rollover (<1-2 years) | 28 | 3 | 0.99 (0.28, 3.52) | 0.78 (0.28, 2.14) |
| Quartile 2 night/rollover (>2-5 years) | 32 | 2 | 0.52 (0.11, 2.47) | 0.49 (0.14, 1.71) |
| Quartile 3 night/rollover (>5-10 years) | 36 | 4 | 0.77 (0.24, 2.48) | 1.02 (0.47, 2.24) |
| Quartile 4 night/rollover (>10 years) | 54 | 11 | 1.62 (0.73, 3.59) | 1.10 (0.51, 2.37) |
| P-trend [c] | | | 0.506 | 0.944 |
| Abbreviations: No=number, HR=hazard ratio, CI=confidence interval | | | | |
| [a] Adjusted for age, age at first child, number of children, menopause status, , (≥53 years as cut-off), and education | | | | |
| [b] complete work history i.e. up to 8 employments as an offshore worker | | | | |
| [c] Modelled as a continuous variable to test for linear trend | | | | |

For peer review only

# Table ~~S3~~ S6

| |
|---|
| ~~**Table S3**. Hazard Ratios of Breast Cancer According to Work Schedule and Chemical Co-exposures in the Norwegian Offshore Petroleum Workers (NOPW) Cohort, multiple imputation analysis (n=601)~~ |

**Table S6**. Hazard Ratios of Breast Cancer According to Work Schedule and Chemical Co-exposures in the Norwegian Offshore Petroleum Workers (NOPW) Cohort, multiple imputation analysis (n=601)

| | **Chlorinated degreasers [a]** | | |
|---|---|---|---|
| **Work schedule variable [b,d]** | **Unexposed** | **Exposed** | **P Value** |
| Unexposed (day work only) | | | |
| n (%) | 99 (17) | 223 (39) | |
| HR [c] (95% CI) | 1.00 (reference) | 0.91 (0.45, 1.84) | |
| Exposed (night/rollover shift) | | | |
| n (%) | 43 (8) | 204 (36) | |
| HR [c] (95% CI) | 1.01 (0.40, 2.55) | 0.79 (0.37, 1.71) | |
| $P_{interaction}$ | | | 0.725 |
| | **Benzene** | | |
| **Work schedule variable [b,d]** | **Unexposed** | **Exposed** | **P Value** |
| Unexposed (day work only) | | | |
| n (%) | 269 (48) | 53 (9) | |
| HR [c] (95% CI) | 1.00 (reference) | 0.60 (0.21, 1.70) | |
| Exposed (night/rollover shift) | | | |
| n (%) | 199 (35) | 48 (8) | |
| HR [c] (95% CI) | 0.74 (0.41, 1.32) | 1.11 (0.46, 2.70) | |
| $P_{interaction}$ | | | 0.175 |

Abbreviations: HR=hazard ratio, CI=confidence interval

[a] Importantly, the exposure assessment of chlorinated degreasers was probability-based by experts and evaluated as "possible" for catering workers.

[b] Complete work history i.e. up to 8 employments as an offshore worker

[c] Adjusted for age, age at first child, number of children, menopause status, and education

[d] Missing: n=32 for both chlorinated degreasers and benzene.

For peer review only



For peer review only - http://bmjopen.bmj.com/site/about/guidelines.xhtml

## Table S4

| Table S4. Hazard Ratios of Female Breast Cancer According to Work Schedule in the Norwegian Offshore Petroleum Workers (NOPW) Cohort. Sensitivity Analyses of models adjusted for age at first child *versus* models without adjustment for age at first child. | | |
|---|---|---|
| | **Multiple imputation, including 'age at first child' (n=600; 86 cases)** [a,b] | **Multiple imputation, excluding 'age at first child' (n=600; 86 cases)** [b,c] |
| **Work schedule variable** | **HR (95% CI)** | **HR (95% CI)** |
| Total employment duration | | |
| Quartile 1 (0-1.9 years) | 1.00 (reference) | 1.00 (reference) |
| Quartile 2 (2-5.9 years) | 1.50 (0.69, 3.27) | 1.53 (0.71, 3.31) |
| Quartile 3 (6-10.9 years) | 1.64 (0.78, 3.50) | 1.65 (0.78, 3.47) |
| Quartile 4 (11-24 years) | 1.19 (0.54, 2.61) | 1.22 (0.56, 2.67) |
| P-trend [d] | 0.733 | 0.685 |
| Work schedule involving night/rollover shift | | |
| Unexposed (day work only) | 1.00 (reference) | 1.00 (reference) |
| Exposed (night/rollover shift) | 0.87 (0.52, 1.46) | 0.87 (0.52, 1.45) |
| | | |
| Duration of night/rollover shift | | |
| Unexposed (0 years, day work only) | 1.00 (reference) | 1.00 (reference) |
| ≤Median night/rollover (<1-6 years) | 0.56 (0.26, 1.22) | 0.55 (0.25, 1.19) |
| >Median night/rollover (>6 years) | 1.21 (0.67, 2.18) | 1.21 (0.67, 2.17) |
| P-trend [d] | 0.748 | 0.752 |
| Abbreviations: HR=hazard ratio, CI=confidence interval | | |
| [a] Adjusted for age, age at first child, number of children, menopause status, and education. 39% (n=233) missing data imputed. | | |
| [b] complete work history i.e. up to 8 employments as an offshore worker | | |
| [c] Adjusted for variable in footnote "a" but without age at first child. 9% (n=53) missing data imputed. | | |
| [d] Modelled as a continuous variable to test for linear trend | | |

For peer review only



For peer review only - http://bmjopen.bmj.com/site/about/guidelines.xhtml

**Directed acyclic graphs (DAGs)**

DAGs were produced by the DAGitty v3.0 software (Textor et al., 2016). Please note that the green circle denotes the exposure, the blue circle with I denotes the outcome, the red circles denote the ancestor of the exposure AND the outcome (i.e. confounders, which are included in the models), and blue circles denote ancestor of the outcome (not included in the models).

Reference
Textor J, Zander B, Gilthorpe MK, Liskiewicz M, Ellison G. Robust causal inference using directed acyclic graphs: the R package 'dagitty'. Int J Epidemiol. 2016;45(6):1887-1894.
Website: www.dagitty.net

Models estimating breast cancer risk in relation to work schedule

In the following, we show DAG for each model with breast cancer as the outcome (as displayed in Tables 2 and 4) in relation to work schedule, age, menopausal status, age at first child, number of children, and education. We made the following assumptions:

- Age and education were not caused by any of the other variables in the DAG
- Menopausal status was affected by age
- Age at first child was affected by education
- Number of children was affected by age at first child



For peer review only - http://bmjopen.bmj.com/site/about/guidelines.xhtml

**Figure S1**

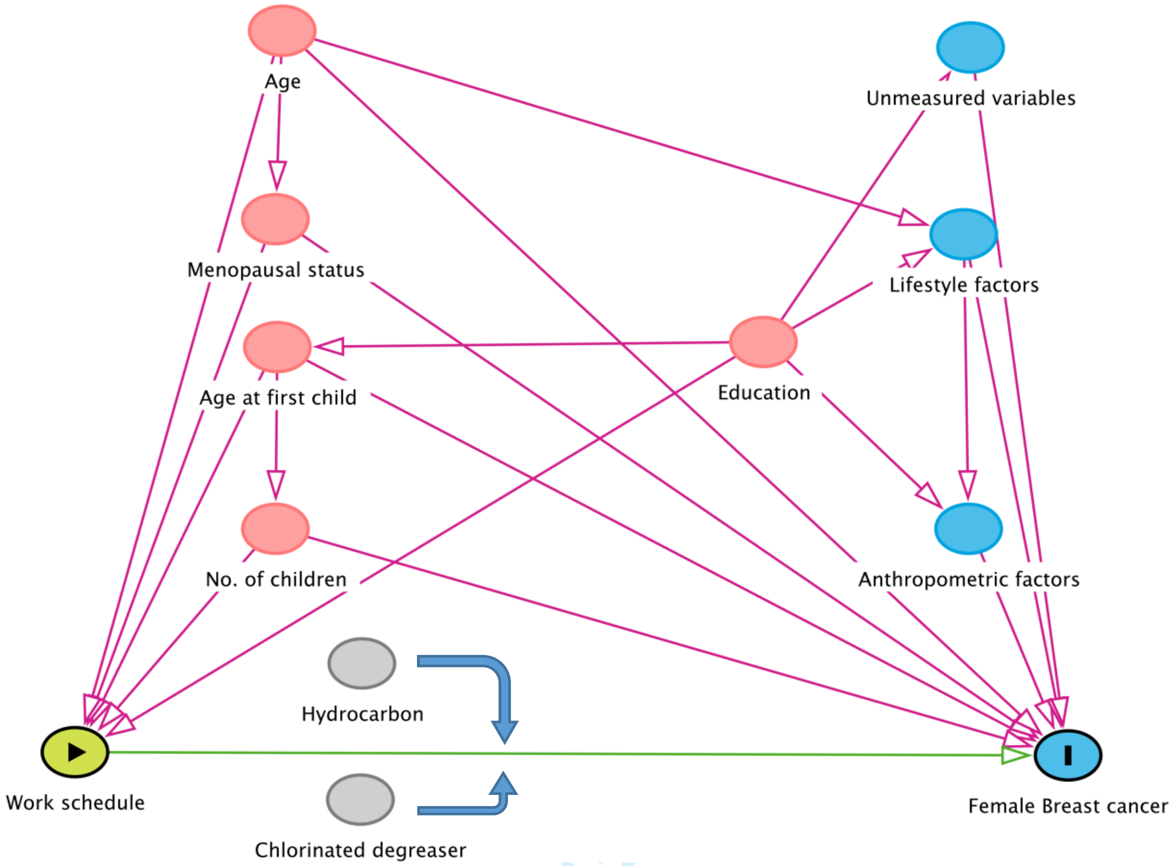


| Lifestyle factors: | Antropometric factors: | Unmeasured variables: |
|---|---|---|
| Fibre intake | Height | Chronotype |
| Multivitamine intake | Weight | Exogenous hormones use |
| Dietary fat intake | | Breast feeding |
| Alcohol intake | | Family history |
| Smoking | | |
| Physical activity | | |



For peer review only - http://bmjopen.bmj.com/site/about/guidelines.xhtml

Models estimating breast cancer risk in relation to chemical co-exposures

In the following, we show DAG for each model with breast cancer as the outcome (as displayed in Tables 3) in relation to age, education, main occupational activity in last position. We made the following assumptions:

- Age, and education were not caused by any of the other variables in the DAG
- Main occupational activity in last position was affected by education, and is an instrument of chemical co-exposure.

For peer review only



For peer review only - http://bmjopen.bmj.com/site/about/guidelines.xhtml

**Figure S2**

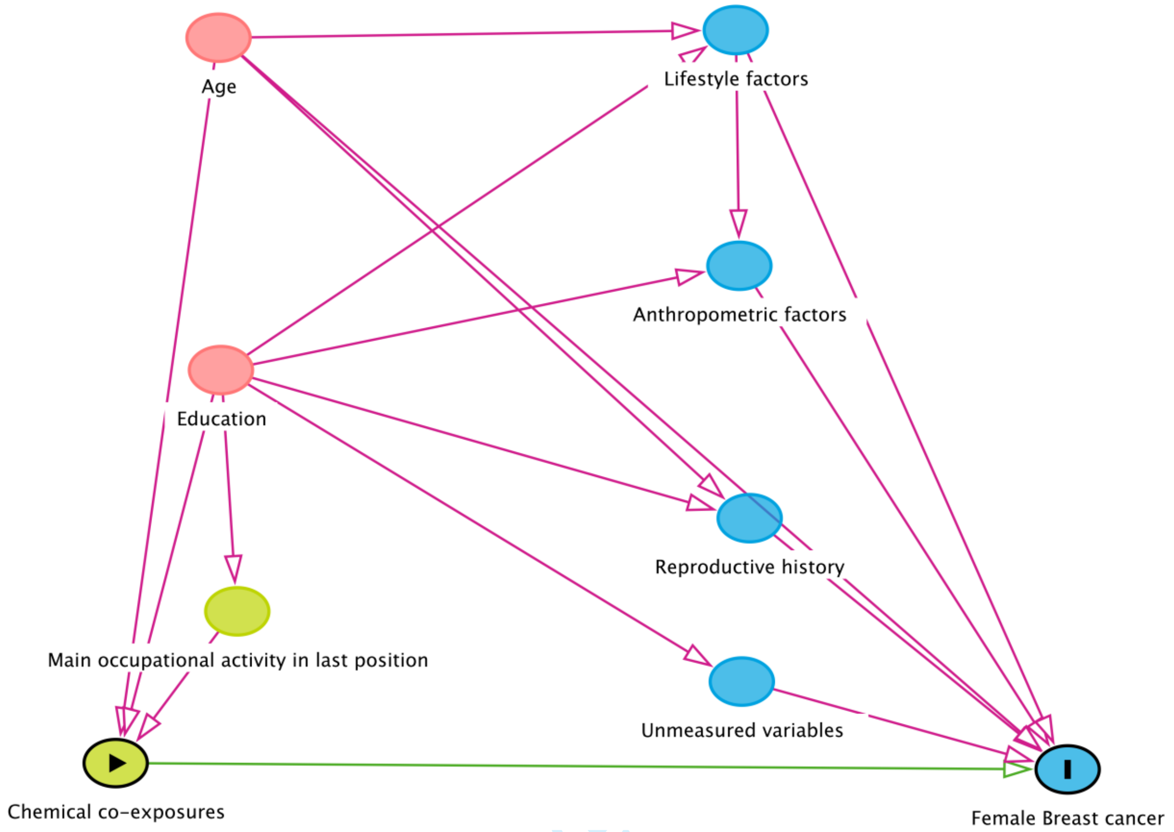


| **Chemical co-exposures**: | **Lifestyle factors:** | **Antropometric factors:** | **Reproductive history:** | **Unmeasured variables:** |
|---|---|---|---|---|
| Chlorinated degreaser | Fibre intake | Height | Age at first child | Exogenous hormones use |
| Benzene | Multivitamine intake | Weight | Number of children | Breast feeding |
| | Dietary fat intake | | Menopausal status | Family history |
| | Alcohol intake | | | |
| | Smoking | | | |
| | Physical activity | | | |



For peer review only - http://bmjopen.bmj.com/site/about/guidelines.xhtml

STROBE Statement—Checklist of items that should be included in reports of ***cohort studies***

| | Item No | Recommendation | Page No |
|---|---|---|---|
| **Title and abstract** | 1 | (*a*) Indicate the study's design with a commonly used term in the title or the abstract<br>(*b*) Provide in the abstract an informative and balanced summary of what was done and what was found | p. 1-2 |
| **Introduction** | | | |
| Background/rationale | 2 | Explain the scientific background and rationale for the investigation being reported | p. 4-5 |
| Objectives | 3 | State specific objectives, including any prespecified hypotheses | p. 5 |
| **Methods** | | | |
| Study design | 4 | Present key elements of study design early in the paper | p. 6 |
| Setting | 5 | Describe the setting, locations, and relevant dates, including periods of recruitment, exposure, follow-up, and data collection | p. 5-6 |
| Participants | 6 | (*a*) Give the eligibility criteria, and the sources and methods of selection of participants. Describe methods of follow-up<br>(*b*) For matched studies, give matching criteria and number of exposed and unexposed | p. 7 |
| Variables | 7 | Clearly define all outcomes, exposures, predictors, potential confounders, and effect modifiers. Give diagnostic criteria, if applicable | p. 6-10 |
| Data sources/ measurement | 8* | For each variable of interest, give sources of data and details of methods of assessment (measurement). Describe comparability of assessment methods if there is more than one group | p. 6-10 |
| Bias | 9 | Describe any efforts to address potential sources of bias | p. 6-11 |
| Study size | 10 | Explain how the study size was arrived at | p. 7, Figure 1 |
| Quantitative variables | 11 | Explain how quantitative variables were handled in the analyses. If applicable, describe which groupings were chosen and why | p. 6-11 |
| Statistical methods | 12 | (*a*) Describe all statistical methods, including those used to control for confounding<br>(*b*) Describe any methods used to examine subgroups and interactions<br>(*c*) Explain how missing data were addressed<br>(*d*) If applicable, explain how loss to follow-up was addressed<br>(*e*) Describe any sensitivity analyses | p. 10-11 |
| **Results** | | | |
| Participants | 13* | (a) Report numbers of individuals at each stage of study—eg numbers potentially eligible, examined for eligibility, confirmed eligible, included in the study, completing follow-up, and analysed<br>(b) Give reasons for non-participation at each stage<br>(c) Consider use of a flow diagram | p. 7, Figure 1 |
| Descriptive data | 14* | (a) Give characteristics of study participants (eg demographic, clinical, social) and information on exposures and potential confounders | p. 11-12, Table 1 + Table S1 |



For peer review only - http://bmjopen.bmj.com/site/about/guidelines.xhtml

| | | | |
|---|---|---|---|
| | | (b) Indicate number of participants with missing data for each variable of interest<br>(c) Summarise follow-up time (eg, average and total amount) | |
| Outcome data | 15* | Report numbers of outcome events or summary measures over time | p. 11-13, Figure 1 |

For peer review only


For peer review only - http://bmjopen.bmj.com/site/about/guidelines.xhtml

| | | | |
|---|---|---|---|
| Main results | 16 | (*a*) Give unadjusted estimates and, if applicable, confounder-adjusted estimates and their precision (eg, 95% confidence interval). Make clear which confounders were adjusted for and why they were included<br><br>(*b*) Report category boundaries when continuous variables were categorized<br>(*c*) If relevant, consider translating estimates of relative risk into absolute risk for a meaningful time period | p. 12-13, Table 2 |
| Other analyses | 17 | Report other analyses done—eg analyses of subgroups and interactions, and sensitivity analyses | p. 11-12, Table 3 + Table 4 + Table S2 + Table S3 + Table S4 + Table S5 + Table S6 |
| **Discussion** | | | |
| Key results | 18 | Summarise key results with reference to study objectives | p. 13 |
| Limitations | 19 | Discuss limitations of the study, taking into account sources of potential bias or imprecision. Discuss both direction and magnitude of any potential bias | p. 14-15 |
| Interpretation | 20 | Give a cautious overall interpretation of results considering objectives, limitations, multiplicity of analyses, results from similar studies, and other relevant evidence | p. 13-15 |
| Generalisability | 21 | Discuss the generalisability (external validity) of the study results | p. 15 |
| **Other information** | | | |
| Funding | 22 | Give the source of funding and the role of the funders for the present study and, if applicable, for the original study on which the present article is based | p. 16 |

*Give information separately for exposed and unexposed groups.

**Note:** An Explanation and Elaboration article discusses each checklist item and gives methodological background and published examples of transparent reporting. The STROBE checklist is best used in conjunction with this article (freely available on the Web sites of PLoS Medicine at http://www.plosmedicine.org/, Annals of Internal Medicine at http://www.annals.org/, and Epidemiology at http://www.epidem.com/). Information on the STROBE Initiative is available at http://www.strobe-statement.org.

For peer review only



For peer review only - http://bmjopen.bmj.com/site/about/guidelines.xhtml

BMJ Open

# Night shift work, chemical co-exposures and risk of female breast cancer in the Norwegian Offshore Petroleum Workers (NOPW) cohort: a prospectively-recruited case-cohort study.

| | |
|---|---|
| Journal: | *BMJ Open* |
| Manuscript ID | bmjopen-2021-056396.R2 |
| Article Type: | Original research |
| Date Submitted by the Author: | 24-Dec-2021 |
| Complete List of Authors: | Liu, Fei-Chih; Cancer Registry of Norway, Department of Research; University of Oslo Faculty of Medicine, Department of Biostatistics<br>Veierød, Marit ; University of Oslo Faculty of Medicine, Department of Biostatistics<br>Kjærheim, Kristina; Cancer Registry of Norway, Department of Research<br>Robsahm, Trude; Cancer Registry of Norway, Department of Research<br>Ghiasvand, Reza; Cancer Registry of Norway, Department of Research; Oslo University Hospital, Oslo Centre for Biostatistics and Epidemiology<br>Hosgood, Dean; Albert Einstein College of Medicine, Department of Epidemiology and Population Health<br>Samuelsen, Sven Ove; University of Oslo, Department of Mathematics<br>Bråtveit, Magne; Universitetet i Bergen Det medisinsk-odontologiske fakultet, Department of Global Public Health and Primary Care<br>Kirkeleit, Jorun; Universitetet i Bergen Det medisinsk-odontologiske fakultet, Department of Global Health and Primary Care<br>Rothman, Nat; National Cancer Institute, Occupational and Environmental Epidemiology<br>Lan, Qing; National Cancer Institute, Occupational and Environmental Epidemiology<br>Silverman, Debra; National Cancer Institute, Occupational and Environmental Epidemiology<br>Friesen, Melissa C.; National Cancer Institute, Occupational and Environmental Epidemiology<br>Babigumira, Ronnie; Cancer Registry of Norway, Department of Research<br>Shala, Nita; Cancer Registry of Norway, Department of Research; University of Oslo Faculty of Medicine, Department of Biostatistics<br>Grimsrud, Tom; Cancer Registry of Norway, Department of Research<br>Stenehjem, Jo; Cancer Registry of Norway, Department of Research |
| <b>Primary Subject Heading</b>: | Epidemiology |
| Secondary Subject Heading: | Oncology, Public health, Occupational and environmental medicine |
| Keywords: | EPIDEMIOLOGY, Breast tumours < ONCOLOGY, OCCUPATIONAL & INDUSTRIAL MEDICINE |
| | |

SCHOLARONE™
Manuscripts

*I, the Submitting Author has the right to grant and does grant on behalf of all authors of the Work (as defined in the below author licence), an exclusive licence and/or a non-exclusive licence for contributions from authors who are: i) UK Crown employees; ii) where BMJ has agreed a CC-BY licence shall apply, and/or iii) in accordance with the terms applicable for US Federal Government officers or employees acting as part of their official duties; on a worldwide, perpetual, irrevocable, royalty-free basis to BMJ Publishing Group Ltd ("BMJ") its licensees and where the relevant Journal is co-owned by BMJ to the co-owners of the Journal, to publish the Work in this journal and any other BMJ products and to exploit all rights, as set out in our licence.*

*The Submitting Author accepts and understands that any supply made under these terms is made by BMJ to the Submitting Author unless you are acting as an employee on behalf of your employer or a postgraduate student of an affiliated institution which is paying any applicable article publishing charge ("APC") for Open Access articles. Where the Submitting Author wishes to make the Work available on an Open Access basis (and intends to pay the relevant APC), the terms of reuse of such Open Access shall be governed by a Creative Commons licence – details of these licences and which Creative Commons licence will apply to this Work are set out in our licence referred to above.*

*Other than as permitted in any relevant BMJ Author's Self Archiving Policies, I confirm this Work has not been accepted for publication elsewhere, is not being considered for publication elsewhere and does not duplicate material already published. I confirm all authors consent to publication of this Work and authorise the granting of this licence.*

For peer review only

# MAIN DOCUMENT

**Title:** Night shift work, chemical co-exposures and risk of female breast cancer in the Norwegian Offshore Petroleum Workers (NOPW) cohort: a prospectively-recruited case-cohort study.

**Authors:** Fei-Chih Liu[1,2], Marit Bragelien Veierød[2], Kristina Kjærheim[1], Trude Eid Robsahm[1], Reza Ghiasvand[1,3], H. Dean Hosgood[4], Sven Ove Samuelsen[5], Magne Bråtveit[6], Jorun Kirkeleit[6], Nathaniel Rothman[7], Qing Lan[7], Debra T. Silverman[7], Melissa C. Friesen[7], Ronnie Babigumira[1], Nita K. Shala[1,2], Tom K. Grimsrud[1], Jo S. Stenehjem[1].

1) Department of Research, Cancer Registry of Norway, Institute of Population based Cancer Research, Oslo, Norway.
2) Oslo Centre for Biostatistics and Epidemiology, Department of Biostatistics, Institute of Basic Medical Sciences, University of Oslo, Norway.
3) Oslo Centre for Biostatistics and Epidemiology, Oslo University Hospital, Norway.
4) Department of Epidemiology and Population Health, Albert Einstein College of Medicine, Bronx, NY, United States.
5) Department of Mathematics, University of Oslo, Oslo, Norway.
6) Department of Global Public Health and Primary Care, University of Bergen, Bergen, Norway.
7) Occupational and Environmental Epidemiology Branch, Division of Cancer Epidemiology and Genetics, National Cancer Institute, Bethesda, MD, United States

Address correspondence to:
Jo S. Stenehjem, PhD
Post: Cancer Registry of Norway, P.O. Box 5313 Majorstuen, NO-0304 Oslo, Norway
E-mail: jo.stenehjem@kreftregisteret.no
Phone: +47 905 09 570

Word-count: 222 (abstract) 3,607 (main text)

Number of references: 47

Number of tables and figures: 5

Conflict of interest: None declared.



For peer review only - http://bmjopen.bmj.com/site/about/guidelines.xhtml

**ABSTRACT**

**Objectives:** This study examined the association between night shift work and risk of breast cancer, overall and by hormone receptor subtype, among females in the Norwegian Offshore Petroleum Workers (NOPW) cohort. We also examined the association of co-exposure (chlorinated degreasers and benzene) and breast cancer risk, and possible interaction with work schedule.

**Design:** Prospectively-recruited case-cohort study within the NOPW cohort.

**Setting:** Female offshore petroleum workers active on the Norwegian continental shelf.

**Participants**: 600 female workers (86 cases and 514 non-cases) were included in the study. We excluded workers that died or emigrated before start of follow-up, had missing work history, were diagnosed with breast cancer or other prior malignancy (except non-melanoma skin cancer) before start of follow-up.

**Results:** No overall association was found between breast cancer risk and work schedule (hazard ratio=0.87, 95% confidence interval: 0.52, 1.46 for work schedule involving night shift *vs*. day shift only). There was no significant association between work schedule and risk of any breast cancer subtype. No significant interactions were found between work schedule and chemical co-exposures (breast cancer overall $P_{\text{interaction chlorinated degreasers}}=0.725$ and $P_{\text{interaction benzene}}=0.175$).

**Conclusions:** Our results did not provide supporting evidence that work schedule involving night shift affects breast cancer risk in female offshore petroleum workers, but should be considered cautiously due to few cases. Further studies with larger sample sizes are warranted.



For peer review only - http://bmjopen.bmj.com/site/about/guidelines.xhtml

## Strengths and limitations of this study

- Complete cancer and population registry data ensure complete follow-up.
- Comprehensive work history information of night/rollover shift duration for up to eight employments per worker.
- Job-exposure matrices of chemical exposure developed specifically for cancer studies in the cohort.
- Females in the cohort were young at baseline, which yielded relatively few breast cancer cases.
- We lacked information on family history of breast cancer, exogenous hormone use and chronotype.

For peer review only

## INTRODUCTION

Breast cancer is the most frequent cancer among females and the incidence is higher in industrialised compared to developing countries [1]. The increase seen in industrialised societies has mainly been attributed to reproduction and lifestyle factors [2, 3]. However, the use of artificial light during work at night, leading to disruption of the internal circadian clock and suppressed melatonin production, may accelerate the transformation of normal cells to malignant mammary cells and promote the risk of breast cancer [4, 5].

Norwegian offshore workers employed at oil and gas installations have an extreme touring work pattern [6]. A standard two-week tour on Norwegian installations is minimum 12 hours of work seven days per week. The work schedule is either 14 day shifts, 14 night shifts or 14 rollover shifts (starting with 7 days followed by 7 nights or *vice versa*) [6]. During a night shift, offshore workers are constantly exposed to artificial light [6]. These extreme working hours meet the definition of night shift work (≥7 hours of work including the period from midnight to 05:00 hours) [7, 8].

The relationship between night shift work and breast cancer is still unclear. Several studies have reported a positive association between night shift work and breast cancer [9-12], while others did not [13-16]. A challenge has been the lack of high-quality exposure data on light during night time and detailed data on night shift work schedules [4]. A recent pooled analysis of five population-based case-control studies with complete work history reported an increased risk of breast cancer in pre-menopausal females who had high intensity and long duration of night shift work [17]. Further, night shift work may be associated with an increased risk of oestrogen receptor (ER)-positive, progesterone receptor (PR)-positive or human epidermal growth factor receptor 2 (HER2)-positive breast cancer [18-21]. However, night shift work has also been reported to increase the risk of ER-negative breast cancer [22],



For peer review only - http://bmjopen.bmj.com/site/about/guidelines.xhtml

and whether the association between night shift work and breast cancer varies by subtype remains unclear.

Based on the limited evidence for the carcinogenicity of night shift work, the International Agency for Research on Cancer (IARC) recently reconfirmed night shift work as a probable carcinogen to humans (Group 2A) [23], asking for improved exposure assessment in future studies to reduce heterogeneity between studies and allow for better comparisons. IARC also encouraged better reporting of potential co-exposures to occupational carcinogens. On offshore petroleum installations, some female workers may be exposed to a number of carcinogens, but in particular benzene during drilling, production and maintenance [24, 25], and historically chlorinated degreasers during cleaning of metal parts (trichloroethylene) or dry-cleaning of work clothes (tetrachloroethylene) [21]. Cohort and population-based studies have linked benzene and tetrachloroethylene to increased breast cancer risk [26, 27], and recently, night shift work has received increased attention as a possible effect modifier of the association between chemical exposure and cancer by desynchronising detoxification mechanisms [28, 29]. To our knowledge, chemical exposure and night shift work have not been examined simultaneously in relation to breast cancer risk.

The Norwegian Offshore Petroleum Workers (NOPW) cohort holds information on work schedule for each individual employed 1965–1998 and job exposure matrices (JEMs) capturing chemical exposures in the offshore work environment [30]. The aim of the present study was to examine the association between shift work and breast cancer risk, overall and by receptor status subtypes, among prospectively-recruited females in the NOPW cohort. We also examined the association of chemical exposures (chlorinated degreasers and benzene) and breast cancer risk, and possible multiplicative interactions with work schedule.



For peer review only - http://bmjopen.bmj.com/site/about/guidelines.xhtml

## MATERIALS AND METHODS

### Patient and public involvement

This study was conducted without patient involvement.

### Study population and study design

The NOPW cohort was established in 1998 by the Cancer Registry of Norway (CRN). Questionnaires were filled in by current or former male and female offshore petroleum workers with a minimum of 20 days work on the Norwegian continental shelf between 1965 and 1998 [30]. In total, 27,917 workers were included (estimated response rate 69%) in the NOPW cohort [31]. Details on the cohort and its establishment have been published elsewhere [30, 31]. All workers filled in an informed consent for participation in the study. Necessary legal and ethical approvals have been obtained from the Norwegian Data Inspectorate, the Regional Committee for Medical Research Ethics (2018/1162), and the Norwegian Directorate of Health.

We used a stratified case-cohort design [32] as complete information on work history had to be extracted manually from the questionnaires for breast cancer cases and a random subsample of the cohort (hereafter the 'subcohort'). The case-cohort design allows prospective models such as Cox-regression, and the subcohort may be used as controls for different types of cancer cases, as it is not matched to specific cases.

### Identification of cancer cases

The NOPW cohort was linked to the CRN and the National Population Register for information on cancer, death and emigration, by using the unique personal identification numbers assigned to all Norwegian citizens [33]. Follow-up started 1 July 1999 and ended 31 December 2017. Reporting of incident cancers to the CRN is mandatory in Norway and the



For peer review only - http://bmjopen.bmj.com/site/about/guidelines.xhtml

degree of completeness and validity is high, with morphology verified for 99.3% of the breast cancers [33]. Cancer cases were defined according to the International Classification of Diseases, Tenth Revision (ICD-10), C50 for breast cancer. Immunohistochemistry and pathology reports were submitted routinely to the CRN to assess breast cancer receptor status.

**Study sample**

A total of 25,347 males were excluded from the full cohort, and breast cancer cases and the subcohort were identified among the remaining 2,570 female offshore workers (Figure 1). Among the cases, we applied the exclusion criteria (i) death or emigration before start of follow-up (n=0) (ii) missing work history (n=10), (iii) breast cancer before start of follow-up (n=7), and (iv) other prior malignancy except non-melanoma skin cancer before start of follow-up (mainly squamous cell carcinoma as the CRN does not routinely record information on basal cell carcinoma of the skin [n=0]), resulting in 86 breast cancer cases in the analyses (Figure 1).

The initial subcohort (n=557) was sampled from the female cohort (n=2,570) and was established in two steps to secure at least 5 non-cases per case. First, we oversampled (*i.e.* selected all) the female cohort members born 1920–1939 and 1975–1979 (n=102) to secure that the oldest and the youngest female non-cases were comparable to the cases in these birth cohorts. Second, for those born 1940–1974, we drew at random within strata of 5-year birth cohorts 455 female workers, who were frequency matched to the birth year distribution of all potentially occupational cancers estimated for 2017 (n=91). We excluded 43 females that died or emigrated before start of follow-up (n=1), had missing work history (n=3), were diagnosed with breast cancer (n=5) or other prior malignancy (except non-melanoma skin cancer [n=12]) before start of follow-up, or were diagnosed with breast cancer during follow-up (cases, n=22). Thus, for our analyses, 514 females remained in the subcohort (Figure 1).



For peer review only - http://bmjopen.bmj.com/site/about/guidelines.xhtml

**Exposure assessment**

*Work schedule*

We handled work history data of 514 female subcohort members and 86 cases who reported up to eight employments each. The process of harmonising overlapping employment records was handled by collapsing jobs within the same category and by splitting jobs of different categories into proportionally equal parts, according to a method described previously [34].

The self-reported work schedule for each job was recorded as either day, night, or rollover shift for two-week tours. Day shift is equivalent to 14 consecutive days of day-time work usually between 07:00 and 19:00 hours. Night shift is equivalent to 14 consecutive days of night-time work usually between 19:00 and 07:00 hours. Rollover shift is equivalent to 7 consecutive days of day-time work (same working hours as for day shift) then alternate to 7 consecutive days of night-time work (same working hours as for night shift), or *vice versa*.

Some worked only day, but none worked only night or only rollover. Therefore, the variable work schedule was categorised as 1) day work only, 2) mixed I (some night/rollover + mainly day), 3) mixed II (mainly night/rollover + some day), and 4) missing. We then collapsed categories 2 (mixed I) and 3 (mixed II) into exposed (night/rollover shift) to represent workers that had ever worked some degree of night-time. For each individual, the duration of night/rollover shift was calculated by (i) cumulating the total number of years with night shifts, (ii) cumulating the total number of years with rollover shifts, (iii) summarising the number of years worked with night and rollover shifts into a combined continuous variable, and (iv) categorising this variable according to median (unexposed, <1-6, >6) or quartile (unexposed, <1–2, >2–6, >6–10, >10) of years of night/rollover shift. Total employment duration was categorised according to quartiles (0–1.9, 2–5.9, 6–10.9, 11–24), expressed in years.



For peer review only - http://bmjopen.bmj.com/site/about/guidelines.xhtml

*Chemical co-exposures and main occupational activity in last position*

A research group at the University of Bergen has developed expert-based job-exposure matrices (JEMs) for the NOPW cohort to identify and describe the degree of exposure to agents, mixtures or exposure situations with known or suspected carcinogenic potential among offshore workers on the Norwegian continental shelf 1970–2005 [25, 35]. The JEMs and their development have been described in detail elsewhere [25, 36]. In brief, due to lack of exposure measurements, the assessment of the JEM for chlorinated degreasers was probability-oriented and based on summary documents (*e.g.* from company visits/interviews, risk assessment reports, sampling reports, product data sheets) on the exposure to tri- and tetrachloroethylene. Eight experts individually categorised the likelihood (unlikely, possible, probable) of chlorinated degreaser exposure (*i.e.* products containing tri- and tetrachloroethylene) for 27 job categories [35]. Importantly, the exposure assessment of chlorinated degreasers was evaluated as "possible" for catering workers, meaning that some workers may have been exposed while the probability of exposure was low. For the benzene JEM, measurement data were used to construct a time-line of exposure and to rate exposure intensity of tasks specific to each job category. Then, task duration and frequency were combined to create a semi-quantitative benzene exposure burden score specific to job category and time period (1965–1969, 1970–1979, 1980–1989, 1990–1998) [36]. For each of the two agents, we generated a dichotomized variable indicating never or ever exposure.

Main occupational activity in each worker's last position was recorded as production and process, drilling and well maintenance, maintenance/inspection/deck construction, catering/office/administration, and miscellaneous.

*Covariates*



For peer review only - http://bmjopen.bmj.com/site/about/guidelines.xhtml

Age at first child and number of children were recorded in the questionnaire, categorized as 0, 1, 2, ≥3 children. Females aged ≥53 years at baseline in 1998 were assumed postmenopausal, according to the convention from the Million Women Study [37] which also was adopted by the Norwegian Women and Cancer study [38] that is representative of the Norwegian female population. In supplemental analyses, we also used age ≥51 as cut-point to define postmenopause (Supplemental Material, Table S1, Table S2), which also has been used in previous study [39]. Education was recorded as compulsory, vocational, folk high school, upper secondary (the two latter collapsed into upper secondary), and university/college.

**Data analysis**

Cox regression, adapted to a stratified case-cohort design [32], was used to estimate hazard ratios (HRs) of breast cancer and 95% confidence intervals (CIs) associated with work schedule, chlorinated degreasers and benzene. Cases were assigned a weight of 1 and subcohort non-cases were given weights according to the inverse sampling fraction in their corresponding 5-year birth cohort stratum. For those born 1940-1974, we drew at random within the 5-year birth cohorts and calculated a sampling fraction, defined as random sample / total cohort member, for each birth cohort. We then assigned a weight, which was defined as 1/sampling fraction, for each birth cohort and those weights were specified as offset in the Cox regression models. In the analyses, age was used as the time-scale with subjects entering start of follow-up on 30 June 1999. Subjects were censored at the date of any cancer diagnosis (except non-melanoma skin cancer), emigration, death, or end of study, whichever came first. The 22 cases identified as members of the randomly drawn subcohort were analysed as cases only (weight=1), and they are included in the (weighted) Cox-likelihood at every event time they are at risk according to the Borgan II estimator [32]. Robust variance was used to

For peer review only

compute standard errors of the HRs. The proportional hazards assumption was evaluated by Schoenfeld residuals.

Based on the literature and the available variables in NOPW, a directed acyclic graph (DAG) was drawn to help model the relationship between work schedule and breast cancer risk (Figure S1, Supplemental Material). Based on the DAG, we adjusted for age at first child, number of children, menopausal status at baseline and education in addition to attained age (timescale). Likewise, based on a DAG (Supplemental Material, Figure S2) we adjusted for age and education in the analyses of chlorinated degreasers and benzene in relation to breast cancer risk. We tested for linear trend by modelling ordinal variables as continuous variables. Tests of interaction between work schedule and chemical co-exposures, and between work schedule and menopausal status were conducted by including product terms of dichotomous variables in the models.

We had up to 39% missing in the covariates of the multivariable model. We used multiple imputation with chained equations, assuming missing at random [40], to impute 45 datasets. In each data set, we conducted analyses using the models described above, and estimates were combined using Rubin's rule [41]. We report results with and without imputed data. We have extended the description of the imputations in the supplements where we also tried an alternative imputation model (Supplemental Material, Methods extension, Table S3).

Tests for significance were two-sided, and P-values <0.05 were considered as evidence against the null hypothesis. Data analyses were performed using Stata version 16.1 (StataCorp, College Station, TX, USA).

**RESULTS**

Table 1 shows baseline characteristics of the 86 cases and 514 non-cases in the study sample. Mean age at baseline was 45 years for cases and 42 years for non-cases. Among the



For peer review only - http://bmjopen.bmj.com/site/about/guidelines.xhtml

cases and non-cases, mean employment duration was 8.4 and 6.9 years, the most frequent main occupational activity in last position was catering/office/administration (73% *vs.* 71%, respectively), most had day work only (55% *vs.* 53%), and 37% of the cases *vs.* 42% of the non-cases were exposed to night/rollover shift. Twenty-seven percent of the cases had >6 years of night/rollover shift compared with 18% of non-cases. Up to 68% of the cases and 77% of the non-cases may have been exposed to chlorinated degreasers. For benzene, 15% of the cases and 18% of the non-cases were exposed. Mean age at breast cancer diagnosis was 56 years, whereof 78% were ER-positive and 69% PR-positive, while 70% were HER2-negative (Supplemental Material, Table S4).

No interaction was found between work schedule and menopausal status when examining risk of overall or any breast cancer subtypes. The P-values of the interaction terms in the models were between 0.279 and 0.639, we therefore do not present results stratified by menopausal status. Results from complete-case and multiple imputation analyses were largely similar across all analyses (Tables 2-4), and we refer to the multiple imputation results in the following.

No association was found between breast cancer risk and total employment duration ($P_{trend}$=0.733) (Table 2). The HR of breast cancer in relation to work schedule ever involving night/rollover shift was 0.87 (95% CI: 0.52, 1.46) *vs.* never, and no significant association was found with duration of night/rollover shift ($P_{trend}$=0.748). Sensitivity analyses of duration of night/rollover shift by quartile among the exposed showed similar result ($P_{trend}$=0.944) (Supplemental Material, Table S5).

There was no significant association between main occupational activity in last position and breast cancer risk (Table 3). No significant associations with breast cancer risk were found for chlorinated degreaser or benzene exposure (HRs (95% CIs) 0.89 (0.52, 1.52) and 0.90 (0.46, 1.79), respectively, for ever *vs*. never, Table 3). No significant interaction was



For peer review only - http://bmjopen.bmj.com/site/about/guidelines.xhtml

found between work schedule and chemical co-exposures ($P_{interaction\ chlorinated\ degreasers}$=0.725; $P_{interaction\ benzene}$=0.175) (Supplemental Material, Table S6).

There was no significant association between work schedule and risk of any breast cancer subtypes. For the breast cancer subtypes that did not express sex hormone receptors we found non-significantly elevated risks for work schedule involving night/rollover shift compared to day work only (HR=1.53, 95% CI: 0.50, 4.72 for ER-negative tumours; HR=1.69, 95% CI: 0.58, 4.87 for PR-negative tumours; HR=2.22, 95% CI: 0.58, 8.51 for ER-negative and PR-negative and HER2-negative tumours) (Table 4).

**DISCUSSION**

In this study of prospectively-recruited female offshore workers, we observed no association between overall breast cancer risk and night/rollover shift, duration of such work, or total employment duration. Some indication of increased risks was observed for ER-negative and PR-negative breast cancer subtypes, but these findings were not statistically significant and were based on few observations. We did not find evidence for interaction between work schedule and chemical co-exposures in relation to risk of breast cancer.

The literature is inconclusive on the association between night shift work and breast cancer risk. A report from the Nurses' Health Study [9] found an elevated risk among nurses who had long (≥20 years) cumulative night shift work and started their career early, which is also in accordance with other studies [10, 11, 17]. One explanation for these positive associations between long-term night shift work and breast cancer risk may be that breast tissue is particularly susceptible to circadian disruption at a younger age [9]. However, in line with our main findings, more recent studies did not find an association between night shift work and breast cancer [13-15]. Importantly, the most recent meta-analysis, stratifying on short- and long-term night shift work, found an increased breast cancer risk related to short-term, but not long-term night shift work [42]. Possible reasons that may explain our negative



For peer review only - http://bmjopen.bmj.com/site/about/guidelines.xhtml

results are that, despite the extreme and unique work pattern, offshore workers have a four-week off-duty period following each standard two-week tour [6]. During the off-duty period, it is possible that workers could rest and readjust their circadian rhythm, and hence reduce the potential effect of night shift work on breast cancer risk. Further, the continuous shift work pattern (7–14 consecutive nights) of offshore workers might facilitate some adaptation and thereby represent a lower degree of circadian disruption, which in turn might reduce the risk of breast cancer [5].

To our knowledge, this is the first study of night shift work and co-exposure to chlorinated degreaser and benzene in relation to breast cancer risk. The hypothesis that night shift work may potentiate chemical exposure by desynchronising detoxification mechanisms [29] was not confirmed, as we did not find any statistically significant interactions, although it may be ascribed to lack of power and misclassification of exposure.

Some studies have investigated the relationship between night shift work and breast cancer subtypes. Night shift work has been associated with sex hormone receptor-positive (ER-positive or PR-positive) tumours [14, 17, 18, 20], which is in line with experimental evidence that melatonin suppression enhances proliferation of ER-positive cell lines [43]. Zhu et al. [44] suggested that melatonin disturbance could instigate two independent pathways that lead to both ER-positive and ER-negative tumours. This would be in line with our findings of increased risk of ER-negative tumours among night-shift workers, which are consistent with the study by Rabstein et al. [22].

Our study has several strengths. First, linkage to nationwide cancer and population registries ensured high quality cancer data and complete follow-up. Second, we had access to independent chemical exposure estimates developed by industrial hygiene experts specifically for cancer studies in our cohort. Third, offshore workers have extreme work schedules and the NOPW cohort therefore provides particularly interesting data to study the association with



For peer review only - http://bmjopen.bmj.com/site/about/guidelines.xhtml

breast cancer. Although, self-reported work history (*i.e.* exposure) was collected prior to the breast cancer diagnoses, and has been shown to be robust to recall errors [45], we cannot rule out some differential misclassification as a result of collapsing exposure categories [46, 47]. However, the results of analysis with categorical and continuous exposure gave similar results (Table 2). The females in the NOPW cohort were quite young at baseline (mean age 42), which may explain the relatively few cases. Also, the low case numbers in some exposure categories reduced the statistical power of detecting significant deviations from the null hypothesis. Comparing our results with the pooled-analysis of Cordina-Duverger *et al.* [17], which had larger sample size and reported a positive association between night shift work and breast cancer (all receptor subtypes combined), the two studies do not contradict each other (HR=0.87, 95% CI: 0.52, 1.46 in our study *vs.* OR=1.12, 95% CI: 1.00, 1.25 in Cordina-Duverger *et al.*). Our study had relatively few cases resulting in low power and wide confidence intervals. The limited power may leave real interactions undetected. In an attempt to reduce the impact of limited power, we tested interactions with binary variables (ever/never exposure). The probability-oriented JEM for chlorinated degreasers classified catering workers as 'possibly exposed' and assigned the same exposure level despite the fact that exposure may vary by job tasks. Hence, the actual fraction of workers exposed to chlorinated degreasers in this job-category is uncertain. We also lacked information on some potential confounders (*e.g.* chronotype, exogenous hormone use, family history and breast feeding history), and we had no information on exposures during follow-up.

In conclusion, our findings in this prospectively-recruited cohort need to be interpreted carefully due to limited statistical power, but pointed towards that night/rollover shift, exposure to chlorinated degreasers or to benzene may not be associated with increased breast cancer risk in female offshore petroleum workers. However, further studies with larger



For peer review only - http://bmjopen.bmj.com/site/about/guidelines.xhtml

sample sizes are warranted for analyses with combined exposures and for analyses of breast cancer by receptor subtype.

For peer review only



For peer review only - http://bmjopen.bmj.com/site/about/guidelines.xhtml

**FUNDING**

This work was supported by the Research Council of Norway grant number 280537.

**CONFLICTS OF INTEREST**

None declared. JSS and TKG have received a grant from the Research Council of Norway (governmental agency) that awarded an industry-collaborative grant to the Cancer Registry of Norway (governmental agency) in 2019 to establish a cohort of offshore petroleum workers. A condition pertaining to industry-collaborative grants is that 20% (175,000 USD) of the grant was provided by the petroleum industry and 80% (700,000 USD) by the Research Council itself with the intention of joining forces for the common interest of occupational health among petroleum workers. The application process was governed by the Research Council without any involvement from the industry. The grant does not cover the PI's or any of the collaborators salary.

**AUTHOR CONTRIBUTIONS**

JSS and TKG conceived the study. FCL, MBV, KK, TER, RG, HDH, SOS, MB, JK, NR, QL, DTS, MCF, RB, NKS, TKG and JSS contributed to the project design. FCL performed the data analyses. FCL, RB, and JSS performed the data management. KK contributed with their expertise on breast cancer epidemiology. MB and JK contributed to chemical exposure assessment. FCL drafted the manuscript and all authors reviewed and revised it critically for important intellectual content and approved the final version for submission. FCL and JSS are the guarantors.

For peer review only

## ETHICS APPROVAL

This study was performed at the CRN after legal and ethical approvals were obtained from the Norwegian Data Inspectorate, the Regional Committee for Medical Research Ethics (2018/1162), and the Norwegian Directorate of Health.

## DATA SHARING STATEMENT

The data that support the findings of this study are available from the CRN (cohort data and cancer data) and the National Population Register (death and emigration data) but restrictions apply to the availability of these data, which were used under license for the current study, and so are not publicly available. Requests for data sharing/case pooling for projects with necessary approvals and legal basis according to the EU General Data Protection Regulation (GDPR) may be directed to principal investigator Dr Tom K Grimsrud; email: tom.k.grimsrud@kreftregisteret.no

For peer review only



For peer review only - http://bmjopen.bmj.com/site/about/guidelines.xhtml

# References


1. Bray F, Ferlay J, Soerjomataram I, Siegel RL, Torre LA, Jemal A. Global Cancer Statistics 2018: Globocan Estimates of Incidence and Mortality Worldwide for 36 Cancers in 185 Countries. CA Cancer J Clin. 2018;68(6):394-424.
2. Bray F, McCarron P, Parkin DM. The Changing Global Patterns of Female Breast Cancer Incidence and Mortality. Breast Cancer Res. 2004;6(6):229.
3. Francies FZ, Hull R, Khanyile R, Dlamini Z. Breast Cancer in Low-Middle Income Countries: Abnormality in Splicing and Lack of Targeted Treatment Options. Am J Cancer Res. 2020;10(5):1568-91.
4. Hansen J. Risk of Breast Cancer After Night- and Shift Work: Current Evidence and Ongoing Studies in Denmark. Cancer Causes & Control. 2006;17(4):531-7.
5. Stevens RG, Davis S. The Melatonin Hypothesis: Electric Power and Breast Cancer. Environ Health Persp. 1996;104(Suppl 1):135-40.
6. Parkes K. Working Hours in the Offshore Petroleum Industry Current Knowledge and Research Needs Regarding Extended Work Periods and Shift Work Offshore. In: Conference On Work Time Arrangements in the Petroleum Industry, 2007: Norway: Petroleum Safety Authority; 2007.
7. ILO. C171 - Night Work Convention No. 171 (C171). [Webpage]. Geneva, Switzerland: International Labour Organization: [cited 2020.29.09]. Available from: https://www.ilo.org/dyn/normlex/en/f?p=NORMLEXPUB:12100:0::NO::P12100_INSTRUMENT_ID:312316
8. ILO. R178 - Night Work Recommendation No.178 (R178). [Webpage]. Geneva, Switzerland: International Labour Organization: [cited 2020.29.09]. Available from: https://www.ilo.org/dyn/normlex/en/f?p=NORMLEXPUB:12100:0::NO::P12100_ILO_CODE:R178
9. Wegrzyn LR, Tamimi RM, Rosner BA, Brown SB, Stevens RG, Eliassen AH, et al. Rotating Night-Shift Work and the Risk of Breast Cancer in the Nurses' Health Studies. Am J Epidemiol. 2017;186(5):532-40.
10. Åkerstedt T, Knutsson A, Narusyte J, Svedberg P, Kecklund G, Alexanderson K. Night Work and Breast Cancer in Women: a Swedish Cohort Study. BMJ Open. 2015;5(4):e008127.
11. Hansen J, Lassen CF. Nested Case–Control Study of Night Shift Work and Breast Cancer Risk Among Women in the Danish Military. Occup Environ Med. 2012;69(8):551-6.
12. Lie JS, Kjuus H, Zienolddiny S, Haugen A, Stevens RG, Kjærheim K. Night Work and Breast Cancer Risk Among Norwegian Nurses: Assessment by Different Exposure Metrics. Am J Epidemiol. 2011;173(11):1272-9.
13. Jones ME, Schoemaker MJ, McFadden EV, Wright LB, Johns LE, Swerdlow AJ. Night Shift Work and Risk of Breast Cancer in Women: The Generations Study Cohort. Br J Cancer. 2019;121(2):172-9.
14. Vistisen HT, Garde AH, Frydenberg M, Christiansen P, Hansen AM, Hansen J, et al. Short-Term Effects of Night Shift Work on Breast Cancer Risk: A Cohort Study of Payroll Data. Scand J Work Environ Health. 2017;43(1):59-67.
15. Travis RC, Balkwill A, Fensom GK, Appleby PN, Reeves GK, Wang XS, et al. Night Shift Work and Breast Cancer Incidence: Three Prospective Studies and Meta-Analysis of Published Studies. J Natl Cancer Inst. 2016;108(12):djw169.
16. Koppes LL, Geuskens GA, Pronk A, Vermeulen RC, de Vroome EM. Night Work and Breast Cancer Risk in a General Population Prospective Cohort Study in the Netherlands. Eur J Epidemiol. 2014;29(8):577-84.
17. Cordina-Duverger E, Menegaux F, Popa A, Rabstein S, Harth V, Pesch B, et al. Night Shift Work and Breast Cancer: A Pooled Analysis of Population-Based Case–Control Studies With Complete Work History. Eur J Epidemiol. 2018;33(4):369-79.
18. Cordina-Duverger E, Koudou Y, Truong T, Arveux P, Kerbrat P, Menegaux F, et al. Night Work and Breast Cancer Risk Defined by Human Epidermal Growth Factor Receptor-2 (HER2) and Hormone Receptor Status: A Population-Based Case-Control Study in France. Chronobiol Int. 2016;33(6):783-7.




For peer review only - http://bmjopen.bmj.com/site/about/guidelines.xhtml

19. Schernhammer ES, Laden F, Speizer FE, Willett WC, Hunter DJ, Kawachi I, et al. Rotating Night Shifts and Risk of Breast Cancer in Women Participating in the Nurses' Health Study. J Natl Cancer Inst. 2001;93(20):1563-8.
20. Lie JS, Kjuus H, Zienolddiny S, Haugen A, Kjærheim K. Breast Cancer Among Nurses: Is the Intensity of Night Work Related to Hormone Receptor Status? Am J Epidemiol. 2013;178(1):110-7.
21. Papantoniou K, Castaño-Vinyals G, Espinosa A, Aragonés N, Pérez-Gómez B, Ardanaz E, et al. Breast Cancer Risk and Night Shift Work in a Case–Control Study in a Spanish Population. Eur J Epidemiol. 2016;31(9):867-78.
22. Rabstein S, Harth V, Pesch B, Pallapies D, Lotz A, Justenhoven C, et al. Night Work and Breast Cancer Estrogen Receptor Status--Results From the German Genica Study. Scand J Work Environ Health. 2013;39(5):448-55.
23. IARC. Night Shift Work. IARC Monogr Identif Carcinog Hazards Hum. 2020;124:1-371.
24. Bratveit M, Kirkeleit J, Hollund BE, Moen BE. Biological Monitoring of Benzene Exposure for Process Operators during Ordinary Activity in the Upstream Petroleum Industry. Ann Occup Hyg. 2007;51(5):487-94.
25. Steinsvåg K, Bratveit M, Moen BE. Exposure to Carcinogens for Defined Job Categories in Norway's Offshore Petroleum Industry, 1970 to 2005. Occup Environ Med. 2007;64(4):250-8.
26. Garcia E, Hurley S, Nelson DO, Hertz A, Reynolds P. Hazardous Air Pollutants and Breast Cancer Risk in California Teachers: A Cohort Study. Environmental health : a global access science source. 2015;14(1):14.
27. Pedersen JE, Strandberg-Larsen K, Andersson M, Hansen J. Occupational Exposure to Specific Organic Solvents and Risk of Subtypes of Breast Cancer in a Large Population of Danish Women, 1964-2016. Occup Environ Med. 2020;oemed-2020-106865. doi: 10.1136/oemed-2020-106865. Online ahead of print.
28. Masri S, Sassone-Corsi P. The Emerging Link Between Cancer, Metabolism, and Circadian Rhythms. Nat Med 2018;24:1795-803.
29. Carmona-Antoñanzas G, Santi M, Migaud H, Vera LM. Light- and Clock-Control of Genes Involved in Detoxification. Chronobiol Int. 2017;34(8):1026-41.
30. Stenehjem JS, Babigumira R, Hosgood HD, Veierød MB, Samuelsen SO, Bråtveit M, et al. Cohort Profile: The Norwegian Offshore Petroleum Workers (NOPW) Cohort. Int J Epidemiol. 2020;dyaa107.
31. Stenehjem JS, Kjaerheim K, Rabanal KS, Grimsrud TK. Cancer Incidence Among 41,000 Offshore Oil Industry Workers. Occup Med (Lond). 2014;64(7):539-45.
32. Borgan O, Langholz B, Samuelsen SO, Goldstein L, Pogoda J. Exposure Stratified Case-Cohort Designs. Lifetime Data Anal. 2000;6(1):39-58.
33. Larsen IK, Smastuen M, Johannesen TB, Langmark F, Parkin DM, Bray F, et al. Data Quality at the Cancer Registry of Norway: An Overview of Comparability, Completeness, Validity and Timeliness. Eur J Cancer. 2009;45(7):1218-31.
34. Stenehjem JS, Babigumira R, Friesen MC, Grimsrud TK. Harmonizing Work History Data in Epidemiologic Studies With Overlapping Employment Records. Am J Ind Med. 2019;62(5):422-9.
35. Steinsvag K, Bratveit M, Moen BE, Kromhout H. Inter-Rater Agreement in the Assessment of Exposure to Carcinogens in the Offshore Petroleum Industry. Occup Environ Med. 2007;64(9):582-8.
36. Bråtveit M, Kirkeleit J, Hollund BE, Vågnes KS. Development of a Retrospective JEM for Benzene in the Norwegian Oil and Gas Industry. Occup Environ Med. 2011;68(Suppl 1):A26-A.
37. Beral V. Breast Cancer and Hormone-Replacement Therapy in the Million Women Study. Lancet. 2003;362(9382):419-27.
38. Busund M, Bugge NS, Braaten T, Waaseth M, Rylander C, Lund E. Progestin-Only and Combined Oral Contraceptives and Receptor-Defined Premenopausal Breast Cancer Risk: The Norwegian Women and Cancer Study. Int J Cancer. 2018;142(11):2293-302.
39. Bjelland EK, Hofvind S, Byberg L, Eskild A. The Relation of Age at Menarche with Age at Natural Menopause: A Population Study of 336 788 Women in Norway. Hum Reprod. 2018;33(6):1149-57.



For peer review only - http://bmjopen.bmj.com/site/about/guidelines.xhtml

40. Bartlett JW, Seaman SR, White IR, Carpenter JR. Multiple Imputation of Covariates by Fully Conditional Specification: Accommodating the Substantive Model. Stat Methods Med Res. 2015;24(4):462-87.
41. Rubin DB. Multiple Imputation for Nonresponse in Surveys. Hoboken, New Jersey: John Wiley & Sons; 2004. 131 p.
42. Manouchehri E, Taghipour A, Ghavami V, Ebadi A, Homaei F, Latifnejad Roudsari R. Night-Shift Work Duration and Breast Cancer Risk: An Updated Systematic Review and Meta-Analysis. BMC women's health. 2021;21(1):89.
43. Girgert R, Bartsch C, Hill SM, Kreienberg R, Hanf V. Tracking the Elusive Antiestrogenic Effect of Melatonin: A New Methodological Approach. Neuro Endocrinol Lett. 2003;24(6):440-4.
44. Zhu K, Bernard LJ, Levine RS, Williams SM. Estrogen Receptor Status of Breast Cancer: A Marker of Different Stages of Tumor or Different Entities of the Disease? Med Hypotheses. 1997;49(1):69-75.
45. Wärneryd B, Thorslund M, Ostlin P. The Quality of Retrospective Questions About Occupational History-a Comparison Between Survey and Census Data. Scand J Soc Med. 1991;19(1):7-13.
46. Wacholder S, Dosemeci M, Lubin JH. Blind Assignment of Exposure Does Not Always Prevent Differential Misclassification. Am J Epidemiol. 1991;134(4):433-7.
47. Jurek AM, Greenland S, Maldonado G, Church TR. Proper Interpretation of Non-Differential Misclassification Effects: Expectations vs Observations. Int J Epidemiol. 2005;34(3):680-7.

For peer review only

# TABLES

| Table 1. Baseline Characteristics of Females in the Norwegian Offshore Petroleum Workers (NOPW) Cohort. | | |
|---|---|---|
| **Variables** | **Cases (n=86)** | **Non-cases (n= 514)** |
| Age at baseline in 1998 (years), mean (range) | 45 (25-65) | 42 (19-74) |
| | | |
| **Anthropometric factors** | | |
| Height (cm), mean (range)[a] | 167 (155-184) | 167 (151-187) |
| Weight (kg), mean (range)[a] | 66 (49-95) | 66 (43-163) |
| BMI ($kg/m^2$), n (%) | | |
| 12-18.4 | 1 (1) | 13 (2) |
| 18.5-24.9 | 63 (73) | 343 (67) |
| 25.0-29.9 | 15 (18) | 121 (24) |
| ≥30.0 | 5 (6) | 29 (6) |
| Missing | 2 (2) | 8 (1) |
| | | |
| **Reproductive history** | | |
| Number of children, n (%) | | |
| 0 | 29 (34) | 151 (29) |
| 1 | 24 (28) | 85 (16) |
| 2 | 13 (15) | 147 (29) |
| ≥3 | 20 (23) | 123 (24) |
| Missing | 0 (0) | 8 (2) |
| Age at first child, mean (range)[a] | 26 (16-38) | 24 (16-41) |
| Postmenopausal at baseline, n (%)[b] | 18 (21) | 111 (22) |
| | | |
| **Social-demographic history** | | |
| Education, n (%) | | |
| Compulsory | 18 (21) | 106 (21) |
| Vocational | 17 (20) | 118 (23) |
| Upper secondary | 23 (27) | 148 (29) |
| University/college | 24 (27) | 138 (26) |
| Missing | 4 (5) | 4 (1) |
| | | |
| **Work history** | | |
| Main occupational activity in last position, n (%) | | |
| Production and process | 2 (2) | 23 (4) |
| Drilling and well maintenance | 3 (3) | 22 (4) |
| Maintenance/inspection/deck construction | 9 (11) | 59 (12) |
| Catering/office/administration | 63 (73) | 364 (71) |
| Miscellaneous | 9 (11) | 39 (8) |
| Missing | 0 (0) | 7 (1) |
| Total employment duration (years), mean (range) | 8.4 (0.3-20) | 6.9 (0.1-24) |
| Total employment duration quartile, n (%) | | |
| Quartile 1 (0-1.9 years) | 13 (15) | 124 (24) |
| Quartile 2 (2-5.9 years) | 23 (27) | 146 (28) |
| Quartile 3 (6-10.9 years) | 26 (30) | 121 (24) |
| Quartile 4 (11-24 years) | 24 (28) | 123 (24) |
| Work schedule, n (%) | | |
| Day work only | 47 (55) | 275 (53) |
| Mixed I (some night/rollover + mainly day) | 1 (1) | 18 (4) |
| Mixed II (mainly night/rollover + some day) | 31 (36) | 196 (38) |
| Missing | 7 (8) | 25 (5) |
| Duration by work schedule [a,c] | | |
| Day work (years), mean (range) | 6.4 (0.3-20) | 5.7 (0.1-21) |
| Night work (years), mean (range) | 3.6 (2.0-5.3) | 2.0 (0.1-11) |
| Rollover shift (years), mean (range) | 9.3 (1.0-18) | 6.8 (0.1-22) |
| Duration of night/rollover shift, n (%) | | |
| Unexposed (0 years, day work only) | 47 (55) | 275 (53) |
| ≤Median night/rollover (<1-6 years) [d] | 9 (10) | 121 (24) |
| >Median night/rollover (>6 years) [d] | 23 (27) | 93 (18) |
| Missing | 7 (8) | 25 (5) |
| | | |
| **Chemical co-exposure** | | |
| Chlorinated degreasers duration, n (%) [e] | | |
| Unexposed (0 years) | 28 (32) | 121 (23) |



For peer review only - http://bmjopen.bmj.com/site/about/guidelines.xhtml

| | | |
|---|---|---|
| ≤Median (<1–5 years) | 22 (26) | 214 (42) |
| >Median (6–23 years) | 36 (42) | 179 (35) |
| Benzene duration, n (%) | | |
| Unexposed (0 years) | 73 (85) | 424 (82) |
| Exposed (<1–23 years) | 13 (15) | 90 (18) |

[a] Missing in continuous variables: Height (n=8); Weight (n=9); Age at first child (n=193); Day work duration (n=32); Night work duration (n=32); Rollover shift duration (n=32); Night + Rollover shift duration (n=32)

[b] Postmenopause assumed at ≥ 53 years of age

[c] Workers with complete missing information on day, night and rollover (n=32) were excluded when calculating the mean

[d] Workers with day work mainly but also worked night/rollover shift were included in this category

[e] Importantly, the exposure assessment of chlorinated degreasers was probability-based by experts and evaluated as "possible" for catering workers.

For peer review only



For peer review only - http://bmjopen.bmj.com/site/about/guidelines.xhtml

| **Table 2**. Hazard Ratios of Female Breast Cancer According to Work Schedule in the Norwegian Offshore Petroleum Workers (NOPW) Cohort | | | | |
|---|---|---|---|---|
| | **Complete case analysis (n=367)** | | | **Multiple imputation (n=600; 86 cases)** |
| **Work schedule variable** | **No. of participants** | **No. of cases** | **HR (95% CI)** | **HR (95% CI)** |
| Total employment duration [a,b] | | | | |
| Quartile 1 (0-1.9 years) | 79 | 9 | 1.00 (reference) | 1.00 (reference) |
| Quartile 2 (2-5.9 years) | 99 | 16 | 1.59 (0.58, 4.35) | 1.50 (0.69, 3.27) |
| Quartile 3 (6-10.9 years) | 90 | 8 | 0.68 (0.24, 1.94) | 1.64 (0.78, 3.50) |
| Quartile 4 (11-24 years) | 99 | 16 | 1.21 (0.46, 3.13) | 1.19 (0.54, 2.61) |
| P-trend [c1] | | | 0.864 | 0.733 |
| Work schedule involving night/rollover shift [a,b] | | | | |
| Unexposed (day work only) | 217 | 29 | 1.00 (reference) | 1.00 (reference) |
| Exposed (night/rollover shift) | 150 | 20 | 1.06 (0.57, 1.97) | 0.87 (0.52, 1.46) |
| | | | | |
| Duration of night/rollover shift [a,b] | | | | |
| Unexposed (0 years, day work only) | 217 | 29 | 1.00 (reference) | 1.00 (reference) |
| ≤Median night/rollover (<1-6 years) | 68 | 6 | 0.72 (0.28, 1.86) | 0.56 (0.26, 1.22) |
| >Median night/rollover (>6 years) | 82 | 14 | 1.34 (0.67, 2.72) | 1.21 (0.67, 2.18) |
| P-trend [c2] | | | 0.523 | 0.748 |

Abbreviations: No=number, HR=hazard ratio, CI=confidence interval

[a] Adjusted for age, age at first child, number of children, menopause status, and education

[b] complete work history i.e. up to 8 employments as an offshore worker

[c1] Modelled as a continuous variable to test for linear trend, HR-complete case 0.97 (95% CI: 0.72, 1.31) and HR-imputed 1.04 (95% CI: 0.83, 1.30).

[c2] Modelled as a continuous variable to test for linear trend, HR-complete case 1.13 (95% CI: 0.78, 1.63) and HR-imputed 1.05 (95% CI: 0.77, 1.44).

For peer review only



For peer review only - http://bmjopen.bmj.com/site/about/guidelines.xhtml

**Table 3**. Hazard Ratios of Female Breast Cancer According to Chemical Co-Exposure in the Norwegian Offshore Petroleum Workers (NOPW) Cohort

| | Complete case analysis (n=367) | | | Multiple imputation (n=600; 86 cases) |
|---|---|---|---|---|
| **Chemical exposure variable** | **No. of participants** | **No. of cases** | **HR (95% CI)** | **HR (95% CI)** |
| Main occupational activity in last position [a] | | | | |
| Production/drilling/maintenance | 58 | 8 | 1.17 (0.47, 2.88) | 0.92 (0.48, 1.79) |
| Catering/office/administration | 280 | 35 | 1.00 (reference) | 1.00 (reference) |
| Miscellaneous | 29 | 6 | 1.70 (0.63, 4.53) | 1.19 (0.54, 2.63) |
| | | | | |
| Chlorinated degreasers [a,b] | | | | |
| Never | 95 | 17 | 1.00 (reference) | 1.00 (reference) |
| Ever | 272 | 32 | 0.72 (0.35, 1.48) | 0.89 (0.52, 1.52) |
| | | | | |
| Benzene [a] | | | | |
| Never | 309 | 40 | 1.00 (reference) | 1.00 (reference) |
| Ever | 58 | 9 | 1.54 (0.65, 3.64) | 0.90 (0.46, 1.79) |

Abbreviations: No=number, HR=hazard ratio, CI=confidence interval

[a] Adjusted for age and education

[b] Importantly, the exposure assessment of chlorinated degreasers was probability-based by experts and evaluated as “possible” for catering workers.



For peer review only - http://bmjopen.bmj.com/site/about/guidelines.xhtml

**Table 4** Hazard Ratios of Female Breast Cancer by Receptor Status Subtypes in Relation to Work Schedule in the Norwegian Offshore Petroleum Workers (NOPW) Cohort

| | | **Complete case analysis (n=367)** | | | **Multiple imputation (n=600; 86 cases)** |
|---|---|---|---|---|---|
| **Breast cancer subtype** | **Work schedule variable** | **No. of participants** | **No. of cases** | **HR (95% CI)** | **HR (95% CI)** |
| ER-positive | Work schedule involving night/rollover shift [a] | | | | |
| | Unexposed (day work only) | 217 | 23 | 1.00 (reference) | 1.00 (reference) |
| | Exposed (night/rollover shift) | 150 | 16 | 1.00 (0.50, 2.00) | 0.75 (0.41, 1.36) |
| ER-negative | Work schedule involving night/rollover shift [a] | | | | |
| | Unexposed (day work only) | 217 | 5 | 1.00 (reference) | 1.00 (reference) |
| | Exposed (night/rollover shift) | 150 | 2 | 0.78 (0.14, 4.46) | 1.53 (0.50, 4.72) |
| PR-positive | Work schedule involving night/rollover shift [a] | | | | |
| | Unexposed (day work only) | 217 | 25 | 1.00 (reference) | 1.00 (reference) |
| | Exposed (night/rollover shift) | 150 | 13 | 0.80 (0.39, 1.68) | 0.67 (0.36, 1.26) |
| PR-negative | Work schedule involving night/rollover shift [a] | | | | |
| | Unexposed (day work only) | 217 | 3 | 1.00 (reference) | 1.00 (reference) |
| | Exposed (night/rollover shift) | 150 | 5 | 2.10 (0.55, 8.08) | 1.69 (0.58, 4.87) |
| HER2-positive | Work schedule involving night/rollover shift [a] | | | | |
| | Unexposed (day work only) | 217 | 7 | 1.00 (reference) | 1.00 (reference) |
| | Exposed (night/rollover shift) | 150 | 1 | 0.29 (0.04, 2.14) | 0.22 (0.05, 0.99) |
| HER2-negative | Work schedule involving night/rollover shift [a] | | | | |
| | Unexposed (day work only) | 217 | 21 | 1.00 (reference) | 1.00 (reference) |
| | Exposed (night/rollover shift) | 150 | 14 | 1.02 (0.49, 2.15) | 0.88 (0.47, 1.62) |
| ER-positive and PR-positive and HER2-negative | Work schedule involving night/rollover shift [a] | | | | |
| | Unexposed (day work only) | 217 | 17 | 1.00 (reference) | 1.00 (reference) |
| | Exposed (night/rollover shift) | 150 | 9 | 0.79 (0.32, 1.95) | 0.58 (0.26, 1.28) |
| ER-positive and PR-positive and HER2-positive | Work schedule involving night/rollover shift [a] | | | | |
| | Unexposed (day work only) | 217 | 5 | 1.00 (reference) | 1.00 (reference) |
| | Exposed (night/rollover shift) | 150 | 1 | 0.38 (0.05, 2.70) | 0.29 (0.06, 1.29) |
| ER-negative and PR-negative and HER2-positive | Work schedule involving night/rollover shift [a] | | | | |
| | Unexposed (day work only) | 217 | 0 | 1.00 (reference) | 1.00 (reference) |
| | Exposed (night/rollover shift) | 150 | 0 | - | - |
| ER-negative and PR-negative and HER2-negative | Work schedule involving night/rollover shift [a] | | | | |
| | Unexposed (day work only) | 217 | 3 | 1.00 (reference) | 1.00 (reference) |
| | Exposed (night/rollover shift) | 150 | 2 | 1.36 (0.16, 11) | 2.22 (0.58, 8.51) |

Abbreviations: No=number, HR=hazard ratio, CI=confidence interval, ER=oestrogen receptor, PR=progesterone receptor, HER2=human epidermal growth factor receptor 2

[a] Adjusted for age, age at first child, number of children, menopause status, and education



For peer review only - http://bmjopen.bmj.com/site/about/guidelines.xhtml

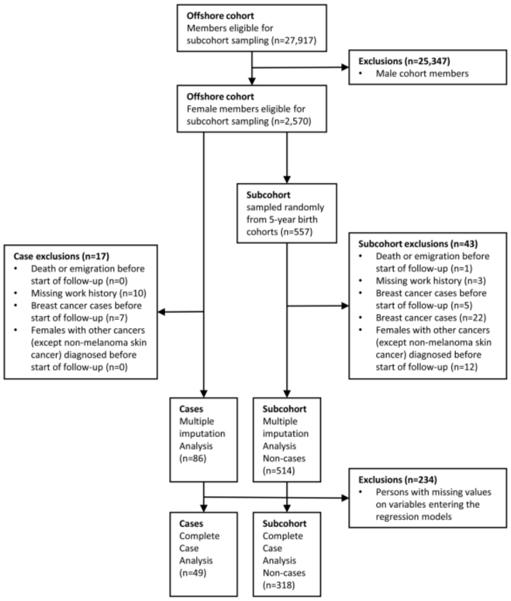


Figure 1. Overview of study design and exclusions.

275x190mm (300 x 300 DPI)

**SUPPLEMENTAL MATERIAL**

**Content:**

- Methods’ extension on multiple imputations
- Table S1
- Table S2
- Table S3
- Table S4
- Table S5
- Table S6
- Directed acyclic graphs (DAGs)
    - Figure S1
    - Figure S2

For peer review only



For peer review only - http://bmjopen.bmj.com/site/about/guidelines.xhtml

**Methods' extension**

Multiple imputations

*Complete case analysis (n=367)*

As shown in Figure 1, the study sample for our complete case analysis included 49 breast cancer cases (with complete information on all variables in the regression model).

*Analysis of imputed data (n=600)*

As shown in Figure 1, our study sample yielded 86 breast cancer cases when those missing data retained. In this sample, 39% (n=233) had missing data on one or more of the following variables (missing numbers are displayed per variable):

- Work schedule involving night/rollover shift: n=32
- Duration of night/rollover shift: n=32
- Number of children: n=8
- Age at first child: n=192
- Education: n=8
- Main occupational activity in last position: n=7

Assuming missing at random, we used multiple imputation with chained equations and followed a 2-steps procedure:

1. We identified differences between these samples for date of birth, total employment duration, and breast cancer diagnosis, and used these variables as predictors together with the variables from the estimation model (i.e. work schedule involving night/rollover shift, duration of night/rollover shift, number of children, age at first child, education, and main



For peer review only - http://bmjopen.bmj.com/site/about/guidelines.xhtml

occupational activity in last position) to simultaneously predict missing data on all variables. Linear and multinomial logit models were used to predict missing data for continuous and categorical variables, respectively. The imputation model was ran 45 times, using the rule of thumb of running at least as many times as the percentage of missing data (White et al., 2011).

2. After step 1, we replicated the complete case analyses shown in Table 2, Table 3, and Table 4 on the imputed dataset (n=600).

In a sensitivity analysis (Table S4), we also tried an alternative imputation model where we excluded the variable 'age at first child', which had the highest number of missing (n=192, 39%). This reduced the total number of missing to n=53 (9%), but did not changed the results materially (Table S4).

Reference

White IR, Royston P, Wood AM. Multiple imputation using chained equations: Issues and guidance for practice. Statistics in medicine 2011; 30: 377-99.



For peer review only - http://bmjopen.bmj.com/site/about/guidelines.xhtml

# Table S1

| **Table S1**. Hazard Ratios of Female Breast Cancer According to Work Schedule in the Norwegian Offshore Petroleum Workers (NOPW) Cohort, sensitivity analyses with menopause cut-off at age ≥51 | | | | |
|---|---|---|---|---|
| | **Complete case analysis (n=367)** | | | **Multiple imputation (n=600; 86 cases)** |
| **Work schedule variable** | **No. of participants** | **No. of cases** | **HR (95% CI)** | **HR (95% CI)** |
| Total employment duration [a,b] | | | | |
| Quartile 1 (0-1.9 years) | 79 | 9 | 1.00 (reference) | 1.00 (reference) |
| Quartile 2 (2-5.9 years) | 99 | 16 | 1.62 (0.59, 4.42) | 1.48 (0.68, 3.23) |
| Quartile 3 (6-10.9 years) | 90 | 8 | 0.69 (0.24, 2.00) | 1.61 (0.77, 3.38) |
| Quartile 4 (11-24 years) | 99 | 16 | 1.19 (0.46, 3.04) | 1.17 (0.54, 2.55) |
| P-trend [c] | | | 0.834 | 0.765 |
| Work schedule involving night/rollover shift [a,b] | | | | |
| Unexposed (day work only) | 217 | 29 | 1.00 (reference) | 1.00 (reference) |
| Exposed (night/rollover shift) | 150 | 20 | 1.05 (0.57, 1.96) | 0.86 (0.51, 1.45) |
| | | | | |
| Duration of night/rollover shift [a,b] | | | | |
| Unexposed (0 years, day work only) | 217 | 29 | 1.00 (reference) | 1.00 (reference) |
| ≤Median night/rollover (<1-6 years) | 68 | 6 | 0.71 (0.27, 1.86) | 0.55 (0.25, 1.21) |
| >Median night/rollover (>6 years) | 82 | 14 | 1.33 (0.66, 2.68) | 1.21 (0.67, 2.18) |
| P-trend [c] | | | 0.543 | 0.753 |
| Abbreviations: No=number, HR=hazard ratio, CI=confidence interval | | | | |
| [a] Adjusted for age, age at first child, number of children, menopause status (≥51 years as cut-off), and education | | | | |
| [b] complete work history i.e. up to 8 employments as an offshore worker | | | | |
| [c] Modelled as a continuous variable to test for linear trend. | | | | |

For peer review only

# Table S2

| Table S2. Hazard Ratios of Female Breast Cancer by Receptor Status Subtypes in Relation to Work Schedule in the Norwegian Offshore Petroleum Workers (NOPW) Cohort, sensitivity analyses with menopause cut-off at age ≥51 | | | | | |
|---|---|---|---|---|---|
| | | **Complete case analysis (n=367)** | | | **Multiple imputation (n=600; 86 cases)** |
| **Breast cancer subtype** | **Work schedule variable** | **No. of participants** | **No. of cases** | **HR (95% CI)** | **HR (95% CI)** |
| ER-positive | Work schedule involving night/rollover shift [a] | | | | |
| | Unexposed (day work only) | 217 | 23 | 1.00 (reference) | 1.00 (reference) |
| | Exposed (night/rollover shift) | 150 | 16 | 1.00 (0.50, 2.01) | 0.74 (0.41, 1.34) |
| ER-negative | Work schedule involving night/rollover shift [a] | | | | |
| | Unexposed (day work only) | 217 | 5 | 1.00 (reference) | 1.00 (reference) |
| | Exposed (night/rollover shift) | 150 | 2 | 0.76 (0.14, 4.17) | 1.53 (0.50, 4.70) |
| PR-positive | Work schedule involving night/rollover shift [a] | | | | |
| | Unexposed (day work only) | 217 | 25 | 1.00 (reference) | 1.00 (reference) |
| | Exposed (night/rollover shift) | 150 | 13 | 0.79 (0.38, 1.64) | 0.66 (0.35, 1.24) |
| PR-negative | Work schedule involving night/rollover shift [a] | | | | |
| | Unexposed (day work only) | 217 | 3 | 1.00 (reference) | 1.00 (reference) |
| | Exposed (night/rollover shift) | 150 | 5 | 2.08 (0.55, 7.88) | 1.72 (0.59, 5.02) |
| HER2-positive | Work schedule involving night/rollover shift [a] | | | | |
| | Unexposed (day work only) | 217 | 7 | 1.00 (reference) | 1.00 (reference) |
| | Exposed (night/rollover shift) | 150 | 1 | 0.27 (0.04, 1.91) | 0.21 (0.05, 0.98) |
| HER2-negative | Work schedule involving night/rollover shift [a] | | | | |
| | Unexposed (day work only) | 217 | 21 | 1.00 (reference) | 1.00 (reference) |
| | Exposed (night/rollover shift) | 150 | 14 | 1.04 (0.50, 2.16) | 0.88 (0.48, 1.62) |
| ER-positive and PR-positive and HER2-negative | Work schedule involving night/rollover shift [a] | | | | |
| | Unexposed (day work only) | 217 | 17 | 1.00 (reference) | 1.00 (reference) |
| | Exposed (night/rollover shift) | 150 | 9 | 0.80 (0.33, 1.94) | 0.58 (0.26, 1.28) |
| ER-positive and PR-positive and HER2-positive | Work schedule involving night/rollover shift [a] | | | | |
| | Unexposed (day work only) | 217 | 5 | 1.00 (reference) | 1.00 (reference) |
| | Exposed (night/rollover shift) | 150 | 1 | 0.35 (0.05, 2.36) | 0.27 (0.06, 1.26) |
| ER-negative and PR-negative and HER2-positive | Work schedule involving night/rollover shift [a] | | | | |
| | Unexposed (day work only) | 217 | 0 | 1.00 (reference) | 1.00 (reference) |
| | Exposed (night/rollover shift) | 150 | 0 | - | - |
| ER-negative and PR-negative and HER2-negative | Work schedule involving night/rollover shift [a] | | | | |
| | Unexposed (day work only) | 217 | 3 | 1.00 (reference) | 1.00 (reference) |
| | Exposed (night/rollover shift) | 150 | 2 | 1.34 (0.17, 11) | 2.22 (0.58, 8.45) |
| Abbreviations: No=number, HR=hazard ratio, CI=confidence interval, ER=oestrogen receptor, PR=progesterone receptor, HER2=human epidermal growth factor receptor 2 | | | | | |
| [a] Adjusted for age, age at first child, number of children, menopause status (≥51 years as cut-off), and education | | | | | |



For peer review only - http://bmjopen.bmj.com/site/about/guidelines.xhtml

# Table S3

**Table S3**. Hazard Ratios of Female Breast Cancer According to Work Schedule in the Norwegian Offshore Petroleum Workers (NOPW) Cohort. Sensitivity Analyses of models adjusted for age at first child *versus* models without adjustment for age at first child.

| | **Multiple imputation, including 'age at first child' (n=600; 86 cases)** [a,b] | **Multiple imputation, excluding 'age at first child' (n=600; 86 cases)** [b,c] |
|---|---|---|
| **Work schedule variable** | **HR (95% CI)** | **HR (95% CI)** |
| Total employment duration | | |
| Quartile 1 (0-1.9 years) | 1.00 (reference) | 1.00 (reference) |
| Quartile 2 (2-5.9 years) | 1.50 (0.69, 3.27) | 1.53 (0.71, 3.31) |
| Quartile 3 (6-10.9 years) | 1.64 (0.78, 3.50) | 1.65 (0.78, 3.47) |
| Quartile 4 (11-24 years) | 1.19 (0.54, 2.61) | 1.22 (0.56, 2.67) |
| P-trend [d1] | 0.733 | 0.685 |
| Work schedule involving night/rollover shift | | |
| Unexposed (day work only) | 1.00 (reference) | 1.00 (reference) |
| Exposed (night/rollover shift) | 0.87 (0.52, 1.46) | 0.87 (0.52, 1.45) |
| | | |
| Duration of night/rollover shift | | |
| Unexposed (0 years, day work only) | 1.00 (reference) | 1.00 (reference) |
| ≤Median night/rollover (<1-6 years) | 0.56 (0.26, 1.22) | 0.55 (0.25, 1.19) |
| >Median night/rollover (>6 years) | 1.21 (0.67, 2.18) | 1.21 (0.67, 2.17) |
| P-trend [d2] | 0.748 | 0.752 |

Abbreviations: HR=hazard ratio, CI=confidence interval

[a] Adjusted for age, age at first child, number of children, menopause status (≥53 years as cut-off), and education. 39% (n=233) missing data imputed.

[b] complete work history i.e. up to 8 employments as an offshore worker

[c] Adjusted for variable in footnote "a" but without age at first child. 9% (n=53) missing data imputed.

[d1] Modelled as a continuous variable to test for linear trend. HR for excluding 'age at first child' 1.05 (95% CI: 0.84-1.31).

[d2] Modelled as a continuous variable to test for linear trend. HR for excluding 'age at first child' 1.05 (95% CI: 0.77-1.44).

For peer review only



For peer review only - http://bmjopen.bmj.com/site/about/guidelines.xhtml

# Table S4

| **Table S4.** Characteristics of Female Breast Cancer Cases in the Norwegian Offshore Petroleum Workers (NOPW) Cohort. | |
|---|---|
| | **Breast Cancer Cases [a] (n=86)** |
| Age at diagnosis, mean (range) | 56 (38-80) |
| Clinical stage, n (%) | |
| I: No metastasis | 38 (44) |
| II: Regional lymph node metastasis | 29 (34) |
| III: Local infiltration into skin | 12 (14) |
| IV: Distant metastasis | 4 (5) |
| Unspecified | 3 (3) |
| Oestrogen receptors (ER), n (%) | |
| ER-positive | 67 (78) |
| ER-negative | 13 (15) |
| Missing | 6 (7) |
| Progesterone receptors (PR), n (%) | |
| PR-positive | 59 (69) |
| PR-negative | 21 (24) |
| Missing | 6 (7) |
| Human epidermal growth factor receptor-2 (HER2), n (%) | |
| HER2-positive | 12 (14) |
| HER2-negative | 60 (70) |
| Missing | 14 (16) |
| ER-positive and PR-positive and HER2-negative, n (%) | |
| Yes | 39 (45) |
| No | 30 (35) |
| Missing | 17 (20) |
| ER-positive and PR-positive and HER2-positive, n (%) | |
| Yes | 9 (10) |
| No | 60 (70) |
| Missing | 17 (20) |
| ER-negative and PR-negative and HER2-positive, n (%) | |
| Yes | 0 (0) |
| No | 69 (80) |
| Missing | 17 (20) |
| ER-negative and PR-negative and HER2-negative | |
| Yes | 11 (13) |
| No | 58 (67) |
| Missing | 17 (20) |

[a] According to International Classification of Diseases 10th edition : C50



For peer review only - http://bmjopen.bmj.com/site/about/guidelines.xhtml

# Table S5

| **Table S5**. Hazard Ratios of Breast Cancer According to duration of night + rollover shift quartile in the Norwegian Offshore Petroleum Workers (NOPW) Cohort | | | | |
|---|---|---|---|---|
| | **Complete case analysis (n=367)** | | | **Multiple imputation (n=600; 86 cases)** |
| **Work schedule variable** | **No. of participants** | **No. of cases** | **HR (95% CI)** | **HR (95% CI)** |
| Duration of night/rollover shift [a,b] | | | | |
| Unexposed (0 years, day work only) | 217 | 29 | 1.00 (reference) | 1.00 (reference) |
| Quartile 1 night/rollover (<1-2 years) | 28 | 3 | 0.99 (0.28, 3.52) | 0.78 (0.28, 2.14) |
| Quartile 2 night/rollover (>2-5 years) | 32 | 2 | 0.52 (0.11, 2.47) | 0.49 (0.14, 1.71) |
| Quartile 3 night/rollover (>5-10 years) | 36 | 4 | 0.77 (0.24, 2.48) | 1.02 (0.47, 2.24) |
| Quartile 4 night/rollover (>10 years) | 54 | 11 | 1.62 (0.73, 3.59) | 1.10 (0.51, 2.37) |
| P-trend [c] | | | 0.506 | 0.944 |
| Abbreviations: No=number, HR=hazard ratio, CI=confidence interval | | | | |
| [a] Adjusted for age, age at first child, number of children, menopause status (≥53 years as cut-off), and education | | | | |
| [b] complete work history i.e. up to 8 employments as an offshore worker | | | | |
| [c] Modelled as a continuous variable to test for linear trend | | | | |

For peer review only

# Table S6

**Table S6**. Hazard Ratios of Breast Cancer According to Work Schedule and Chemical Co-exposures in the Norwegian Offshore Petroleum Workers (NOPW) Cohort, multiple imputation analysis (n=601)

| | **Chlorinated degreasers [a]** | | |
|---|---|---|---|
| **Work schedule variable [b,d]** | **Unexposed** | **Exposed** | **P Value** |
| Unexposed (day work only) | | | |
| n (%) | 99 (17) | 223 (39) | |
| HR [c] (95% CI) | 1.00 (reference) | 0.91 (0.45, 1.84) | |
| Exposed (night/rollover shift) | | | |
| n (%) | 43 (8) | 204 (36) | |
| HR [c] (95% CI) | 1.01 (0.40, 2.55) | 0.79 (0.37, 1.71) | |
| $P_{interaction}$ | | | 0.725 |
| | **Benzene** | | |
| **Work schedule variable [b,d]** | **Unexposed** | **Exposed** | **P Value** |
| Unexposed (day work only) | | | |
| n (%) | 269 (48) | 53 (9) | |
| HR [c] (95% CI) | 1.00 (reference) | 0.60 (0.21, 1.70) | |
| Exposed (night/rollover shift) | | | |
| n (%) | 199 (35) | 48 (8) | |
| HR [c] (95% CI) | 0.74 (0.41, 1.32) | 1.11 (0.46, 2.70) | |
| $P_{interaction}$ | | | 0.175 |

Abbreviations: HR=hazard ratio, CI=confidence interval

[a] Importantly, the exposure assessment of chlorinated degreasers was probability-based by experts and evaluated as “possible” for catering workers.

[b] Complete work history i.e. up to 8 employments as an offshore worker

[c] Adjusted for age, age at first child, number of children, menopause status, and education

[d] Missing: n=32 for both chlorinated degreasers and benzene.



For peer review only - http://bmjopen.bmj.com/site/about/guidelines.xhtml

**Directed acyclic graphs (DAGs)**

DAGs were produced by the DAGitty v3.0 software (Textor et al., 2016). Please note that the green circle denotes the exposure, the blue circle with I denotes the outcome, the red circles denote the ancestor of the exposure AND the outcome (i.e. confounders, which are included in the models), and blue circles denote ancestor of the outcome (not included in the models).

Reference
Textor J, Zander B, Gilthorpe MK, Liskiewicz M, Ellison G. Robust causal inference using directed acyclic graphs: the R package 'dagitty'. Int J Epidemiol. 2016;45(6):1887-1894.
Website: www.dagitty.net

Models estimating breast cancer risk in relation to work schedule

In the following, we show DAG for each model with breast cancer as the outcome (as displayed in Tables 2 and 4) in relation to work schedule, age, menopausal status, age at first child, number of children, and education. We made the following assumptions:

- Age and education were not caused by any of the other variables in the DAG
- Menopausal status was affected by age
- Age at first child was affected by education
- Number of children was affected by age at first child



For peer review only - http://bmjopen.bmj.com/site/about/guidelines.xhtml

**Figure S1**

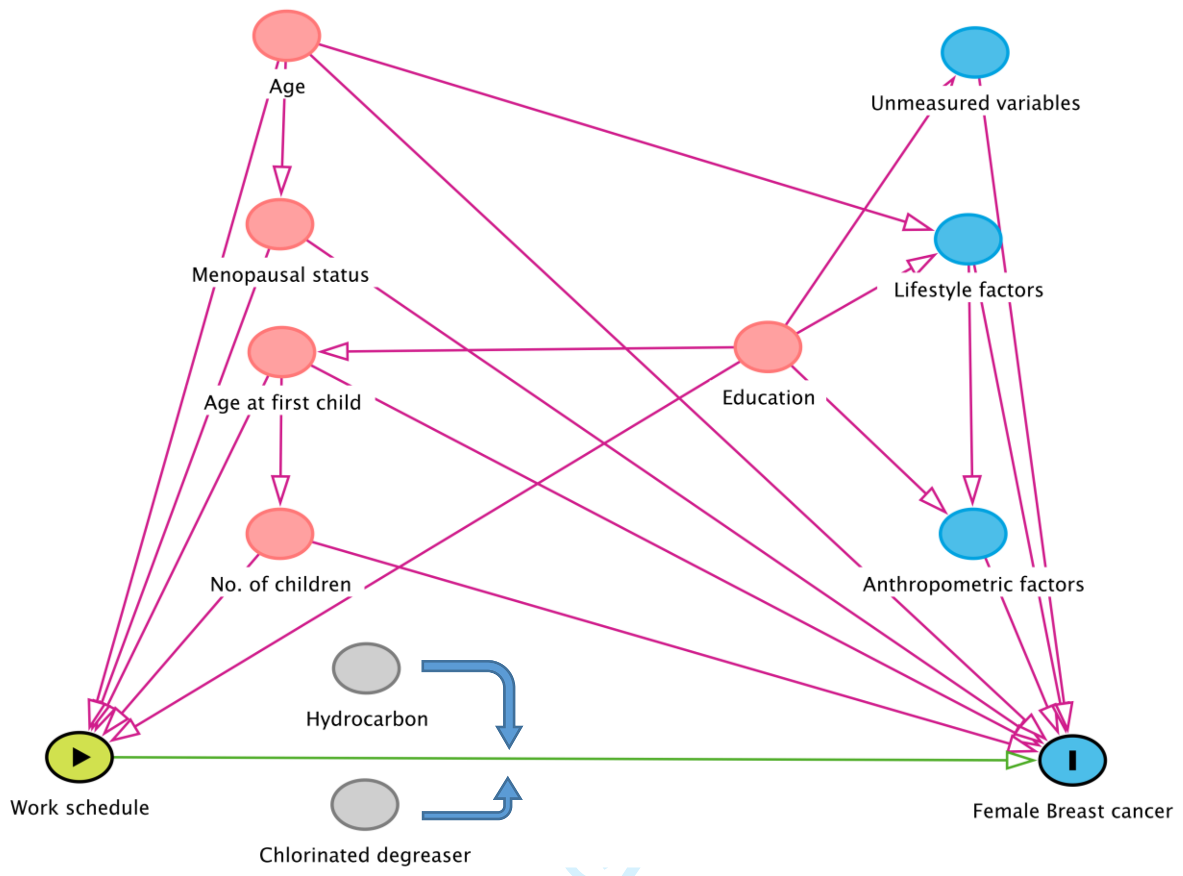


| Lifestyle factors: | Antropometric factors: | Unmeasured variables: |
|---|---|---|
| Fibre intake | Height | Chronotype |
| Multivitamine intake | Weight | Exogenous hormones use |
| Dietary fat intake | | Breast feeding |
| Alcohol intake | | Family history |
| Smoking | | |
| Physical activity | | |



For peer review only - http://bmjopen.bmj.com/site/about/guidelines.xhtml

Models estimating breast cancer risk in relation to chemical co-exposures

In the following, we show DAG for each model with breast cancer as the outcome (as displayed in Tables 3) in relation to age, education, main occupational activity in last position. We made the following assumptions:

- Age, and education were not caused by any of the other variables in the DAG
- Main occupational activity in last position was affected by education, and is an instrument of chemical co-exposure.

For peer review only



For peer review only - http://bmjopen.bmj.com/site/about/guidelines.xhtml

**Figure S2**

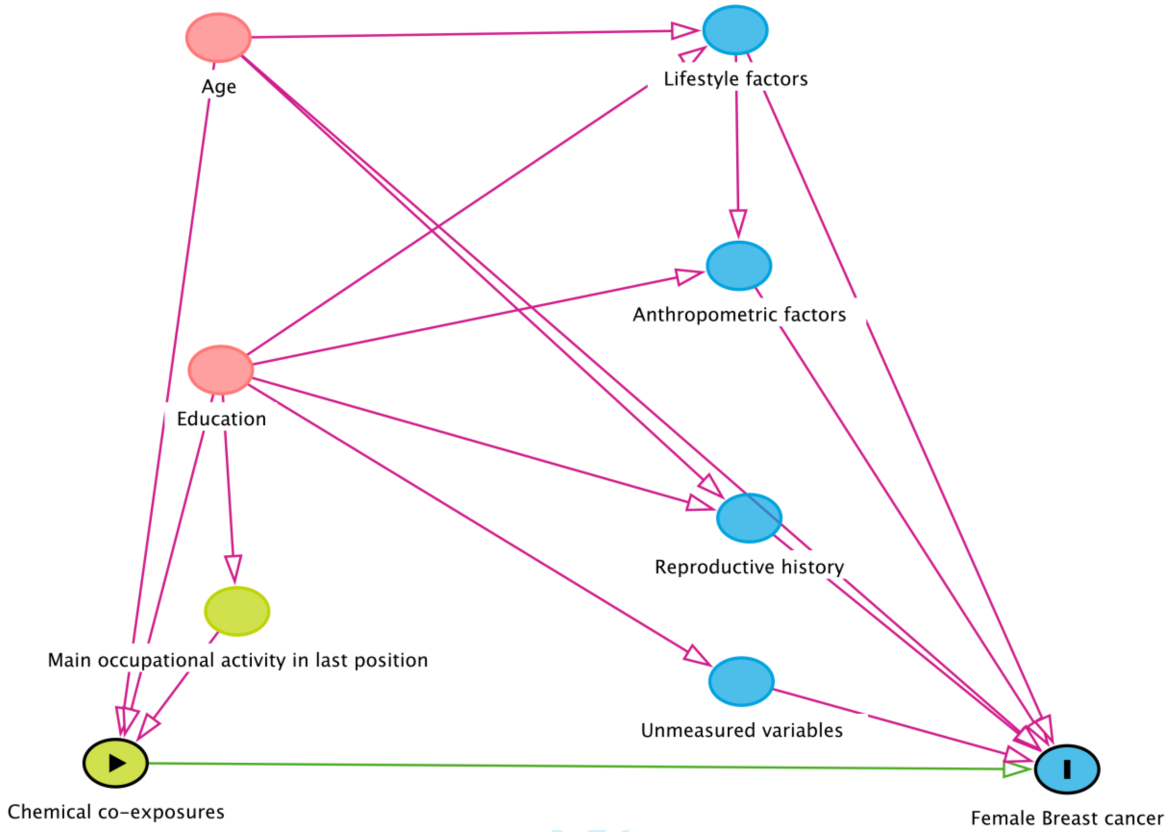


| **Chemical co-exposures**: | **Lifestyle factors:** | **Antropometric factors:** | **Reproductive history:** | **Unmeasured variables:** |
|---|---|---|---|---|
| Chlorinated degreaser | Fibre intake | Height | Age at first child | Exogenous hormones use |
| Benzene | Multivitamine intake | Weight | Number of children | Breast feeding |
| | Dietary fat intake | | Menopausal status | Family history |
| | Alcohol intake | | | |
| | Smoking | | | |
| | Physical activity | | | |



For peer review only - http://bmjopen.bmj.com/site/about/guidelines.xhtml

STROBE Statement—Checklist of items that should be included in reports of ***cohort studies***

| | Item No | Recommendation | Page No |
|---|---|---|---|
| **Title and abstract** | 1 | (*a*) Indicate the study's design with a commonly used term in the title or the abstract<br>(*b*) Provide in the abstract an informative and balanced summary of what was done and what was found | p. 1-2 |
| **Introduction** | | | |
| Background/rationale | 2 | Explain the scientific background and rationale for the investigation being reported | p. 4-5 |
| Objectives | 3 | State specific objectives, including any prespecified hypotheses | p. 5 |
| **Methods** | | | |
| Study design | 4 | Present key elements of study design early in the paper | p. 6 |
| Setting | 5 | Describe the setting, locations, and relevant dates, including periods of recruitment, exposure, follow-up, and data collection | p. 5-6 |
| Participants | 6 | (*a*) Give the eligibility criteria, and the sources and methods of selection of participants. Describe methods of follow-up<br>(*b*) For matched studies, give matching criteria and number of exposed and unexposed | p. 7 |
| Variables | 7 | Clearly define all outcomes, exposures, predictors, potential confounders, and effect modifiers. Give diagnostic criteria, if applicable | p. 6-10 |
| Data sources/ measurement | 8* | For each variable of interest, give sources of data and details of methods of assessment (measurement). Describe comparability of assessment methods if there is more than one group | p. 6-10 |
| Bias | 9 | Describe any efforts to address potential sources of bias | p. 6-11 |
| Study size | 10 | Explain how the study size was arrived at | p. 7, Figure 1 |
| Quantitative variables | 11 | Explain how quantitative variables were handled in the analyses. If applicable, describe which groupings were chosen and why | p. 6-11 |
| Statistical methods | 12 | (*a*) Describe all statistical methods, including those used to control for confounding<br>(*b*) Describe any methods used to examine subgroups and interactions<br>(*c*) Explain how missing data were addressed<br>(*d*) If applicable, explain how loss to follow-up was addressed<br>(*e*) Describe any sensitivity analyses | p. 10-11 |
| **Results** | | | |
| Participants | 13* | (a) Report numbers of individuals at each stage of study—eg numbers potentially eligible, examined for eligibility, confirmed eligible, included in the study, completing follow-up, and analysed<br>(b) Give reasons for non-participation at each stage<br>(c) Consider use of a flow diagram | p. 7, Figure 1 |
| Descriptive data | 14* | (a) Give characteristics of study participants (eg demographic, clinical, social) and information on exposures and potential confounders | p. 11-12, Table 1 + Table S1 |

For peer review only



For peer review only - http://bmjopen.bmj.com/site/about/guidelines.xhtml

| | | | |
|---|---|---|---|
| | | (b) Indicate number of participants with missing data for each variable of interest<br>(c) Summarise follow-up time (eg, average and total amount) | |
| Outcome data | 15* | Report numbers of outcome events or summary measures over time | p. 11-13, Figure 1 |

For peer review only


For peer review only - http://bmjopen.bmj.com/site/about/guidelines.xhtml

| | | | |
|---|---|---|---|
| Main results | 16 | (*a*) Give unadjusted estimates and, if applicable, confounder-adjusted estimates and their precision (eg, 95% confidence interval). Make clear which confounders were adjusted for and why they were included<br><br>(*b*) Report category boundaries when continuous variables were categorized<br>(*c*) If relevant, consider translating estimates of relative risk into absolute risk for a meaningful time period | p. 12-13, Table 2 |
| Other analyses | 17 | Report other analyses done—eg analyses of subgroups and interactions, and sensitivity analyses | p. 11-12, Table 3 + Table 4 + Table S2 + Table S3 + Table S4 + Table S5 + Table S6 |
| **Discussion** | | | |
| Key results | 18 | Summarise key results with reference to study objectives | p. 13 |
| Limitations | 19 | Discuss limitations of the study, taking into account sources of potential bias or imprecision. Discuss both direction and magnitude of any potential bias | p. 14-15 |
| Interpretation | 20 | Give a cautious overall interpretation of results considering objectives, limitations, multiplicity of analyses, results from similar studies, and other relevant evidence | p. 13-15 |
| Generalisability | 21 | Discuss the generalisability (external validity) of the study results | p. 15 |
| **Other information** | | | |
| Funding | 22 | Give the source of funding and the role of the funders for the present study and, if applicable, for the original study on which the present article is based | p. 16 |

*Give information separately for exposed and unexposed groups.

**Note:** An Explanation and Elaboration article discusses each checklist item and gives methodological background and published examples of transparent reporting. The STROBE checklist is best used in conjunction with this article (freely available on the Web sites of PLoS Medicine at http://www.plosmedicine.org/, Annals of Internal Medicine at http://www.annals.org/, and Epidemiology at http://www.epidem.com/). Information on the STROBE Initiative is available at http://www.strobe-statement.org.

For peer review only
